# Glucagon-like peptide-1 receptor activation and mental health: a drug-target mendelian randomization study

**DOI:** 10.1038/s41398-026-04244-7

**Published:** 2026-07-13

**Authors:** Guoyi Yang, Stephen Burgess, C. Mary Schooling

**Affiliations:** 1 School of Public Health, Li Ka Shing Faculty of Medicine, The University of Hong Kong, Hong Kong, China; 2 Division of Translational Medicine and Human Genetics, Department of Medicine, Perelman School of Medicine, University of Pennsylvania, Philadelphia, PA USA; 3 MRC Biostatistics Unit, University of Cambridge, Cambridge, UK; 4 British Heart Foundation Cardiovascular Epidemiology Unit, Department of Public Health and Primary Care, University of Cambridge, Cambridge, UK; 5 Graduate School of Public Health and Health Policy, City University of New York, New York, NY USA

**Keywords:** Bipolar disorder, Depression, Clinical genetics

## Abstract

Concerns have been raised about the psychiatric safety of glucagon-like peptide-1 receptor (GLP-1R) agonists, but trial evidence suggests that they ameliorate depressive symptoms. We aimed to assess the associations of GLP-1R activation with mental health well-being and the risk of mental health disorders and substance use disorders. We performed drug-target Mendelian randomization and colocalization analyses using the largest relevant genome-wide association studies and replicated in FinnGen. After correcting for multiple comparisons, genetically predicted lower body mass index (BMI) via GLP-1R activation was associated with a better well-being spectrum (0.06 standard deviation [95% confidence interval 0.03-0.08]), lower risk of major depressive disorder (odds ratio 0.82 [0.75-0.88]), and lower risk of bipolar disorder (odds ratio 0.61 [0.47-0.79]) per 1-kg/m^2^ decrease in BMI. There was also suggestive evidence that genetically predicted lower BMI via GLP-1R activation was associated with lower risk of substance use disorders. These associations were stronger than the associations for genetically predicted lower BMI and lower glycated hemoglobin (HbA1c) based on genome-wide variants. The posterior probabilities of colocalization of BMI and each significant outcome within 1 Mb of the *GLP1R* gene were 59.3% for the well-being spectrum, 76.9% for major depressive disorder, and 45.9% for bipolar disorder. The posterior probabilities of colocalization were > 80% for the well-being spectrum and bipolar disorder when conditioning on the presence of a variant associated with the outcome. This study provides genetic evidence that GLP-1R activation is associated with better mental health well-being, lower risk of major depressive disorder, and lower risk of bipolar disorder, possibly beyond its effect on BMI and HbA1c.

## Introduction

Glucagon-like peptide-1 receptor (GLP-1R) agonists reduce body weight and blood glucose [1]. Randomized controlled trials (RCTs) have shown cardiovascular and mortality benefits of GLP-1R agonists [2, 3]. GLP-1R agonists act on the hindbrain, the hypothalamus, and vagal afferents to suppress food intake and reduce body weight [4–6]. Anti-obesity drugs affecting central nervous systems could have psychiatric effects. For example, the combination of phentermine and topiramate increases depression and anxiety [7]. Rimonabant was withdrawn from the market due to serious psychiatric adverse events [8]. In contrast, the combination of naltrexone and bupropion improves depression [9] and substance use disorder [10].

Concerns have been raised about the risk of suicidal ideation and self-injury in people using GLP-1R agonists [11]. However, RCTs showed that GLP-1R agonists moderately reduced depressive symptoms in people who are overweight or obese [12] and in people with type 2 diabetes [13]. Animal [14, 15], observational [16, 17], and genetic studies [18] suggested potential benefits of GLP-1R agonists in alcohol use disorder, but RCTs yielded contradictory evidence showing benefits or null effects [19, 20]. Early-stage trials are on-going to investigate the efficacy of GLP-1R agonists in individuals with mental illnesses [21] and substance use disorders [22]. A better understanding of the association of GLP-1R activation, the intended target of GLP-1R agonists, with mental health has implications for informing their psychiatric safety and potential repurposing opportunities.

To address this gap, we used Mendelian randomization (MR), an instrumental variable analysis with genetic instruments, to obtain less confounded estimates than conventional observational studies [23]. We used MR to assess the associations of GLP-1R activation with mental health outcomes, including the well-being spectrum (comprising life satisfaction, positive affect, neuroticism, and depressive symptoms), mental health disorders, and substance use disorders. We further investigated whether any associations were explained by lowering body mass index (BMI) or lowering glycated hemoglobin (HbA1c).

## Subjects and methods

### Ethics approval and consent to participate

This study was conducted using publicly available summary statistics of genome-wide association studies (GWASs) and did not involve individual-level human data. Consequently, ethical approval was not required for this secondary analysis. All cohorts contributing to the original GWASs obtained ethical approval from their respective institutional review boards or local ethics committees, and all participants provided informed consent.

### Study design

MR relies on the instrumental variable assumptions of relevance (the genetic instruments should be related to the exposure), independence (there is no open backdoor path linking the genetic instruments and the outcome), and exclusion restriction (the genetic instruments should only affect the outcome via the exposure) [23]. This MR study took advantage of the largest relevant publicly available GWASs and replicated in FinnGen (eTable 1). First, we assessed the associations of GLP-1R activation predicted by *GLP1R* variants with mental health outcomes, including the well-being spectrum, mental health disorders, and substance use disorders. We compared these associations with the associations for lower BMI and lower HbA1c predicted by genome-wide variants. Second, we performed colocalization analyses to examine whether any associations found for GLP-1R activation were driven by a shared causal variant affecting both BMI or HbA1c and the outcome in or near the *GLP1R* gene [24]. Where possible, we conducted sex-specific analyses, because women are more likely to have depression than men [25], whereas boys are more likely to develop neurodevelopmental disorders than girls [26].

### Genetic instruments

We selected genetic instruments for GLP-1R activation based on their associations with downstream traits related to drug target effects (BMI and HbA1c), because this approach reduces the risk of the variant-outcome association being lost or confounded by pleiotropic effects [27]. We obtained genetic associations with BMI (*N* = 806,834) from a meta-analysis of the UK Biobank and GIANT [28] and with HbA1c (*N* = 344,182) from a GWAS of the UK Biobank [29]. We considered these two sets of instruments separately, because they might represent distinct mechanisms [30]. For primary analyses, we extracted variants in or near (+- 1 Mb) the *GLP1R* gene that were uncorrelated (r^2^ < 0.001) and genome-wide significantly (*p* value < 5×10^-8^) associated with BMI to mimic BMI-related mechanisms. We used the same approach based on their associations with HbA1c to select genetic variants mimicking HbA1c-related mechanisms. Linkage disequilibrium was obtained from the 1000 Genomes reference panel in individuals of European ancestry. For secondary analyses, we used a missense variant in *GLP1R*, rs10305492 (A316T), which has been associated with fasting glucose and type 2 diabetes [31] and has known functional consequences for GLP-1R signaling [32]. We evaluated the associations of these genetic instruments with *GLP1R* expression in the ten most highly expressed tissues using expression quantitative trait locus (eQTL) data from GTEx v10 [33], and with plasma GLP-1R levels using protein quantitative trait locus (pQTL) data from the UK Biobank Pharma Proteomics Project [34]. For comparison, we also extracted genetic instruments for BMI and HbA1c from across the genome (genome-wide variants) that were uncorrelated (r^2^ < 0.001) and genome-wide significantly (*p* value < 5×10^-8^) associated with BMI and HbA1c, respectively.

We assessed the associations of genetically predicted GLP-1R activation with coronary artery disease (CAD) and all-cause mortality because RCTs have shown that GLP-1R agonists reduce cardiovascular events and death [2, 3]. We obtained genetic associations with CAD (181,522 cases/984,168 controls) from a meta-analysis of the CARDIoGRAMplusC4D consortium and the UK Biobank [35]. We used parental mortality as a proxy for all-cause mortality because it has greater statistical power and gives results consistent with participant’s mortality [36]. We obtained genetic associations with parental mortality (609,139 cases/403,101 controls), determined by parental attained age and their alive/dead status, from a meta-analysis of the UK Biobank and LifeGen [37].

### Genetic associations with mental health outcomes

We obtained sex-combined and sex-specific genetic associations with mental health outcomes from the largest relevant publicly available GWASs (eTable 1) [29, 38–52]. Primary outcomes included the well-being spectrum, mental health disorders (major depressive disorder, bipolar disorder, post-traumatic stress disorder (PTSD), schizophrenia, anorexia nervosa, attention deficit hyperactivity disorder (ADHD), autism spectrum disorder, and Tourette’s syndrome), and substance use disorders. Secondary outcomes included four dimensions of mental health well-being (life satisfaction, positive affect, neuroticism, and depressive symptoms) and subtypes of mental health disorders (postpartum depression and bipolar disorder I and II) and substance use disorders (cannabis use disorder and alcohol dependence). Secondary outcomes also included the Alcohol Use Disorders Identification Test (AUDIT) total score, score for alcohol consumption, and score for alcohol problems. We obtained genetic associations with relevant outcomes from the FinnGen R12 for replication [53], where possible. Genetic associations with binary outcomes obtained using linear regression were transformed into odds ratio (OR) using an established approximation [54].

### MR analysis

We used the F-statistic to assess instrument strength, approximated by the square of SNP-exposure association divided by the square of its standard error [55]. An F-statistic larger than 10 suggests weak instrument bias is unlikely. We aligned SNPs on the same allele for exposure and outcome, and used proxy SNPs (r^2^ > 0.8), where possible, when SNPs were not available in the outcome GWAS. We calculated Wald estimates by dividing genetic association with outcome by genetic association with exposure. We obtained MR estimates by meta-analyzing Wald estimates using inverse variance weighting (IVW) with fixed effects for three SNPs or fewer and random effects for four SNPs or more [56]. To assess the robustness of IVW estimates, we conducted sensitivity analyses using the weighted median [57], MR Egger [58], and MR using the robust adjusted profile score (MR-RAPS) [59] for the genome-wide analyses. We did not perform these sensitivity analyses for *GLP1R*-specific analyses due to the limited number of instruments. We used the Cochran’s Q statistic [60] to test heterogeneity in Wald estimates and used the MR Egger intercept [58] to assess directional pleiotropy. We used the Steiger filtering to identify the SNPs explaining more variance in the outcome than in the exposure (*p* value < 0.05) [61]. We conducted sensitivity analyses excluding the SNPs identified to assess whether MR estimates were affected by reverse causation. We used a Bonferroni-corrected significance level of α = 0.05/10 = 0.005 given ten primary outcomes. We considered nominally significant associations (*p* value < 0.05) that did not reach Bonferroni-corrected significance as suggestive evidence. We tested differences by sex using a two-sided z-test [62].

### Colocalization

We performed pairwise colocalization analyses in a Bayesian framework to assess whether any associations found for GLP-1R activation were driven by a shared causal variant affecting both BMI or HbA1c and the outcome or were confounded by linkage disequilibrium [24]. We included variants (minor allele frequency >0.1%) in or near (+- 1 Mb) the *GLP1R* gene. We set the prior probabilities as recommended, that is 10^-4^ for a variant associated with BMI or HbA1c, 10^-4^ for a variant associated with the outcome, and 10^-5^ for a variant associated with both traits [24]. We assessed the posterior probability of several hypotheses, including H_0_ (no association with either trait), H_1_, (association with BMI or HbA1c only); H_2_, (association with the outcome only); H_3_, (associations of two independent variants and one for each trait); H_4_ (associations of one shared variant with both traits) [24]. A posterior probability of H_4_ larger than 0.80 suggests colocalization [24]. The power to detect colocalization is low when the variants are not strongly associated with the outcome, so we also calculated the posterior probability of colocalization (H_4_) conditional on the presence of a variant associated with the outcome as H_4_/(H_2_ + H_3_ + H_4_) [63]. We reported the posterior probabilities of H_0_-H_4_ and conditional H_4_, as well as the variant with the largest posterior probability of being the shared causal variant.

We also used the Hypothesis Prioritisation for multi-trait Colocalization (HyPrColoc) [64] to assess colocalization across multiple traits identified for GLP-1R activation. We set the prior probability for a variant associated with at least one trait as 10^-4^ and the conditional prior probability for a variant associated with an additional trait given that the variant is associated with at least one other trait as 0.02 [64]. We reported clusters of putatively colocalized traits and the posterior probability of colocalization.

## Results

### Genetic instruments

For primary analyses, we used genetic variants within 1 Mb of *GLP1R* to predict the impact of GLP-1R activation as indicated by lower BMI (rs17757975 and rs4714290) and lower HbA1c (rs10305518) (eFigure 1 and eTable 2). For secondary analyses, we used a missense variant (rs10305492) in *GLP1R* to predict the impact of GLP-1R activation as indicated by lower HbA1c (eFigure 1 and eTable 2). The F-statistics were all >10, suggesting minimal weak instrument bias. These genetic instruments were supported by eQTL or pQTL evidence: rs17757975 was associated with *GLP1R* expression in the heart (atrial appendage), brain (caudate), and the tibial nerve; rs4714290 was associated with *GLP1R* expression in the pancreas; rs10305518 was associated with plasma GLP-1R levels (all *p* values < 0.05; eFigure 2 and eTable 3). The *GLP1R* SNPs for BMI were not associated with HbA1c and vice versa (eTable 4), which substantiated that these two sets of genetic instruments represent distinct mechanisms of GLP-1R activation [30]. For comparison, we also extracted 516 (overall), 303 (women), and 256 (men) SNPs for BMI and 318 (overall), 200 (women), 174 (men) SNPs for HbA1c from across the genome (eTable 5).

Genetically predicted lower BMI and lower HbA1c via GLP-1R activation were associated with lower CAD risk and all-cause mortality, despite wide confidence intervals (CIs) (eFigure 3). The point estimates for GLP-1R activation were comparable to those for genetically predicted lower BMI and lower HbA1c based on genome-wide variants (eFigure 3 and eTable 6).

### Associations of GLP-1R activation with mental health well-being

**Fig. 1:**
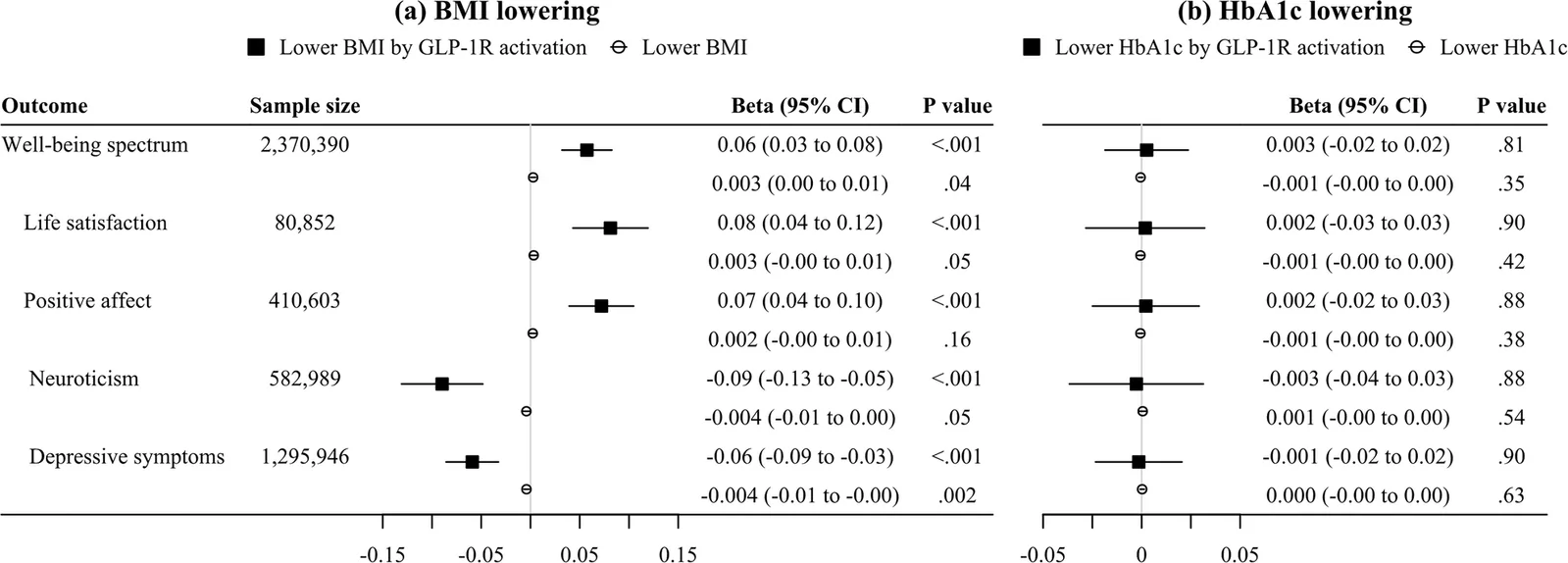
IVW MR estimates for the associations of GLP-1R activation with mental health well-being. BMI, body mass index; GLP-1R, glucagon-like peptide-1 receptor; HbA1c, glycated hemoglobin; IVW, inverse variance weighted; MR, Mendelian randomization. Black squares denote genetically predicted (**a**) lower BMI or (**b**) lower HbA1c via GLP-1R activation based on variants within 1 Mb of *GLP1R*. White circles denote genetically predicted (**a**) lower BMI or (**b**) lower HbA1c based on genome-wide variants. Positive associations with the well-being spectrum, life satisfaction, and positive affect and negative associations with neuroticism and depressive symptoms indicate better mental health well-being. Estimates are presented in standard deviation units for mental health well-being per 1-kg/m^2^ decrease in BMI or per 1-mmol/mol decrease in HbA1c.

Genetically predicted lower BMI via GLP-1R activation was associated with a better well-being spectrum, including life satisfaction, positive affect, neuroticism, and depressive symptoms (Fig. 1). This association with depressive symptoms was evident specifically in women (eFigure 4). These associations were stronger than the associations for genetically predicted lower BMI using genome-wide variants (Fig. 1). However, genetically predicted lower HbA1c via GLP-1R activation had little association with mental health well-being (Fig. 1). Sensitivity analyses using the weighted median, MR Egger, and MR-RAPS gave consistent estimates (eTable 7). The Steiger filtering identified no SNP that explained more variance in the outcome than in the exposure.

### Associations of GLP-1R activation with the risk of mental health disorders

**Fig. 2:**
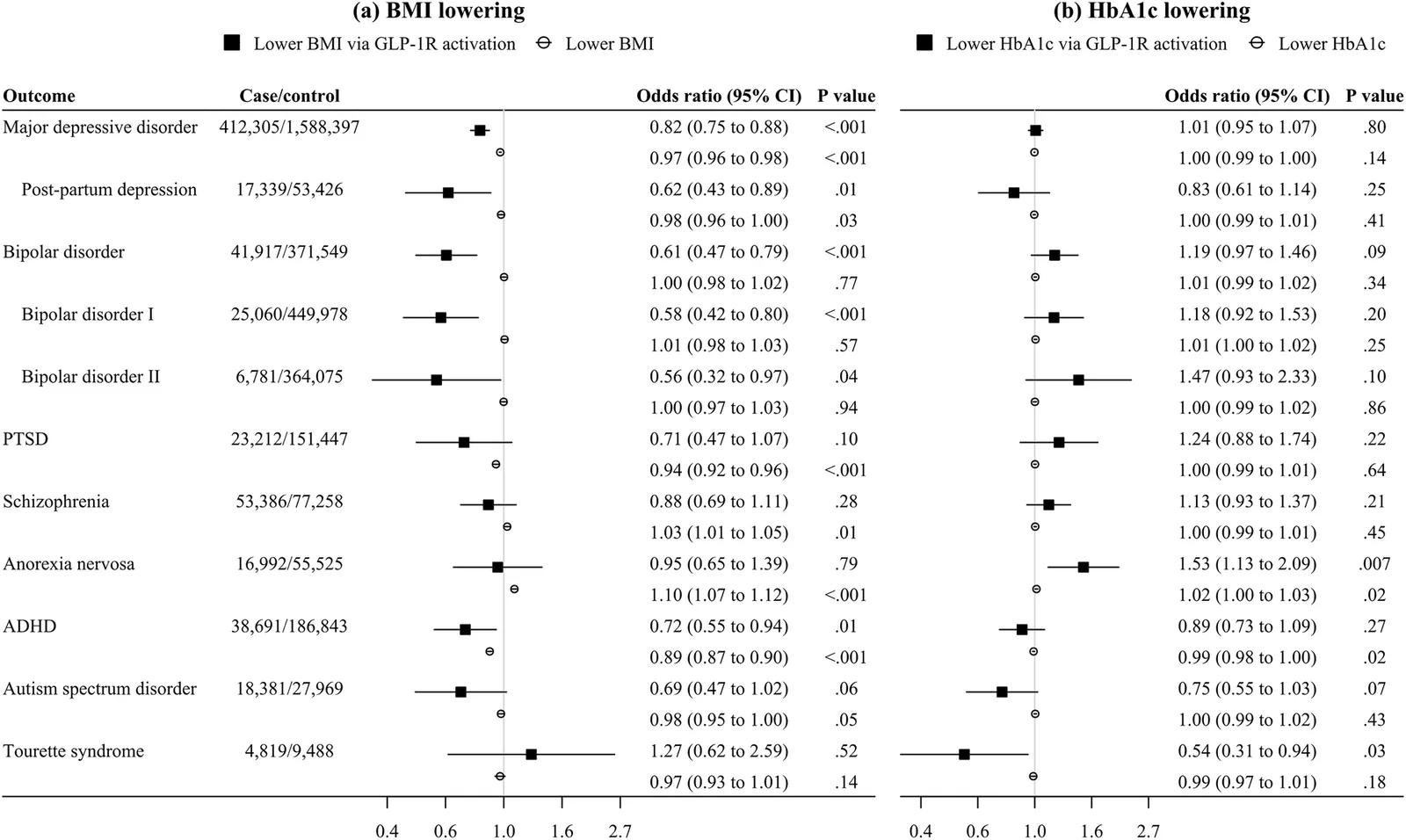
IVW MR estimates for the associations of GLP-1R activation with the risk of mental health disorders. ADHD, attention deficit hyperactivity disorder; BMI, body mass index; GLP-1R, glucagon-like peptide-1 receptor; HbA1c, glycated hemoglobin; IVW, inverse variance weighted; MR, Mendelian randomization; PTSD, post-traumatic stress disorder. Black squares denote genetically predicted (**a**) lower BMI or (**b**) lower HbA1c via GLP-1R activation based on variants within 1 Mb of *GLP1R*. White circles denote genetically predicted (**a**) lower BMI or (**b**) lower HbA1c based on genome-wide variants. Estimates are presented as odds ratio per 1-kg/m^2^ decrease in BMI or per 1-mmol/mol decrease in HbA1c.

Genetically predicted lower BMI via GLP-1R activation was associated with lower risk of major depressive disorder, postpartum depression, bipolar disorder, bipolar disorder I and II, and ADHD (Fig. 2). This association with major depressive disorder was possibly stronger in women (odds ratio 0.70 [95% confidence intervals 0.59-0.82]) than men (0.84 [0.71-1.01]), although the *p* value for sex difference was 0.12 (eFigure 5). These associations were stronger than the associations for genetically predicted lower BMI using genome-wide variants (Fig. 2). Genetically predicted lower HbA1c via GLP-1R activation was associated with higher risk of anorexia nervosa but lower risk of Tourette syndrome, with stronger associations than the associations for genetically predicted lower HbA1c using genome-wide variants (Fig. 2). These results were robust to different MR methods and the exclusion of SNPs identified by the Steiger filtering (eTable 8).

### Associations of GLP-1R activation with the risk of substance use disorders

**Fig. 3:**
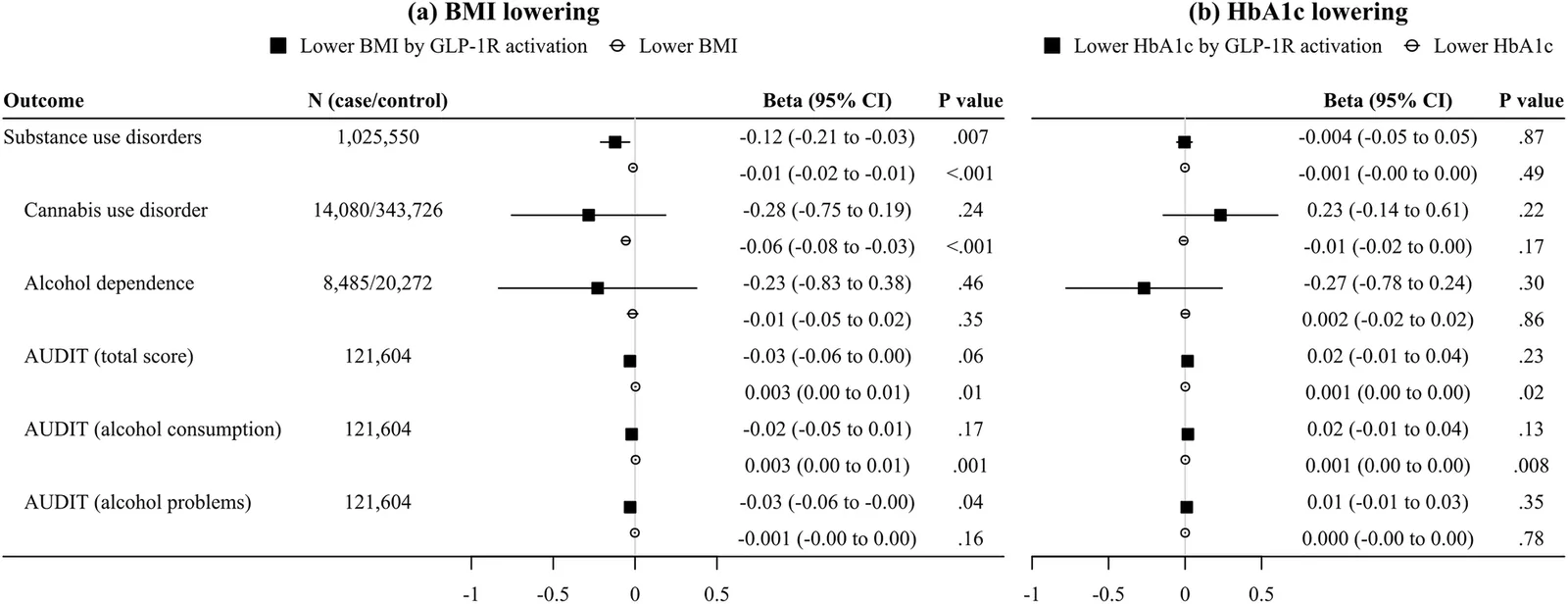
IVW MR estimates for the associations of GLP-1R activation with the risk of substance use disorders and AUDIT scores. AUDIT, Alcohol Use Disorders Identification Test; BMI, body mass index; GLP-1R, glucagon-like peptide-1 receptor; HbA1c, glycated hemoglobin; IVW, inverse variance weighted; MR, Mendelian randomization. Black squares denote genetically predicted (**a**) lower BMI or (**b**) lower HbA1c via GLP-1R activation based on variants within 1 Mb of *GLP1R*. White circles denote genetically predicted (**a**) lower BMI or (**b**) lower HbA1c based on genome-wide variants. Positive associations with AUDIT scores indicate higher risk of alcohol use disorders. Estimates are presented in standard deviation unit for substance use disorder risk, as log odds for cannabis use disorder and alcohol dependence, and as log10 transformed score for AUDIT scores per 1-kg/m^2^ decrease in BMI or per 1-mmol/mol decrease in HbA1c.

Genetically predicted lower BMI via GLP-1R activation was associated with lower risk of substance use disorders and lower AUDIT score for alcohol problems (Fig. 3). These associations were stronger than the associations for genetically predicted lower BMI using genome-wide variants. However, genetically predicted lower HbA1c via GLP-1R activation had little association with the risk of substance use disorders (Fig. 3). Sensitivity analyses using different MR methods and excluding the SNPs identified by the Steiger filtering gave similar interpretations (eTable 9).

### Replication in FinnGen

In FinnGen, genetically predicted lower BMI via GLP-1R activation was associated with lower risk of major depressive disorder, bipolar disorder, PTSD, ADHD, substance abuse, and alcohol dependence (eFigure 6). Again, these associations were stronger than the associations for genetically lower BMI using genome-wide variants (eTable 10). In contrast, genetically predicted lower HbA1c via GLP-1R activation had little association with mental health outcomes except for major depressive disorder (eFigure 6).

### Associations of the GLP1R missense variant with mental health outcomes

As the *GLP1R* missense variant (rs10305492, A316T) was genome-wide significantly associated with HbA1c but had little impact on BMI (eTable 4), we used it to proxy lower HbA1c via GLP-1R activation. Although rs10305492 was not significantly associated with CAD or all-cause mortality, it showed effect magnitudes and directions consistent with the primary analyses (eTable 11). This specific variant was not available in the GWAS for the well-being spectrum and showed little association with mental health disorders or substance use disorders (eTable 11).

### Colocalization

**Fig. 4:**
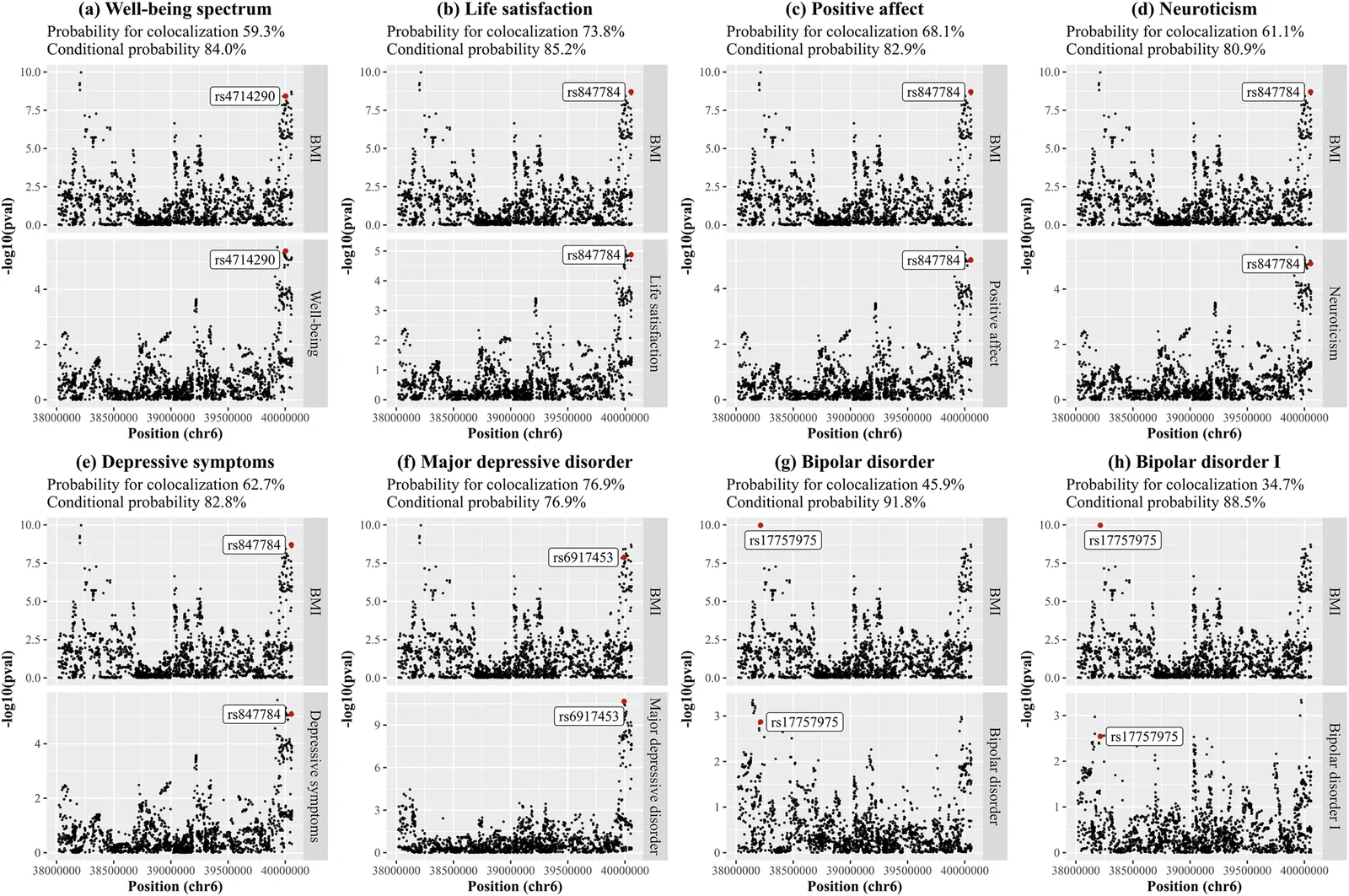
Pairwise colocalization analyses for BMI and each significant outcome within 1 Mb of the *GLP1R* gene. BMI, body mass index. Panels illustrate pairwise colocalization results for BMI and (**a**) the well-being spectrum, (**b**) life satisfaction, (**c**) positive affect, (**d**) neuroticism, (**e**) depressive symptoms, (**f**) major depressive disorder, (**g**) bipolar disorder, and (**h**) bipolar disorder I. Probability for colocalization means the posterior probability of a shared variant associated with both traits; conditional probability means the posterior probability of a shared variant associated with both traits conditional on the presence of a variant associated with the outcome. Given the presence of a shared variant associated with both traits, the labeled variant had the largest probability of being the shared causal variant.

After correcting for multiple comparisons, genetically predicted lower BMI via GLP-1R activation remained significantly associated with the well-being spectrum, its four dimensions (life satisfaction, positive affect, neuroticism, depressive symptoms), major depressive disorder, bipolar disorder, and bipolar disorder I (*p* values < 0.005). Pairwise colocalization analyses were performed for BMI and each significant outcome within 1 Mb of the *GLP1R* gene. The posterior probabilities of colocalization were 59.3% for the well-being spectrum, 76.9% for major depressive disorder, and 45.9% for bipolar disorder (Fig. 4). Conditioning on the presence of a variant associated with the outcome, the posterior probabilities of colocalization were >80% for all the significant outcomes except for major depressive disorder (Fig. 4), suggesting that some lack of colocalization was due to limited power. The posterior probabilities of two independent variants associated with each trait were <25% for all the significant outcomes (eTable 12), suggesting that confounding by linkage disequilibrium was unlikely. Within 1 Mb of the *GLP1R* gene, rs4714290, its proxies (rs847784 and rs6917453, r^2^ > 0.80), or rs17757975 had the largest posterior probability of being the shared causal variant, which substantiated their validity as genetic instruments.

Multi-trait colocalization analyses were performed across BMI and all the significant outcomes within 1 Mb of the *GLP1R* gene. We identified BMI, the well-being spectrum, life satisfaction, positive affect, neuroticism, depressive symptoms, and major depressive disorder as a cluster of putatively colocalized traits with the posterior probability of colocalization 51.5%.

## Discussion

This drug-target MR study suggests that GLP-1R activation is associated with a better well-being spectrum, lower risk of major depressive disorder, and lower risk of bipolar disorder. We also provide suggestive evidence that GLP-1R activation is associated with lower risk of substance use disorders. The associations of GLP-1R activation with favorable mental health outcomes exceeded those expected from general reduction of BMI or HbA1c.

We found that genetically predicted GLP-1R activation was associated with a better well-being spectrum, lower risk of major depressive disorder, and lower risk of bipolar disorder. Consistently, RCTs have shown that GLP-1R agonists moderately reduce depression symptoms in overweight people [12] and in people with type 2 diabetes [13]. A small open-label trial showed that liraglutide, as an adjunct to existing pharmacotherapy, improved cognitive function [65] and brain volumes [66] in people with depression or bipolar disorder. These psychiatric benefits of GLP-1R activation could be driven by the neuroprotective effects of GLP-1 including enhanced neurogenesis, improved barrier function, reduced neuroinflammation, and decreased oxidative stress [67].

Interestingly, the associations of GLP-1R activation with favorable mental health outcomes were more evident when proxied via BMI than via HbA1c. Furthermore, the *GLP1R* missense variant, which affects glycemic traits but not BMI, showed null associations with mental health outcomes. These findings underscore the central role of BMI-related mechanisms in the psychiatric benefits of GLP-1R activation. A recent study showed that GLP-1R agonists target the hindbrain dorsal vagal complex to decrease food intake and body weight [4]. Actions on the hypothalamus and vagal afferents also contribute to the weight loss on GLP-1R agonists [5, 6]. GLP neurons in the dorsal vagal complex moderate stress responses and stimulate hypothalamic-pituitary-adrenal (HPA) axis activity, which has been implicated in the pathology of depression [68] and bipolar disorder [69]. Notably, the HPA axis is also related to cognitive function [68, 69], consistent with an RCT showing that dulaglutide ameliorates cognitive impairment in people with type 2 diabetes [70]. Additional studies are warranted to elucidate the mechanisms underlying potential benefits of GLP-1R agonists for mental health.

We found that genetically predicted GLP-1R activation was associated with lower risk of major depressive disorder specifically in women and with lower risk of postpartum depression. We caution that the power to detect sex differences may have been limited by small or unequal sample sizes of sex-stratified GWAS. Women are about twice as likely as men to have depression during their lifetime with the difference starting at puberty [25]. A meta-analysis of RCTs showed that GLP-1R agonists decreased testosterone and increased sex hormone-binding globulin in women with polycystic ovary syndrome [71], but their effects on estrogen are less clear. Sex hormones might contribute to GLP-1R activation improving depression in women, given their potential roles in modulating serotonergic neurotransmission, cortisol, and oxytocin [72].

Genetically predicted GLP-1R activation was suggestively associated with lower risk of ADHD and Tourette syndrome but had a null association with autism spectrum disorder. Although RCTs have shown the short-term (no more than 26 weeks) safety of GLP-1R agonists in children and adolescents [73], RCTs typically do not capture the long latency of neurodevelopmental disorders. Our study adds to the evidence by showing little adverse effect of lifelong GLP-1R activation on these disorders. However, GLP-1R activation was associated with higher risk of anorexia nervosa when proxied via HbA1c. GLP-1R activation increases insulin secretion and sensitivity and lowers glucose [74]. A previous MR study suggested an inverse association of fasting insulin with the risk of anorexia nervosa [75], indicating a role of insulin sensitivity.

We provide suggestive evidence that GLP-1R activation was associated with lower risk of substance use disorders and lower AUDIT score for alcohol problems, which were replicated in FinnGen. Our findings align with a recent RCT showing that eight-week treatment with semaglutide reduced alcohol consumption and craving in people with alcohol use disorder [20]. In contrast, another RCT including people with alcohol use disorder showed that 26 weeks of treatment with exenatide (an GLP-1R agonist) did not significantly reduce heavy drinking days, although exenatide attenuated fMRI alcohol cue reactivity in brain areas for drug reward and addiction [19]. Further evidence from RCTs is needed to determine the repurposing opportunities of GLP-1R agonists for treating substance use disorders.

We presented the MR estimates of GLP-1R activation in effect sizes of BMI or HbA1c reduction for interpretability. However, this scaling does not imply that weight loss or glucose lowering is the sole mechanism of action for the psychiatric effects of GLP-1R activation. Indeed, the associations of GLP-1R activation with mental health outcomes appeared stronger than those expected from lowering BMI or lowering HbA1c. Correspondingly, RCTs have showed that semaglutide reduces cardiovascular events and mortality more than expected from the reduction in body weight [12], and that GLP-1R agonists outperform other anti-diabetes drugs [76]. Apart from reducing body weight and glucose, GLP-1R agonists also decrease blood pressure, low-density lipoprotein cholesterol, and inflammation and enhance kidney function [2, 3]. These multifaceted effects of GLP-1R agonists could be partially explained by 5’ adenosine monophosphate-activated protein kinase (AMPK) activation [77]. Taken together, our findings suggest that the psychiatric benefits of GLP-1R activation likely extend beyond its effect on BMI and HbA1c.

This drug-target MR study comprehensively explored the associations of GLP-1R activation with mental health outcomes. Our investigation has implications for the psychiatric safety and repurposing opportunities of GLP-1R agonists, particularly in people with mental health disorders, who usually have higher risk of obesity and type 2 diabetes but are undertreated. This study has several limitations. First, MR relies on three rigorous assumptions, that is genetic instruments should be related to the exposure, share no open backdoor path with the outcome, and only affect the outcome via the exposure [23]. The F-statistics were >10, suggesting minimal weak instrument bias. To validate the genetic instruments, we evaluated their associations with tissue-specific *GLP1R* expression and plasma GLP-1R levels, as well as with CAD and all-cause mortality. Second, we selected genetic variants within 1 Mb of the *GLP1R* gene to proxy GLP-1R activation based on their associations with BMI and HbA1c. However, we cannot exclude the possibility that the observed psychiatric effects are driven by pleiotropy from neighboring genes. The primary instruments selected based on BMI are not within the *GLP1R* gene, and other genes are closer to these variants (eFigure 1). Furthermore, instrumenting on BMI and HbA1c could have masked alternative pathways of GLP-1R activation, particularly if additional underlying factors contribute to these outcomes. Third, the small number of genetic instruments for GLP-1R activation constrained the use of MR sensitivity analyses, thereby limiting the assessment of pleiotropy. Fourth, we obtained genetic associations with BMI from a meta-analysis of the UK Biobank and GIANT and with HbA1c from the UK Biobank, which overlaps with some outcome GWASs. Two-sample MR methods in a one-sample setting perform well in terms of bias and precision in large biobanks, except for MR Egger which can result in bias reflecting the direction and magnitude of confounding [78]. We replicated the results using outcome GWASs from FinnGen, which has no overlap with the UK Biobank or GIANT. Fifth, the power to detect colocalization was insufficient. However, the posterior probabilities of two independent variants associated with each trait were <25% for all the significant outcomes, suggesting that the associations found were unlikely driven by confounding due to linkage disequilibrium. Sixth, MR can be open to selection bias from selecting on genetic makeup and mental health disorders. Nevertheless, such selection should bias towards a harmful effect of GLP-1R activation, which cannot explain the observed favorable associations with mental health outcomes. Finally, mental health disorders sometimes originate during childhood or adolescence, but GLP-1R agonists are primarily used in adults. Our MR study assessed the lifelong effects of genetically predicted GLP-1R activation in the general population, which may differ from short-term effects of GLP-1R agonists in patients. RCTs are warranted to determine the clinical relevance of these findings in the future.

## Conclusions

This drug-target MR study provides genetic evidence that GLP-1R activation is associated with better mental health well-being, lower risk of major depressive disorder, and lower risk of bipolar disorder, possibly beyond its effect on BMI and HbA1c.

## Supplementary information

Supplements

## Data Availability

All statistical analyses were conducted using R version 4.2.1 and the packages “ieugwasr”, “TwoSampleMR”, “MendelianRandomization”, “mr.raps, “metafor”, “coloc”, and “hyprcoloc”.

## Data availability

Summary statistics analyzed are available in the website https://doi.org/10.5281/zenodo.1251813 for BMI, http://www.nealelab.is/uk-biobank/ for HbA1c, https://www.gtexportal.org/home/downloads/adult-gtex/qtl for eQTL, https://www.synapse.org/Synapse:syn51364943 for pQTL, https://www.cardiogramplusc4d.org/data-downloads/ for CAD, https://www.ebi.ac.uk/gwas/publications/30642433 for parental mortality, https://www.ebi.ac.uk/gwas/publications/30643256 for mental health well-being, and https://pgc.unc.edu/for-researchers/download-results/, https://www.ebi.ac.uk/gwas/publications/40858613, and https://www.finngen.fi/en/access_results for mental health disorders and substance use disorders.

## Artificial intelligence (AI)-assisted technologies

The authors declared that AI-assisted technologies were not used in this study.
