# Supplementary material for: Glucagon-like peptide-1 receptor activation and mental health: a drug-target mendelian randomization study
Source: Transl Psychiatry. 2026 Jul 13;16:529. doi: 10.1038/s41398-026-04244-7 (PMC13642936; doi:10.1038/s41398-026-04244-7)
Supplement: Supplementary file 1 [file 41398_2026_4244_MOESM1_ESM.pdf]

Online-Only Supplements

eTable 1. A summary of genome-wide association studies used in this study.

eFigure 1. Regional plots for genetic instruments for GLP-1R activation.

eTable 2. SNP-specific estimates for genetic instruments for GLP-1R activation on BMI (kg/m^2) and HbA1c (mmol/mol).

eFigure 2. The associations of genetic instruments for GLP-1R activation with *GLP1R* expression in the ten most highly expressed tissues.

eTable 3. SNP-specific estimates for genetic instruments for GLP-1R activation on plasma GLP-1R levels.

eTable 4. MR estimates for the associations of GLP-1R activation with HbA1c (mmol/mol) and BMI (kg/m^2).

eTable 5. SNP-specific estimates for genetic instruments for BMI (kg/m^2) and HbA1c (mmol/mol).

eFigure 3. IVW MR estimates for the associations of GLP-1R activation with the risk of coronary artery disease and all-cause mortality in comparison with those for lower BMI and lower HbA1c.

eTable 6. MR sensitivity analyses for the associations of lower BMI and lower HbA1c with the risk of coronary artery disease and all-cause mortality.

eFigure 4. IVW MR estimates for sex-specific associations of GLP-1R activation with the risk of depressive symptoms in comparison with those for lower BMI and lower HbA1c.

eTable 7. MR sensitivity analyses for the associations of lower BMI and lower HbA1c with mental health well-being.

eFigure 5. IVW MR estimates for sex-specific associations of GLP-1R activation with the risk of major depressive disorder, schizophrenia, and ADHD in comparison with those for lower BMI and lower HbA1c.

eTable 8. MR sensitivity analyses for the associations of lower BMI and lower HbA1c with the risk of mental health disorders.

eTable 9. MR sensitivity analyses for the associations of lower BMI and lower HbA1c with the risk of substance use disorders.

eFigure 6. IVW MR estimates for the associations of GLP-1R activation with mental health outcomes in comparison with those for lower BMI and lower HbA1c in FinnGen.

eTable 10. MR sensitivity analyses for the associations of lower BMI and lower HbA1c with mental health outcomes in FinnGen.

eTable 11. Wald estimates for the associations of lower HbA1c via GLP-1R activation (instrumented by rs10305492) with coronary artery disease, all-cause mortality, and mental health outcomes.

eTable 12. The posterior probabilities of different hypotheses in pairwise colocalization analyses for BMI and each significant outcome within 1 Mb of the *GLP1R* gene.

eTable 1. A summary of genome-wide association studies used in this study.

Category	Phenotype	Study	Ancestry	Sex	*N* (case/control)	Covariates	PMID
Genetic instruments	Body mass index	UKB, GIANT	European	Overall	806,834	Sex, age at assessment, age at assessment^2, and assessment center (UKB); sex, age, age^2, and study-specific covariates (GIANT)	30239722
	Body mass index	UKB, GIANT	European	Women	434,794	Age at assessment, age at assessment^2, and assessment center (UKB); age, age^2, and study-specific covariates (GIANT)	30239722
	Body mass index	UKB, GIANT	European	Men	374,756	Age at assessment, age at assessment^2, and assessment center (UKB); age, age^2, and study-specific covariates (GIANT)	30239722
	HbA1c	UKB	European	Overall	344,182	Age, age^2, inferred sex, age × inferred sex, age^2 × inferred sex, and the first 20 PCs	Neale lab
	HbA1c	UKB	European	Women	185,022	Age, age^2, and the first 20 PCs	Neale lab
	HbA1c	UKB	European	Men	159,160	Age, age^2, and the first 20 PCs	Neale lab
	Tissue-specific *GLP1R* expression	GTEx v10	Mixed (European ~85%)	Overall	943	The first five PCs, PEER factors, PCR, platform, and sex	32913098
	Plasma GLP-1R level	UKB-PPP	European	Overall	32,867	Age, age^2, sex, age × sex, age^2 × sex, batch, centre, genetic array, time between blood sampling and measurement, and the first 10 PCs	37794186
Cardiovascular and mortality outcome	Coronary artery disease	UKB, CARDIoGRAMplusC4D	Mixed (European >95%)	Overall	181,522/984,168	Study-specific covariates	36474045
	Parental mortality	UKB, LifeGen	European	Overall	609,139/403,101	Genotyping batch and array, the first 40 PCs of relatedness, and participant sex (UKB); participant sex, the first 10 PCs, and study-specific covariates (LifeGen)	30642433

Primary outcome	Well-being spectrum	Meta-analysis	European	Overall	2,370,390	Study-specific covariates	30643256
	Major depressive disorder	PGC excluding 23andMe	European	Overall	412,305/1,588,397	The first four PCs and any other PC that was nominally significantly associated with the phenotype	39814019
	Major depressive disorder	Meta-analysis	European	Women	130,471/159,521	The first ten PCs, a genetic relationship matrix, and study-specific covariates	40858613
	Major depressive disorder	Meta-analysis	European	Men	64,805/132,185	The first ten PCs, a genetic relationship matrix, and study-specific covariates	40858613
	Bipolar disorder	Meta-analysis	European	Overall	41,917/371,549	The first five PCs and any others as required	34002096
	PTSD	PGC, UKB	European	Overall	23,212/151,447	The first five PCs (unrelated subjects); the first five PCs and a genetic relatedness matrix (family and twin studies); the first six PCs, batch, and center indicator variables (UKB)	31594949
	Schizophrenia	PGC	European	Overall	53,386/77,258	The first four PCs and any other PC that was nominally significantly associated with the phenotype	35396580
	Schizophrenia	PGC	European	Women	17,710/36,803	The first four PCs and any other PC that was nominally significantly associated with the phenotype	35396580
	Schizophrenia	PGC	European	Men	33,097/35,190	The first four PCs and any other PC that was nominally significantly associated with the phenotype	35396580
	Anorexia nervosa	PGC, UKB	European	Overall	16,992/55,525	The first five PCs and any other PC that was nominally associated with the phenotype	31308545
	Attention deficit hyperactivity disorder	iPSYCH, deCODE, PGC	European	Overall	38,691/186,843	The first ten PCs (iPSYCH); sex, year of birth, county of origin, population stratification, and relatedness (deCODE); PCs and study-specific covariates (PGC)	36702997

	Attention deficit hyperactivity disorder	iPSYCH, PGC	European	Women	4,945/16,246	The first four PCs and any other PC that was nominally significantly associated with the phenotype (iPSYCH); the first five or ten PCs or indicator variables coding for site ID (PGC)	29325848
	Attention deficit hyperactivity disorder	iPSYCH, PGC	European	Men	14,154/17,948	The first four PCs and any other PC that was nominally significantly associated with the phenotype (iPSYCH); the first five or ten PCs or indicator variables coding for site ID (PGC)	29325848
	Autism spectrum disorder	iPSYCH, PGC	European	Overall	18,381/27,969	The first four PCs and any other PC that was nominally significantly associated with the phenotype (iPSYCH); study-specific covariates (PGC)	30804558
	Tourette's syndrome	Meta-analysis	European	Overall	4,819/9,488	The first four population stratification components and any additional population stratification component that was nominally significantly associated with the phenotype	30818990
	Substance use disorders	Meta-analysis	European	Overall	1,025,550	Study-specific covariates	37250466
Secondary outcome	Life satisfaction	Meta-analysis	European	Overall	80,852	Study-specific covariates	30643256
	Positive affect	Meta-analysis	European	Overall	410,603	Study-specific covariates	30643256
	Neuroticism	Meta-analysis	European	Overall	582,989	Study-specific covariates	30643256
	Depressive symptoms	Meta-analysis	European	Overall	1,295,946	Study-specific covariates	30643256
	Depressive symptoms (seen a general practitioner)	UKB	European	Women	79,976/112,704	Age, age^2, and the first 20 PCs	Neale lab

	Depressive symptoms (seen a general practitioner)	UKB	European	Men	43,552/122,461	Age, age^2, and the first 20 PCs	Neale lab
	Depressive symptoms (seen a psychiatrist)	UKB	European	Women	23,975/169,269	Age, age^2, and the first 20 PCs	Neale lab
	Depressive symptoms (seen a psychiatrist)	UKB	European	Men	17,258/149,033	Age, age^2, and the first 20 PCs	Neale lab
	Postpartum depression	Meta-analysis	European	Women	17,339/53,426	Study-specific covariates	37849304
	Bipolar disorder I	Meta-analysis	European	Overall	25,060/449,978	The first five PCs and any others as required	34002096
	Bipolar disorder II	Meta-analysis	European	Overall	6,781/364,075	The first five PCs and any others as required	34002096
	Cannabis use disorder	iPSYCH, deCODE, PGC	European	Overall	14,080/343,726	Five ancestral PCs, data processing waves, and the presence of another psychiatric disorder (iPSYCH); sex, age, county of origin, population stratification, and relatedness (deCODE); sex and five to ten within-ancestry PCs (PGC)	33096046
	Alcohol dependence	Meta-analysis	European	Overall	8,485/20,272	Sex and PCs	30482948
	AUDIT (total score)	UKB	European	Overall	121,604	Age, sex, genotyping array, and the first 20 PCs	30336701
	AUDIT (alcohol consumption)	UKB	European	Overall	121,604	Age, sex, genotyping array, and the first 20 PCs	30336701
	AUDIT (alcohol problems)	UKB	European	Overall	121,604	Age, sex, genotyping array, and the first 20 PCs	30336701
Replication outcome	Major depressive disorder	FinnGen R12	European	Overall	59,333/434,831	Age, sex, FinnGen chip version 1 or 2, legacy genotyping batch, and the first 10 PCs	36653562
	Bipolar disorder	FinnGen R12	European	Overall	8,946/434,831	Age, sex, FinnGen chip version 1 or 2, legacy genotyping batch, and the first 10 PCs	36653562

PTSD	FinnGen R12	European	Overall	3,444/444,414	Age, sex, FinnGen chip version 1 or 2, legacy genotyping batch, and the first 10 PCs	36653562
Schizophrenia	FinnGen R12	European	Overall	7,234/484,776	Age, sex, FinnGen chip version 1 or 2, legacy genotyping batch, and the first 10 PCs	36653562
Anorexia nervosa (including atypical)	FinnGen R12	European	Overall	2,621/484,901	Age, sex, FinnGen chip version 1 or 2, legacy genotyping batch, and the first 10 PCs	36653562
Attention deficit hyperactivity disorder	FinnGen R12	European	Overall	4,452/490,708	Age, sex, FinnGen chip version 1 or 2, legacy genotyping batch, and the first 10 PCs	36653562
Substance abuse	FinnGen R12	European	Overall	30,806/362,304	Age, sex, FinnGen chip version 1 or 2, legacy genotyping batch, and the first 10 PCs	36653562
Alcohol dependence	FinnGen R12	European	Overall	12,599/468,475	Age, sex, FinnGen chip version 1 or 2, legacy genotyping batch, and the first 10 PCs	36653562

AUDIT, Alcohol Use Disorders Identification Test; CARDIoGRAMplusC4D, Coronary ARtery DIsease Genome wide Replication and Meta-analysis plus The Coronary Artery Disease Genetics; GIANT, Genetic Investigation of ANthropometric Traits; HbA1c, glycated hemoglobin; iPSYCH, Integrative Psychiatric Research; PC, principal component; PCR, polymerase chain reaction; PEER, Probabilistic Estimation of Expression Residuals; PGC, Psychiatric Genomics Consortium; PTSD, post-traumatic stress disorder; UKB, UK Biobank; UKB-PPP, UK Biobank Pharma Proteomics Project.

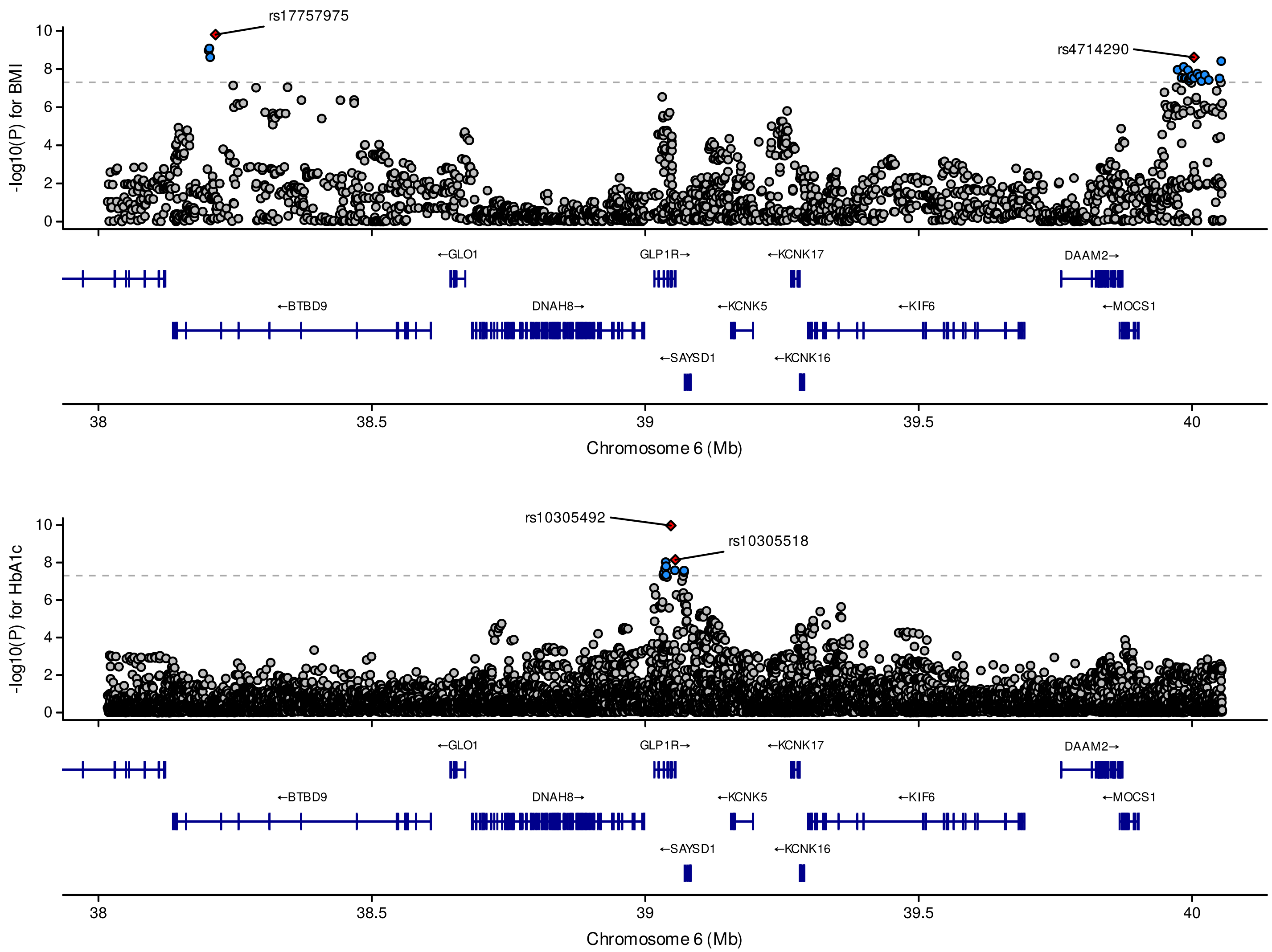

eFigure 1. Regional plots for genetic instruments for GLP-1R activation.

BMI, body mass index; GLP-1R, glucagon-like peptide-1 receptor; HbA1c, glycated hemoglobin.

eTable 2. SNP-specific estimates for genetic instruments for GLP-1R activation on BMI (kg/m^2) and HbA1c (mmol/mol).

Category	Phenotype	Sex	SNP	Effect allele	Other allele	Effect allele frequency	Beta	SE	*P* value	F-statistic
Primary analyses	BMI	Overall	rs17757975	T	C	0.846	0.075	0.012	1.6E-10	41.7
	BMI	Overall	rs4714290	T	C	0.705	0.054	0.009	2.4E-09	34.7
	BMI	Women	rs17757975	T	C	0.845	0.066	0.016	2.3E-05	17.5
	BMI	Women	rs4714290	T	C	0.705	0.056	0.012	4.6E-06	21.5
	BMI	Men	rs17757975	T	C	0.844	0.089	0.017	1.8E-07	26.4
	BMI	Men	rs4714290	T	C	0.704	0.058	0.013	1.1E-05	18.7
	HbA1c	Overall	rs10305518	G	T	0.057	0.195	0.034	7.2E-09	33.5
	HbA1c	Women	rs10305518	G	T	0.057	0.174	0.045	1.1E-04	14.9
	HbA1c	Men	rs10305518	G	T	0.057	0.218	0.051	1.6E-05	18.6
Secondary analyses	HbA1c	Overall	rs10305492	G	A	0.984	0.403	0.062	1.1E-10	41.7
	HbA1c	Women	rs10305492	G	A	0.984	0.270	0.083	1.2E-03	10.5
	HbA1c	Men	rs10305492	G	A	0.984	0.556	0.094	3.9E-09	34.7

BMI, body mass index; GLP-1R, glucagon-like peptide-1 receptor; HbA1c, glycated hemoglobin.

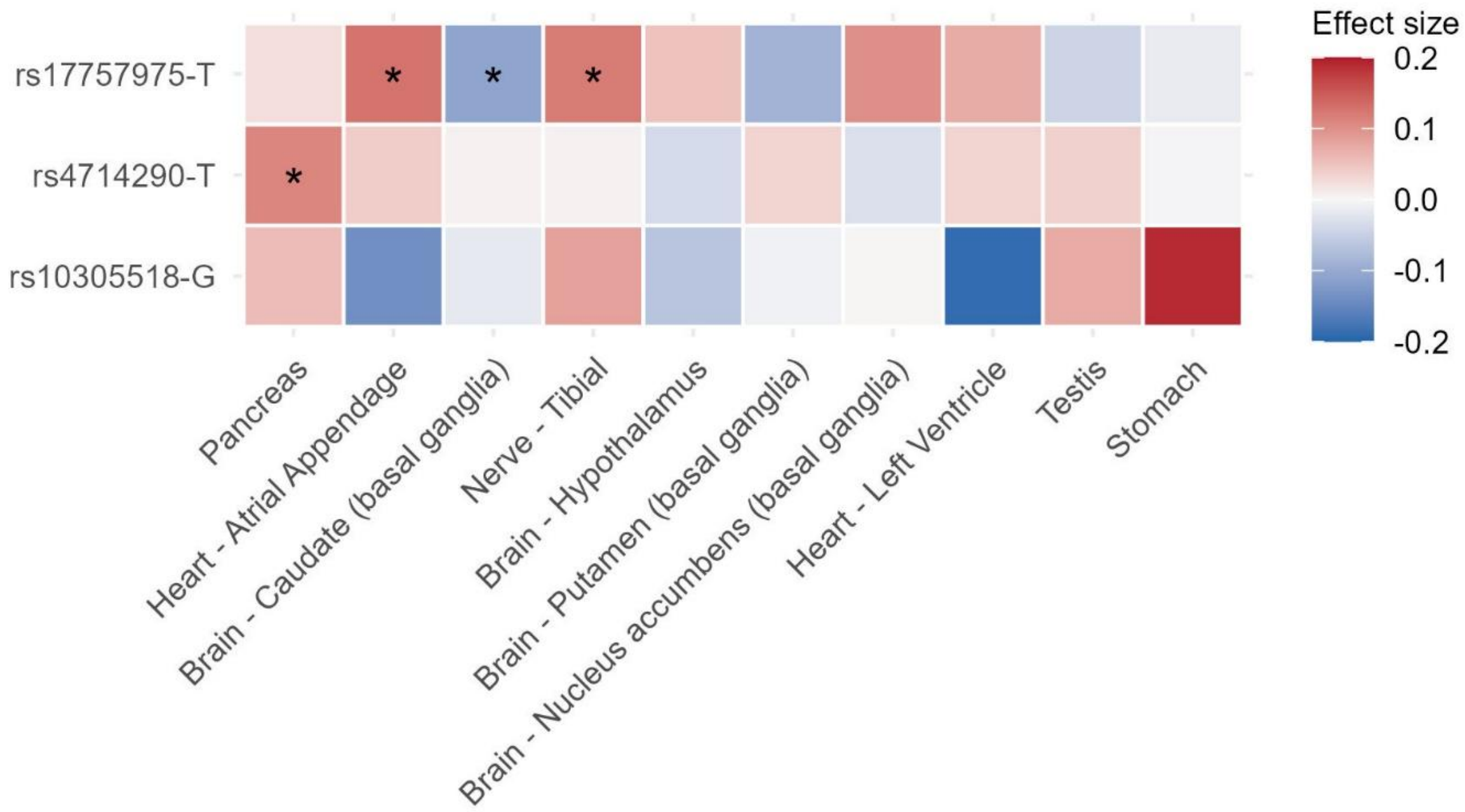

eFigure 2. The associations of genetic instruments for GLP-1R activation with *GLP1R* expression in the ten most highly expressed tissues.

GLP-1R, glucagon-like peptide-1 receptor. rs10305492 was not available in the expression quantitative trait locus data. * denotes p value < 0.05.

eTable 3. SNP-specific estimates for genetic instruments for GLP-1R activation on plasma GLP-1R levels.

Category	Phenotype	SNP	Effect allele	Other allele	Effect allele frequency	Beta	SE	*P* value
Primary analyses	BMI	rs17757975	T	C	0.852	-0.001	0.011	0.91
	BMI	rs4714290	T	C	0.715	0.010	0.009	0.23
	HbA1c	rs10305518	G	T	0.057	-0.034	0.017	0.04
Secondary analyses	HbA1c	rs10305492	G	A	0.984	0.001	0.031	0.98

BMI, body mass index; GLP-1R, glucagon-like peptide-1 receptor; HbA1c, glycated hemoglobin.

eTable 4. MR estimates for the associations of GLP-1R activation with HbA1c (mmol/mol) and BMI (kg/m^2).

Category	Exposure	Outcome	SNPs	Method	Beta (95% CI)	*P* value	*P* value (Q)
Primary analyses	Lower BMI via GLP-1R activation	HbA1c	2	IVW	-0.32 (-0.74 to 0.11)	0.14	0.10
	Lower HbA1c via GLP-1R activation	BMI	1	IVW	-0.04 (-0.22 to 0.14)	0.67	
Secondary analyses	Lower HbA1c via GLP-1R activation	BMI	1	IVW	-0.004 (-0.18 to 0.17)	0.97	

BMI, body mass index; GLP-1R, glucagon-like peptide-1 receptor; HbA1c, glycated hemoglobin; IVW, inverse variance weighted; MR, Mendelian randomization.

eTable 5. SNP-specific estimates for genetic instruments for BMI (kg/m^2) and HbA1c (mmol/mol).

Phenotype	Sex	SNP	Effect allele	Other allele	Effect allele frequency	Beta	SE	*P* value	F-statistic
BMI	Overall	rs2803316	A	G	0.455	-0.068	0.008	1.9E-16	69.8
BMI	Overall	rs7535528	A	G	0.372	-0.074	0.009	1.6E-17	73.2
BMI	Overall	rs6577584	T	G	0.659	-0.057	0.009	3.4E-11	43.0
BMI	Overall	rs2791653	A	G	0.240	0.064	0.009	1.0E-12	49.0
BMI	Overall	rs9435739	A	G	0.661	-0.052	0.009	9.9E-10	36.7
BMI	Overall	rs4655141	T	C	0.836	-0.083	0.011	6.2E-15	61.8
BMI	Overall	rs10798888	T	G	0.189	0.062	0.011	5.8E-09	34.9
BMI	Overall	rs12022461	A	G	0.167	-0.080	0.011	9.8E-14	57.6
BMI	Overall	rs7512146	T	G	0.527	-0.047	0.008	1.3E-08	32.6
BMI	Overall	rs11586036	A	C	0.935	-0.095	0.017	1.1E-08	32.0
BMI	Overall	rs11206436	T	C	0.893	0.071	0.013	4.7E-08	29.6
BMI	Overall	rs1707322	A	G	0.316	-0.065	0.009	1.6E-14	57.1
BMI	Overall	rs2984618	T	G	0.441	0.079	0.008	3.8E-24	106.3
BMI	Overall	rs657452	A	G	0.404	0.090	0.008	3.2E-30	138.1
BMI	Overall	rs17425707	T	C	0.900	-0.083	0.013	6.2E-10	38.2
BMI	Overall	rs2481665	T	C	0.582	0.077	0.008	3.2E-23	101.3
BMI	Overall	rs2503185	A	G	0.510	0.062	0.008	1.3E-14	58.5
BMI	Overall	rs3101336	T	C	0.383	-0.122	0.008	4.8E-54	252.0
BMI	Overall	rs12036473	A	T	0.618	-0.082	0.008	2.8E-24	101.2
BMI	Overall	rs12049202	T	C	0.188	0.115	0.010	3.3E-29	129.5
BMI	Overall	rs2166171	T	C	0.624	-0.050	0.009	5.5E-09	32.7
BMI	Overall	rs12034762	T	C	0.391	0.060	0.008	6.3E-13	54.1
BMI	Overall	rs11165643	T	C	0.580	0.089	0.008	4.5E-30	133.7
BMI	Overall	rs10747488	A	C	0.780	-0.064	0.010	1.5E-11	44.9
BMI	Overall	rs17024393	T	C	0.969	-0.310	0.024	7.1E-39	172.7
BMI	Overall	rs197374	T	C	0.403	0.068	0.008	3.2E-16	68.8
BMI	Overall	rs12120851	T	C	0.631	-0.056	0.010	2.9E-08	30.5
BMI	Overall	rs7534091	A	G	0.718	-0.058	0.009	6.7E-11	44.4
BMI	Overall	rs6587552	A	G	0.241	0.075	0.010	2.2E-15	61.6
BMI	Overall	rs3856261	A	G	0.498	0.056	0.008	1.1E-11	46.6
BMI	Overall	rs1750307	A	T	0.366	0.062	0.009	2.8E-13	51.4
BMI	Overall	rs10733051	A	G	0.512	0.045	0.008	7.0E-09	33.8
BMI	Overall	rs12564992	A	G	0.888	-0.091	0.012	2.6E-13	52.8
BMI	Overall	rs543874	A	G	0.779	-0.230	0.010	3.1E-125	573.6
BMI	Overall	rs10920678	A	G	0.425	0.072	0.008	7.1E-20	86.7
BMI	Overall	rs2400414	T	C	0.346	-0.061	0.009	5.5E-13	49.0
BMI	Overall	rs2820295	A	G	0.327	0.113	0.009	5.6E-39	170.4
BMI	Overall	rs10920336	A	G	0.533	-0.049	0.008	4.2E-09	35.3
BMI	Overall	rs1006317	T	G	0.127	0.077	0.012	3.7E-10	37.9
BMI	Overall	rs6661316	T	C	0.589	0.058	0.008	1.7E-13	56.3
BMI	Overall	rs10489615	A	G	0.400	0.048	0.008	1.3E-08	33.9
BMI	Overall	rs946824	T	C	0.136	0.095	0.012	4.8E-15	62.1
BMI	Overall	rs4577313	A	G	0.241	0.076	0.010	2.6E-15	62.4
BMI	Overall	rs13021737	A	G	0.157	-0.278	0.010	2.9E-161	757.6
BMI	Overall	rs10929925	A	C	0.411	-0.068	0.008	3.0E-18	78.8

BMI	Overall	rs11902450	T	C	0.105	0.080	0.013	1.1E-09	38.3
BMI	Overall	rs10182181	A	G	0.505	-0.157	0.008	2.5E-91	417.7
BMI	Overall	rs1260326	T	C	0.391	-0.051	0.008	1.2E-10	39.6
BMI	Overall	rs17327461	T	C	0.446	0.059	0.008	7.6E-14	58.1
BMI	Overall	rs10185199	A	G	0.286	-0.069	0.010	3.2E-13	51.1
BMI	Overall	rs10169594	T	C	0.648	-0.058	0.009	1.3E-11	44.4
BMI	Overall	rs7561278	T	C	0.767	0.081	0.010	4.9E-16	64.8
BMI	Overall	rs930295	A	C	0.155	0.100	0.011	2.0E-19	81.8
BMI	Overall	rs805412	A	G	0.434	-0.047	0.008	1.1E-08	33.2
BMI	Overall	rs4671328	T	G	0.450	0.103	0.008	3.4E-36	158.5
BMI	Overall	rs6545714	A	G	0.611	-0.093	0.008	4.0E-32	147.0
BMI	Overall	rs2861683	A	C	0.597	0.068	0.008	1.9E-16	68.8
BMI	Overall	rs17020497	A	G	0.134	0.067	0.012	2.0E-08	31.4
BMI	Overall	rs12714199	T	C	0.615	-0.068	0.008	3.2E-16	68.8
BMI	Overall	rs4303732	T	C	0.614	0.081	0.008	1.3E-22	98.8
BMI	Overall	rs264941	A	C	0.468	-0.060	0.008	1.8E-13	53.2
BMI	Overall	rs10197031	T	C	0.726	-0.077	0.009	5.0E-18	71.8
BMI	Overall	rs11884795	A	G	0.268	0.054	0.009	1.6E-09	38.7
BMI	Overall	rs4988235	A	G	0.721	0.060	0.008	7.1E-13	53.2
BMI	Overall	rs17814208	A	G	0.758	-0.062	0.010	7.2E-11	41.6
BMI	Overall	rs16824165	A	G	0.116	0.077	0.013	2.2E-09	35.1
BMI	Overall	rs429343	A	G	0.434	0.070	0.008	8.4E-18	73.8
BMI	Overall	rs2119753	A	G	0.623	0.048	0.008	1.0E-08	34.6
BMI	Overall	rs3764835	A	G	0.151	-0.062	0.012	3.8E-08	29.3
BMI	Overall	rs12692596	T	C	0.362	0.058	0.008	1.0E-12	49.8
BMI	Overall	rs1521527	C	G	0.518	-0.049	0.008	1.8E-09	36.0
BMI	Overall	rs3754963	A	T	0.763	0.062	0.009	2.4E-11	46.1
BMI	Overall	rs2138348	T	G	0.284	-0.064	0.009	1.4E-12	49.7
BMI	Overall	rs2119137	A	G	0.662	-0.063	0.009	2.3E-13	53.0
BMI	Overall	rs7588437	A	G	0.366	-0.079	0.008	2.3E-22	94.2
BMI	Overall	rs6757852	A	G	0.499	0.051	0.008	3.8E-10	38.9
BMI	Overall	rs7593917	A	G	0.454	-0.055	0.008	9.7E-13	51.7
BMI	Overall	rs4482463	A	C	0.917	-0.149	0.015	4.8E-23	100.0
BMI	Overall	rs6715020	T	C	0.381	-0.050	0.008	1.3E-09	38.1
BMI	Overall	rs11692326	T	C	0.230	0.071	0.009	1.7E-14	59.9
BMI	Overall	rs7599312	A	G	0.274	-0.087	0.009	1.5E-23	102.2
BMI	Overall	rs12987009	A	T	0.585	-0.052	0.008	1.8E-10	41.1
BMI	Overall	rs6725931	T	C	0.848	0.090	0.012	2.3E-15	60.7
BMI	Overall	rs4973618	A	G	0.662	-0.071	0.009	1.3E-16	67.6
BMI	Overall	rs6720868	T	C	0.308	0.074	0.009	1.8E-17	73.2
BMI	Overall	rs7568228	C	G	0.540	-0.050	0.008	1.0E-09	37.4
BMI	Overall	rs17535749	A	G	0.108	0.076	0.013	3.5E-09	34.7
BMI	Overall	rs10510419	T	G	0.147	-0.081	0.011	2.2E-13	53.4
BMI	Overall	rs2600226	T	C	0.671	-0.056	0.009	1.4E-10	41.5
BMI	Overall	rs1048637	T	G	0.547	-0.045	0.008	2.9E-08	30.6
BMI	Overall	rs4858193	T	C	0.720	0.064	0.009	2.4E-12	49.0
BMI	Overall	rs6804842	A	G	0.430	-0.068	0.008	7.6E-18	77.7
BMI	Overall	rs11921432	T	C	0.891	-0.091	0.013	5.4E-12	49.0

BMI	Overall	rs1799923	A	G	0.112	-0.108	0.012	1.1E-17	74.2
BMI	Overall	rs28350	A	G	0.172	0.083	0.011	1.1E-14	61.1
BMI	Overall	rs4017425	T	C	0.468	-0.057	0.008	2.9E-12	48.2
BMI	Overall	rs11713193	A	G	0.516	0.118	0.008	3.0E-48	209.4
BMI	Overall	rs2365389	T	C	0.400	-0.081	0.008	6.5E-25	110.3
BMI	Overall	rs925018	C	G	0.667	-0.062	0.008	4.3E-14	58.5
BMI	Overall	rs11915371	A	C	0.802	-0.074	0.010	2.3E-13	53.8
BMI	Overall	rs12636480	T	G	0.351	0.062	0.009	7.0E-13	50.6
BMI	Overall	rs9827823	T	C	0.855	0.087	0.011	3.4E-15	62.6
BMI	Overall	rs9818122	T	C	0.793	-0.110	0.010	4.0E-30	130.0
BMI	Overall	rs11128021	A	G	0.162	-0.087	0.012	1.6E-14	56.9
BMI	Overall	rs1492014	T	C	0.567	-0.082	0.008	1.4E-23	101.2
BMI	Overall	rs1436344	C	G	0.570	0.071	0.008	1.1E-17	74.8
BMI	Overall	rs7640424	T	C	0.311	-0.065	0.009	1.2E-14	56.3
BMI	Overall	rs17681451	A	G	0.076	-0.108	0.015	7.4E-13	52.7
BMI	Overall	rs6808814	T	C	0.734	0.057	0.009	1.3E-09	38.6
BMI	Overall	rs7631156	A	G	0.309	0.103	0.009	3.3E-32	142.7
BMI	Overall	rs687339	T	C	0.770	0.090	0.009	4.3E-22	97.9
BMI	Overall	rs16851483	T	G	0.072	0.169	0.016	4.9E-25	107.2
BMI	Overall	rs355777	C	G	0.398	0.073	0.008	2.1E-18	78.9
BMI	Overall	rs9826775	A	G	0.851	0.075	0.012	6.6E-11	41.7
BMI	Overall	rs2047648	A	T	0.747	-0.064	0.010	7.9E-12	44.2
BMI	Overall	rs3732927	T	C	0.291	0.048	0.008	1.1E-08	33.9
BMI	Overall	rs12635553	A	T	0.486	0.048	0.008	4.1E-09	33.9
BMI	Overall	rs39654	A	G	0.451	-0.078	0.008	1.7E-21	91.9
BMI	Overall	rs6443750	T	C	0.199	-0.073	0.010	7.3E-13	52.4
BMI	Overall	rs865809	A	G	0.225	0.060	0.010	6.5E-10	38.4
BMI	Overall	rs9816226	A	T	0.176	-0.151	0.010	1.4E-50	225.0
BMI	Overall	rs7616009	A	G	0.160	-0.075	0.012	4.3E-11	42.8
BMI	Overall	rs2051559	T	C	0.859	-0.080	0.012	3.8E-11	44.6
BMI	Overall	rs6818414	T	C	0.516	-0.048	0.008	4.6E-09	34.6
BMI	Overall	rs1477890	A	G	0.488	-0.062	0.008	6.1E-14	56.7
BMI	Overall	rs9291467	T	C	0.465	0.068	0.008	4.6E-17	69.8
BMI	Overall	rs6448587	A	C	0.806	0.075	0.011	1.6E-12	50.3
BMI	Overall	rs337637	A	G	0.359	-0.066	0.008	6.2E-16	64.9
BMI	Overall	rs10938397	A	G	0.570	-0.155	0.008	2.4E-86	405.0
BMI	Overall	rs2271046	A	T	0.695	-0.055	0.009	3.0E-10	40.8
BMI	Overall	rs1492767	T	C	0.468	0.046	0.008	3.5E-09	35.3
BMI	Overall	rs2192158	A	G	0.449	0.066	0.008	7.4E-16	64.9
BMI	Overall	rs1346841	A	G	0.412	-0.061	0.008	3.2E-13	54.9
BMI	Overall	rs10002111	A	G	0.223	0.060	0.010	1.3E-09	35.4
BMI	Overall	rs10033843	A	G	0.215	0.067	0.010	1.1E-11	44.4
BMI	Overall	rs17507682	T	G	0.154	0.065	0.012	1.9E-08	31.6
BMI	Overall	rs4148155	A	G	0.887	0.092	0.012	1.3E-13	54.5
BMI	Overall	rs4286488	A	G	0.761	0.058	0.010	1.7E-09	36.6
BMI	Overall	rs7678054	A	G	0.479	-0.048	0.008	4.6E-09	33.9
BMI	Overall	rs3796432	T	G	0.367	-0.054	0.009	2.2E-10	39.4
BMI	Overall	rs13107325	T	C	0.082	0.225	0.015	3.8E-47	213.9

BMI	Overall	rs326893	T	C	0.581	0.058	0.008	1.9E-12	50.7
BMI	Overall	rs7696649	A	G	0.278	0.056	0.009	4.7E-10	37.3
BMI	Overall	rs4864201	T	C	0.347	0.066	0.008	4.3E-16	64.9
BMI	Overall	rs1296328	A	C	0.446	0.080	0.008	3.5E-22	95.3
BMI	Overall	rs17367750	T	C	0.312	-0.059	0.009	2.0E-11	45.9
BMI	Overall	rs9992189	C	G	0.608	-0.047	0.008	2.0E-08	32.6
BMI	Overall	rs3914628	T	C	0.859	0.079	0.011	6.9E-13	51.5
BMI	Overall	rs750090	T	C	0.626	0.054	0.009	2.5E-10	39.4
BMI	Overall	rs13110266	A	G	0.404	-0.060	0.008	4.0E-14	60.1
BMI	Overall	rs7685628	A	T	0.401	0.049	0.008	6.1E-09	35.3
BMI	Overall	rs1522569	T	G	0.818	0.068	0.011	1.6E-10	41.1
BMI	Overall	rs1437842	A	G	0.491	-0.051	0.008	8.5E-10	38.9
BMI	Overall	rs4565118	A	C	0.502	-0.051	0.009	7.3E-09	34.7
BMI	Overall	rs698147	A	G	0.447	0.056	0.008	9.7E-12	46.6
BMI	Overall	rs6890310	A	G	0.292	-0.057	0.009	3.3E-10	39.2
BMI	Overall	rs7730004	T	C	0.661	0.067	0.009	1.5E-14	59.6
BMI	Overall	rs7704281	A	G	0.046	0.119	0.020	1.2E-09	36.6
BMI	Overall	rs13186194	T	C	0.617	0.048	0.008	2.2E-09	34.6
BMI	Overall	rs4700608	T	C	0.524	-0.075	0.008	4.3E-20	83.1
BMI	Overall	rs6886740	T	C	0.662	0.055	0.009	3.1E-10	40.1
BMI	Overall	rs1159692	A	C	0.475	0.065	0.008	5.5E-15	63.1
BMI	Overall	rs2112347	T	G	0.630	0.133	0.008	1.2E-61	263.6
BMI	Overall	rs10942267	A	G	0.693	0.071	0.009	6.5E-16	67.6
BMI	Overall	rs2962334	T	G	0.027	0.190	0.028	1.8E-11	45.0
BMI	Overall	rs1501673	A	G	0.136	0.139	0.012	2.7E-31	133.6
BMI	Overall	rs12652212	A	G	0.561	-0.063	0.008	1.6E-15	67.0
BMI	Overall	rs7713317	A	G	0.720	-0.080	0.009	2.0E-20	85.0
BMI	Overall	rs6882366	T	C	0.396	-0.063	0.008	4.4E-14	59.4
BMI	Overall	rs11739877	T	C	0.623	0.056	0.009	4.0E-11	41.5
BMI	Overall	rs6888194	T	C	0.844	-0.061	0.011	3.6E-08	30.5
BMI	Overall	rs40067	A	G	0.170	-0.121	0.011	9.7E-29	120.0
BMI	Overall	rs459552	A	T	0.775	-0.064	0.009	8.4E-12	49.0
BMI	Overall	rs6893539	A	C	0.698	-0.059	0.009	6.2E-11	41.2
BMI	Overall	rs6864049	A	G	0.489	-0.058	0.008	1.5E-13	57.2
BMI	Overall	rs6886072	T	C	0.467	-0.048	0.008	4.1E-09	34.6
BMI	Overall	rs13174863	A	G	0.854	-0.095	0.011	1.9E-17	73.4
BMI	Overall	rs17405603	A	T	0.706	-0.060	0.009	4.4E-11	43.3
BMI	Overall	rs2973157	A	G	0.671	-0.054	0.010	4.2E-08	28.4
BMI	Overall	rs7715256	T	G	0.564	-0.076	0.008	4.0E-22	97.5
BMI	Overall	rs7734385	A	G	0.445	-0.049	0.008	6.1E-10	39.8
BMI	Overall	rs2861089	A	T	0.381	0.050	0.008	1.5E-09	38.1
BMI	Overall	rs7727781	T	C	0.516	0.045	0.008	4.3E-08	29.9
BMI	Overall	rs2053682	A	C	0.678	0.082	0.009	2.6E-20	89.2
BMI	Overall	rs6556301	T	G	0.371	-0.054	0.008	8.1E-11	44.2
BMI	Overall	rs2228213	A	G	0.337	-0.069	0.008	5.5E-17	71.8
BMI	Overall	rs11757278	T	C	0.696	0.064	0.009	6.9E-13	49.0
BMI	Overall	rs3806114	A	G	0.692	-0.058	0.009	1.5E-11	44.4
BMI	Overall	rs2066295	A	G	0.758	0.068	0.010	2.3E-12	50.4

BMI	Overall	rs3115667	T	C	0.282	-0.086	0.009	5.9E-21	88.8
BMI	Overall	rs2281819	A	T	0.229	-0.074	0.010	2.1E-14	59.3
BMI	Overall	rs2744974	T	C	0.324	0.125	0.008	1.3E-51	235.7
BMI	Overall	rs2436728	A	G	0.407	0.091	0.008	2.0E-29	123.6
BMI	Overall	rs1358980	T	C	0.474	-0.062	0.008	5.2E-15	57.6
BMI	Overall	rs2206277	T	C	0.156	0.196	0.010	1.8E-83	377.5
BMI	Overall	rs1327259	A	G	0.610	0.075	0.008	1.5E-19	85.3
BMI	Overall	rs6915002	T	C	0.411	0.048	0.008	8.7E-09	33.9
BMI	Overall	rs9370410	A	G	0.729	0.050	0.009	2.6E-08	30.5
BMI	Overall	rs2622274	T	G	0.455	-0.051	0.008	3.2E-10	39.6
BMI	Overall	rs13213867	A	G	0.803	0.059	0.011	1.0E-08	31.3
BMI	Overall	rs6921533	T	C	0.293	0.050	0.009	1.4E-08	33.4
BMI	Overall	rs9294260	A	G	0.469	0.067	0.008	8.2E-18	76.6
BMI	Overall	rs6909685	T	C	0.330	-0.072	0.009	2.8E-16	68.5
BMI	Overall	rs9320823	T	C	0.412	-0.079	0.008	2.1E-21	94.2
BMI	Overall	rs12530388	A	C	0.474	0.048	0.008	4.2E-09	34.6
BMI	Overall	rs156201	C	G	0.746	0.060	0.009	1.6E-10	42.6
BMI	Overall	rs768023	A	G	0.603	0.077	0.008	1.1E-22	101.3
BMI	Overall	rs2357760	A	G	0.674	0.069	0.008	2.1E-16	70.8
BMI	Overall	rs2875762	C	G	0.252	0.062	0.010	1.1E-10	41.6
BMI	Overall	rs1268065	A	G	0.500	-0.048	0.008	7.1E-10	39.1
BMI	Overall	rs9375702	T	C	0.679	-0.051	0.009	5.5E-09	34.7
BMI	Overall	rs2246012	T	C	0.851	-0.077	0.011	1.2E-13	53.6
BMI	Overall	rs6922607	A	G	0.808	-0.062	0.011	1.9E-09	34.9
BMI	Overall	rs765875	T	C	0.469	-0.063	0.008	1.1E-14	60.3
BMI	Overall	rs7760482	A	G	0.616	-0.049	0.009	6.2E-09	32.1
BMI	Overall	rs12527426	A	G	0.300	0.072	0.009	5.5E-16	62.3
BMI	Overall	rs9478496	T	C	0.845	-0.075	0.011	8.2E-12	46.6
BMI	Overall	rs13191362	A	G	0.857	0.113	0.012	4.1E-21	88.4
BMI	Overall	rs9458814	T	C	0.771	-0.054	0.010	2.3E-08	31.4
BMI	Overall	rs6461115	A	G	0.762	0.064	0.009	1.5E-12	49.7
BMI	Overall	rs6463489	T	C	0.096	0.080	0.012	2.5E-10	41.3
BMI	Overall	rs7779296	A	G	0.716	-0.050	0.009	4.6E-09	32.7
BMI	Overall	rs4307239	A	G	0.536	-0.055	0.008	1.5E-11	45.8
BMI	Overall	rs213518	T	C	0.850	-0.074	0.012	2.5E-10	40.6
BMI	Overall	rs215669	A	G	0.602	-0.072	0.008	8.9E-18	76.8
BMI	Overall	rs2108719	A	G	0.727	0.051	0.009	2.3E-08	31.7
BMI	Overall	rs217433	T	C	0.803	-0.055	0.010	3.0E-08	30.0
BMI	Overall	rs2289379	T	C	0.392	-0.066	0.009	7.0E-15	57.9
BMI	Overall	rs10499694	A	G	0.494	0.062	0.008	1.3E-15	66.0
BMI	Overall	rs11772246	T	C	0.823	0.070	0.011	3.7E-11	43.4
BMI	Overall	rs17207196	T	C	0.420	-0.106	0.008	1.6E-36	167.5
BMI	Overall	rs17149254	T	C	0.205	0.114	0.011	3.0E-25	107.1
BMI	Overall	rs6973656	A	G	0.584	-0.049	0.008	6.3E-09	35.3
BMI	Overall	rs1965529	A	G	0.773	0.077	0.011	7.7E-14	53.6
BMI	Overall	rs274628	A	C	0.337	-0.049	0.009	1.4E-08	32.1
BMI	Overall	rs2283006	A	G	0.488	0.063	0.008	8.1E-15	60.3
BMI	Overall	rs13240600	A	G	0.834	0.087	0.011	7.9E-16	66.9

BMI	Overall	rs1721447	T	G	0.510	-0.048	0.008	3.8E-09	34.6
BMI	Overall	rs2396625	A	T	0.418	-0.085	0.008	2.8E-24	107.2
BMI	Overall	rs13245051	A	G	0.456	0.072	0.008	1.1E-18	77.9
BMI	Overall	rs10261050	T	C	0.475	0.054	0.008	4.5E-11	44.2
BMI	Overall	rs2283093	T	C	0.195	0.058	0.010	1.1E-08	33.2
BMI	Overall	rs7802342	T	G	0.705	-0.060	0.009	6.2E-11	42.6
BMI	Overall	rs17160760	A	T	0.105	-0.079	0.013	1.3E-09	37.3
BMI	Overall	rs11773362	T	C	0.340	-0.050	0.009	6.4E-09	34.0
BMI	Overall	rs2907948	A	G	0.244	-0.070	0.009	1.9E-14	58.2
BMI	Overall	rs1700082	C	G	0.659	0.045	0.008	3.4E-08	30.6
BMI	Overall	rs4240673	T	C	0.455	0.084	0.008	1.6E-26	119.6
BMI	Overall	rs13263601	A	C	0.659	-0.070	0.009	4.9E-16	64.9
BMI	Overall	rs10110189	T	C	0.106	-0.075	0.013	2.2E-08	31.0
BMI	Overall	rs10101364	T	C	0.680	0.058	0.009	5.6E-11	44.4
BMI	Overall	rs1421334	A	C	0.455	0.065	0.008	3.1E-15	63.1
BMI	Overall	rs2466103	T	G	0.693	-0.058	0.009	8.0E-12	45.2
BMI	Overall	rs6468266	A	T	0.418	-0.054	0.008	7.7E-11	44.2
BMI	Overall	rs12681792	A	C	0.202	0.072	0.010	2.9E-12	51.0
BMI	Overall	rs12334877	A	G	0.191	-0.069	0.010	2.2E-11	47.0
BMI	Overall	rs1431659	A	G	0.274	0.092	0.009	2.3E-23	101.1
BMI	Overall	rs17405819	T	C	0.685	0.101	0.009	6.0E-33	137.4
BMI	Overall	rs2196618	A	G	0.263	-0.066	0.009	1.5E-12	52.0
BMI	Overall	rs2120710	A	G	0.653	0.049	0.009	2.0E-08	31.5
BMI	Overall	rs12680842	A	G	0.684	0.068	0.008	3.4E-16	69.8
BMI	Overall	rs6469351	T	C	0.672	0.053	0.009	3.7E-10	38.0
BMI	Overall	rs3808477	T	C	0.274	-0.087	0.009	8.7E-22	91.8
BMI	Overall	rs11781699	T	C	0.813	-0.068	0.010	1.6E-11	45.1
BMI	Overall	rs6470144	T	G	0.650	0.047	0.009	3.1E-08	29.6
BMI	Overall	rs7842934	T	C	0.921	-0.086	0.015	1.5E-08	33.0
BMI	Overall	rs305256	T	C	0.226	-0.055	0.010	2.8E-08	30.0
BMI	Overall	rs16906838	T	C	0.049	-0.119	0.019	4.3E-10	38.1
BMI	Overall	rs11782074	T	G	0.368	0.060	0.009	4.2E-12	47.5
BMI	Overall	rs10099330	A	G	0.538	-0.057	0.008	3.2E-12	49.0
BMI	Overall	rs10975933	C	G	0.663	0.055	0.009	2.1E-10	40.1
BMI	Overall	rs1948080	T	G	0.630	0.065	0.009	1.1E-14	57.1
BMI	Overall	rs10961649	T	C	0.324	0.050	0.009	1.5E-08	33.4
BMI	Overall	rs4740619	T	C	0.544	0.091	0.008	3.1E-31	139.5
BMI	Overall	rs10962550	C	G	0.176	0.089	0.011	5.7E-17	71.5
BMI	Overall	rs10811868	A	G	0.320	-0.048	0.009	3.2E-08	30.9
BMI	Overall	rs10968114	A	C	0.533	0.054	0.008	3.3E-11	44.2
BMI	Overall	rs1412235	C	G	0.315	0.114	0.008	2.3E-42	194.4
BMI	Overall	rs10121187	C	G	0.509	0.049	0.008	2.0E-09	36.0
BMI	Overall	rs2275003	A	G	0.501	0.053	0.008	9.4E-12	48.1
BMI	Overall	rs13296413	T	C	0.381	-0.071	0.008	2.5E-17	75.8
BMI	Overall	rs2134858	T	C	0.512	-0.056	0.008	5.9E-12	47.4
BMI	Overall	rs7861160	T	C	0.594	0.046	0.008	3.7E-08	31.2
BMI	Overall	rs1999433	T	C	0.440	-0.051	0.008	2.9E-10	39.6
BMI	Overall	rs2777768	A	G	0.723	0.057	0.009	6.4E-10	39.2

BMI	Overall	rs7357754	A	G	0.499	-0.058	0.008	1.8E-12	49.8
BMI	Overall	rs10992867	A	G	0.269	0.078	0.009	3.2E-17	72.7
BMI	Overall	rs7025938	C	G	0.680	-0.078	0.009	1.5E-19	81.0
BMI	Overall	rs7024334	T	G	0.225	0.065	0.010	4.7E-12	45.6
BMI	Overall	rs1928295	T	C	0.571	0.064	0.008	2.2E-16	70.1
BMI	Overall	rs1877875	T	C	0.434	-0.052	0.008	2.5E-10	41.1
BMI	Overall	rs10733682	A	G	0.459	0.071	0.008	1.8E-19	85.6
BMI	Overall	rs2267958	A	G	0.509	-0.062	0.009	2.0E-13	52.2
BMI	Overall	rs4740383	A	G	0.419	0.063	0.009	7.0E-14	53.0
BMI	Overall	rs10858334	C	G	0.848	-0.071	0.012	4.9E-09	35.0
BMI	Overall	rs7907470	A	G	0.916	-0.085	0.015	7.4E-09	32.6
BMI	Overall	rs7893571	T	G	0.674	0.060	0.009	5.8E-12	48.2
BMI	Overall	rs7084454	A	G	0.309	0.095	0.009	4.5E-27	121.0
BMI	Overall	rs10829164	T	C	0.147	0.073	0.012	2.4E-10	39.6
BMI	Overall	rs4097319	T	G	0.564	0.051	0.008	4.3E-10	39.6
BMI	Overall	rs12765914	T	C	0.080	0.109	0.015	2.0E-13	53.1
BMI	Overall	rs1624134	C	G	0.393	0.048	0.008	6.5E-09	34.6
BMI	Overall	rs12259464	A	G	0.481	0.052	0.008	1.5E-10	41.1
BMI	Overall	rs10761785	T	G	0.514	-0.064	0.008	3.5E-16	69.1
BMI	Overall	rs12098284	T	C	0.121	0.088	0.012	9.9E-13	50.1
BMI	Overall	rs11001259	A	T	0.177	-0.066	0.012	1.2E-08	33.1
BMI	Overall	rs7899106	A	G	0.950	-0.157	0.018	1.7E-18	78.1
BMI	Overall	rs10788494	C	G	0.484	0.063	0.008	5.5E-15	60.3
BMI	Overall	rs2439823	A	G	0.451	-0.079	0.008	6.5E-22	94.2
BMI	Overall	rs17094222	T	C	0.791	-0.083	0.010	4.0E-18	74.8
BMI	Overall	rs1886276	A	G	0.571	-0.052	0.008	3.0E-10	40.4
BMI	Overall	rs7903146	T	C	0.279	-0.086	0.009	1.7E-23	97.8
BMI	Overall	rs2257791	A	G	0.756	-0.067	0.010	1.6E-12	48.3
BMI	Overall	rs845084	A	G	0.267	0.065	0.009	3.2E-12	51.2
BMI	Overall	rs17636031	T	C	0.724	-0.074	0.009	3.9E-17	73.2
BMI	Overall	rs4880341	T	C	0.573	-0.062	0.008	3.1E-14	58.5
BMI	Overall	rs12416812	A	G	0.507	0.051	0.008	5.2E-11	43.9
BMI	Overall	rs4256980	C	G	0.331	-0.090	0.008	8.6E-29	121.0
BMI	Overall	rs900144	T	C	0.578	0.071	0.008	1.5E-18	75.8
BMI	Overall	rs2074314	T	C	0.636	0.050	0.008	1.4E-09	38.1
BMI	Overall	rs6265	T	C	0.184	-0.199	0.010	7.4E-89	386.8
BMI	Overall	rs570463	A	C	0.327	-0.058	0.009	4.3E-11	45.2
BMI	Overall	rs11030618	T	C	0.579	0.053	0.008	1.7E-10	41.9
BMI	Overall	rs2065418	T	G	0.646	0.067	0.009	6.1E-15	59.6
BMI	Overall	rs2862996	T	G	0.701	-0.104	0.008	3.6E-35	161.4
BMI	Overall	rs10838202	C	G	0.556	0.054	0.008	5.5E-11	44.2
BMI	Overall	rs10742752	T	C	0.376	-0.059	0.008	1.2E-13	52.3
BMI	Overall	rs7124681	A	C	0.416	0.124	0.008	4.0E-55	258.0
BMI	Overall	rs6591407	A	C	0.198	-0.060	0.010	3.6E-09	34.9
BMI	Overall	rs562664	T	C	0.187	-0.066	0.011	4.2E-10	39.3
BMI	Overall	rs7102454	T	C	0.644	-0.081	0.009	3.8E-21	87.1
BMI	Overall	rs592483	T	C	0.593	-0.066	0.008	1.5E-16	64.9
BMI	Overall	rs737185	A	G	0.781	0.066	0.010	4.2E-11	42.6

BMI	Overall	rs349088	A	C	0.478	-0.062	0.008	3.5E-14	58.5
BMI	Overall	rs10741329	A	G	0.694	0.055	0.009	3.9E-10	40.8
BMI	Overall	rs3019466	T	C	0.168	-0.062	0.012	4.9E-08	28.4
BMI	Overall	rs2605603	A	G	0.481	-0.050	0.008	2.0E-10	41.4
BMI	Overall	rs4937870	A	G	0.694	0.054	0.009	1.6E-09	34.7
BMI	Overall	rs10891549	T	C	0.465	0.046	0.008	1.9E-08	31.2
BMI	Overall	rs12286929	A	G	0.501	-0.085	0.008	1.9E-27	122.4
BMI	Overall	rs3825061	T	C	0.386	0.067	0.008	6.1E-16	67.8
BMI	Overall	rs6589936	A	C	0.387	0.054	0.009	2.2E-10	38.7
BMI	Overall	rs7944782	T	G	0.502	-0.069	0.008	3.6E-17	71.8
BMI	Overall	rs2007518	A	G	0.562	-0.062	0.008	2.0E-14	58.5
BMI	Overall	rs1941213	A	C	0.711	0.052	0.009	1.6E-08	32.3
BMI	Overall	rs329651	T	G	0.806	0.077	0.010	2.1E-14	58.0
BMI	Overall	rs12364470	T	G	0.854	-0.090	0.011	2.2E-17	72.3
BMI	Overall	rs11611246	T	G	0.199	0.107	0.010	2.0E-28	124.3
BMI	Overall	rs765125	T	C	0.586	-0.047	0.008	1.3E-08	33.2
BMI	Overall	rs10772983	T	C	0.539	-0.048	0.008	6.2E-10	39.1
BMI	Overall	rs11044430	A	T	0.844	0.077	0.011	3.8E-12	49.0
BMI	Overall	rs11046972	T	C	0.071	0.085	0.015	3.7E-08	30.3
BMI	Overall	rs10842240	C	G	0.126	0.094	0.012	1.2E-14	60.8
BMI	Overall	rs11170468	A	C	0.783	0.062	0.009	1.1E-11	46.8
BMI	Overall	rs1350430	T	C	0.533	-0.060	0.008	2.1E-13	54.1
BMI	Overall	rs7138803	A	G	0.387	0.143	0.008	3.1E-71	305.2
BMI	Overall	rs2271189	A	G	0.396	-0.068	0.009	9.3E-16	61.4
BMI	Overall	rs7975187	A	G	0.771	-0.066	0.010	3.9E-11	42.6
BMI	Overall	rs650198	T	C	0.729	-0.066	0.009	6.4E-13	52.0
BMI	Overall	rs11115176	T	C	0.784	0.063	0.009	6.8E-12	47.5
BMI	Overall	rs10506971	A	G	0.552	-0.068	0.008	4.7E-17	69.8
BMI	Overall	rs11105839	A	T	0.373	-0.054	0.008	1.2E-11	44.2
BMI	Overall	rs2712665	T	C	0.697	-0.052	0.009	5.4E-09	32.3
BMI	Overall	rs6539064	C	G	0.747	0.093	0.009	1.1E-23	104.3
BMI	Overall	rs1860561	A	G	0.209	0.076	0.009	1.7E-16	70.0
BMI	Overall	rs11066188	A	G	0.376	-0.055	0.008	3.1E-12	50.8
BMI	Overall	rs2707183	T	G	0.532	-0.045	0.008	4.9E-08	29.9
BMI	Overall	rs11615578	T	C	0.258	0.056	0.010	3.0E-09	34.2
BMI	Overall	rs12369179	T	C	0.085	-0.163	0.015	2.3E-28	120.3
BMI	Overall	rs7306544	T	C	0.881	-0.068	0.012	4.0E-08	29.8
BMI	Overall	rs11614340	T	C	0.707	-0.056	0.009	1.9E-10	42.3
BMI	Overall	rs9512648	A	G	0.474	0.046	0.008	4.1E-08	31.2
BMI	Overall	rs7323	C	G	0.268	-0.080	0.009	2.2E-18	76.3
BMI	Overall	rs4771218	A	G	0.624	-0.068	0.009	3.2E-15	61.4
BMI	Overall	rs9595908	T	C	0.643	0.074	0.008	3.7E-20	82.1
BMI	Overall	rs9603697	T	C	0.318	0.064	0.009	1.7E-13	55.4
BMI	Overall	rs12429545	A	G	0.122	0.150	0.012	1.4E-37	170.1
BMI	Overall	rs962796	T	C	0.194	0.067	0.010	1.9E-11	44.4
BMI	Overall	rs9569777	T	G	0.187	-0.097	0.010	3.4E-21	91.6
BMI	Overall	rs9527895	T	C	0.819	-0.076	0.011	3.7E-12	47.2
BMI	Overall	rs8181823	A	C	0.234	-0.060	0.010	4.4E-10	39.1

BMI	Overall	rs9599161	T	C	0.570	0.047	0.008	2.3E-09	37.5
BMI	Overall	rs1441264	A	G	0.585	0.084	0.008	7.6E-25	104.8
BMI	Overall	rs9602902	T	C	0.379	0.064	0.008	1.6E-14	62.1
BMI	Overall	rs1927790	T	C	0.606	-0.067	0.008	1.6E-17	76.6
BMI	Overall	rs9168	A	C	0.275	-0.067	0.009	1.1E-13	53.5
BMI	Overall	rs9559022	A	G	0.816	-0.058	0.011	4.9E-08	30.3
BMI	Overall	rs1536053	T	C	0.310	-0.059	0.009	8.0E-11	41.2
BMI	Overall	rs12868881	A	T	0.407	0.066	0.008	1.4E-15	65.9
BMI	Overall	rs10132280	A	C	0.311	-0.103	0.009	2.3E-33	141.3
BMI	Overall	rs4981693	A	G	0.774	0.097	0.010	7.9E-24	102.0
BMI	Overall	rs225882	T	C	0.751	0.054	0.009	8.6E-10	39.4
BMI	Overall	rs1958898	C	G	0.210	-0.070	0.010	4.2E-12	48.3
BMI	Overall	rs4900714	T	G	0.478	0.072	0.008	1.0E-18	77.9
BMI	Overall	rs217671	A	G	0.726	-0.072	0.009	4.1E-15	62.3
BMI	Overall	rs4430672	T	C	0.196	0.060	0.010	5.0E-09	34.9
BMI	Overall	rs3902951	T	G	0.769	-0.064	0.009	2.9E-12	49.7
BMI	Overall	rs17182027	A	G	0.565	-0.053	0.008	1.5E-10	41.9
BMI	Overall	rs7144011	T	G	0.233	0.126	0.010	2.4E-40	172.9
BMI	Overall	rs4517716	C	G	0.792	-0.056	0.010	4.3E-09	34.2
BMI	Overall	rs12888545	A	G	0.746	-0.064	0.010	1.8E-11	44.2
BMI	Overall	rs1951455	T	C	0.281	-0.071	0.009	6.0E-15	60.7
BMI	Overall	rs9989141	T	C	0.618	0.081	0.008	8.2E-23	97.7
BMI	Overall	rs8008285	T	C	0.849	-0.065	0.012	1.7E-08	31.6
BMI	Overall	rs7161194	A	G	0.344	0.091	0.009	2.2E-24	100.0
BMI	Overall	rs4906263	C	G	0.656	-0.085	0.009	8.1E-23	95.6
BMI	Overall	rs2273175	T	C	0.679	-0.058	0.009	3.7E-11	45.2
BMI	Overall	rs12594043	C	G	0.521	0.049	0.008	1.3E-09	36.0
BMI	Overall	rs7172627	A	G	0.523	-0.055	0.008	1.8E-11	45.0
BMI	Overall	rs11636611	T	C	0.498	0.050	0.008	8.9E-10	37.4
BMI	Overall	rs9944219	A	G	0.604	-0.057	0.008	8.1E-13	49.0
BMI	Overall	rs12912198	T	C	0.273	-0.048	0.009	3.4E-08	30.9
BMI	Overall	rs6493498	T	C	0.447	0.066	0.008	4.8E-17	73.3
BMI	Overall	rs10518694	A	C	0.139	0.069	0.012	3.4E-09	36.0
BMI	Overall	rs12439632	C	G	0.168	0.070	0.011	3.7E-10	39.7
BMI	Overall	rs339991	A	G	0.420	-0.060	0.008	3.5E-13	54.1
BMI	Overall	rs1559673	A	C	0.969	0.173	0.024	2.2E-13	53.7
BMI	Overall	rs2241423	A	G	0.229	-0.143	0.009	3.6E-54	246.0
BMI	Overall	rs7171864	A	G	0.694	0.063	0.008	2.6E-14	60.3
BMI	Overall	rs2470893	T	C	0.305	0.051	0.008	9.4E-10	39.6
BMI	Overall	rs12914623	C	G	0.272	-0.076	0.009	2.0E-16	70.0
BMI	Overall	rs16946314	A	G	0.212	-0.066	0.010	6.9E-11	42.6
BMI	Overall	rs11633626	A	C	0.631	-0.075	0.009	7.3E-19	76.1
BMI	Overall	rs6496248	A	T	0.635	0.050	0.009	2.9E-09	33.4
BMI	Overall	rs2715423	A	G	0.281	-0.055	0.009	1.9E-09	36.6
BMI	Overall	rs214249	T	G	0.607	0.066	0.008	2.7E-15	65.9
BMI	Overall	rs12448257	A	G	0.219	0.077	0.010	9.0E-16	64.8
BMI	Overall	rs879620	T	C	0.597	0.109	0.008	8.4E-39	176.7
BMI	Overall	rs2058527	T	G	0.272	-0.055	0.009	1.8E-09	36.6

BMI	Overall	rs9926784	T	C	0.801	0.114	0.010	1.1E-30	127.4
BMI	Overall	rs194809	A	G	0.187	0.061	0.011	4.9E-09	32.8
BMI	Overall	rs2342892	T	G	0.486	0.061	0.008	1.3E-13	54.9
BMI	Overall	rs7498665	A	G	0.618	-0.137	0.008	1.1E-66	281.1
BMI	Overall	rs3814883	T	C	0.480	0.109	0.008	1.5E-40	178.3
BMI	Overall	rs2193101	C	G	0.195	0.068	0.010	3.3E-11	45.7
BMI	Overall	rs1477199	A	G	0.843	-0.106	0.011	5.1E-21	92.3
BMI	Overall	rs9922708	T	C	0.440	0.321	0.008	0.0E+00	1743.1
BMI	Overall	rs2962449	T	C	0.471	0.053	0.008	6.7E-11	42.6
BMI	Overall	rs889398	T	C	0.415	-0.094	0.008	3.2E-32	148.5
BMI	Overall	rs17604662	A	G	0.890	-0.086	0.013	1.0E-10	40.9
BMI	Overall	rs756717	A	G	0.394	-0.064	0.008	2.4E-15	62.1
BMI	Overall	rs4390583	A	C	0.571	-0.048	0.009	1.6E-08	30.9
BMI	Overall	rs12922346	C	G	0.264	0.064	0.010	1.5E-11	44.2
BMI	Overall	rs7206608	C	G	0.675	-0.062	0.009	1.2E-12	52.2
BMI	Overall	rs3923783	A	C	0.178	-0.107	0.011	4.1E-23	101.8
BMI	Overall	rs11078883	C	G	0.654	-0.061	0.009	1.3E-12	49.0
BMI	Overall	rs5862	A	G	0.542	-0.049	0.008	2.3E-09	36.0
BMI	Overall	rs1075901	T	C	0.452	-0.057	0.008	4.4E-13	54.4
BMI	Overall	rs4986044	T	C	0.455	-0.085	0.008	1.3E-27	122.4
BMI	Overall	rs8065172	A	G	0.238	-0.060	0.010	5.3E-10	38.4
BMI	Overall	rs12150665	T	C	0.593	0.081	0.008	1.7E-24	110.3
BMI	Overall	rs6607337	T	C	0.298	-0.060	0.009	2.5E-11	42.6
BMI	Overall	rs7222349	A	G	0.356	0.048	0.009	1.7E-08	30.9
BMI	Overall	rs208015	T	C	0.070	0.169	0.016	7.2E-26	113.8
BMI	Overall	rs7207087	C	G	0.684	0.062	0.009	1.8E-12	52.2
BMI	Overall	rs11649864	A	G	0.086	0.092	0.014	2.5E-10	41.0
BMI	Overall	rs12602912	T	C	0.212	0.080	0.010	2.9E-16	68.9
BMI	Overall	rs312750	A	G	0.499	0.047	0.008	2.6E-09	36.8
BMI	Overall	rs12939549	A	G	0.557	0.087	0.008	3.7E-28	126.6
BMI	Overall	rs4075482	A	C	0.368	-0.063	0.009	2.0E-13	53.8
BMI	Overall	rs8097672	A	T	0.855	-0.100	0.012	3.3E-18	75.8
BMI	Overall	rs477805	A	G	0.204	-0.064	0.010	3.3E-10	40.7
BMI	Overall	rs891387	T	C	0.495	0.100	0.008	9.3E-35	149.7
BMI	Overall	rs1945160	A	G	0.382	-0.050	0.009	5.7E-09	33.4
BMI	Overall	rs1941696	A	G	0.517	0.054	0.008	4.7E-11	43.4
BMI	Overall	rs474605	A	G	0.468	-0.060	0.008	2.7E-13	54.1
BMI	Overall	rs1356506	T	C	0.629	0.066	0.009	8.4E-15	57.9
BMI	Overall	rs2612576	A	T	0.295	-0.052	0.009	8.5E-09	32.3
BMI	Overall	rs784257	T	C	0.188	-0.071	0.011	3.1E-11	44.6
BMI	Overall	rs9951619	T	G	0.224	-0.073	0.009	2.3E-15	64.0
BMI	Overall	rs6567160	T	C	0.752	-0.265	0.009	7.8E-184	844.1
BMI	Overall	rs17066856	T	C	0.892	0.168	0.013	7.1E-38	168.0
BMI	Overall	rs543892	A	G	0.608	0.049	0.009	7.0E-09	31.5
BMI	Overall	rs17783165	T	C	0.671	-0.062	0.008	2.7E-13	56.7
BMI	Overall	rs11150911	A	C	0.285	0.057	0.009	3.7E-11	43.0
BMI	Overall	rs12981256	A	G	0.528	0.073	0.008	1.4E-18	78.9
BMI	Overall	rs895330	C	G	0.806	0.094	0.011	1.6E-19	79.4

BMI	Overall	rs273512	T	C	0.409	0.075	0.009	4.5E-19	75.1
BMI	Overall	rs17724992	A	G	0.720	0.083	0.009	5.2E-21	91.3
BMI	Overall	rs7258722	A	T	0.413	-0.096	0.008	1.8E-30	138.4
BMI	Overall	rs12611148	A	C	0.143	-0.066	0.012	1.4E-08	32.6
BMI	Overall	rs12462975	A	G	0.324	0.093	0.009	1.5E-25	115.0
BMI	Overall	rs7245985	T	G	0.796	0.057	0.010	1.9E-08	32.1
BMI	Overall	rs11882409	A	C	0.303	0.058	0.009	3.3E-10	40.6
BMI	Overall	rs185350	T	C	0.490	0.066	0.008	9.1E-17	73.3
BMI	Overall	rs769449	A	G	0.107	-0.124	0.013	4.4E-21	91.3
BMI	Overall	rs11672660	T	C	0.187	-0.162	0.010	6.8E-60	259.1
BMI	Overall	rs9304665	A	T	0.742	0.111	0.010	2.2E-31	132.3
BMI	Overall	rs12608738	A	G	0.712	0.061	0.009	3.5E-11	44.7
BMI	Overall	rs1884389	T	C	0.439	-0.052	0.008	3.7E-10	40.4
BMI	Overall	rs4813619	T	G	0.531	-0.051	0.009	1.7E-09	34.7
BMI	Overall	rs1884897	A	G	0.375	-0.088	0.008	2.7E-28	117.1
BMI	Overall	rs2423668	T	C	0.420	0.051	0.009	7.8E-09	34.7
BMI	Overall	rs852056	T	C	0.254	0.059	0.009	2.1E-10	41.9
BMI	Overall	rs1409818	T	C	0.107	0.094	0.013	2.6E-12	48.5
BMI	Overall	rs8122855	A	G	0.338	0.066	0.009	4.1E-14	57.9
BMI	Overall	rs6059578	C	G	0.668	0.050	0.009	6.0E-09	33.4
BMI	Overall	rs16989232	A	G	0.386	0.055	0.008	6.2E-12	45.8
BMI	Overall	rs11904898	A	G	0.234	-0.065	0.010	3.2E-11	46.2
BMI	Overall	rs2143624	A	G	0.361	0.045	0.008	2.1E-08	30.6
BMI	Overall	rs17201143	T	C	0.305	-0.054	0.009	1.1E-09	38.7
BMI	Overall	rs17806224	A	G	0.181	-0.125	0.011	7.9E-32	139.7
BMI	Overall	rs1304549	A	G	0.226	-0.057	0.010	1.4E-08	31.6
BMI	Overall	rs6010784	T	C	0.506	0.051	0.008	6.9E-11	43.9
BMI	Overall	rs6512302	C	G	0.739	0.064	0.010	1.5E-11	44.9
BMI	Overall	rs2064044	A	C	0.806	-0.059	0.010	1.0E-08	34.3
BMI	Overall	rs2832283	A	G	0.225	0.055	0.010	4.7E-09	33.1
BMI	Overall	rs13047416	C	G	0.626	0.073	0.009	6.9E-18	71.3
BMI	Overall	rs13049280	C	G	0.643	0.050	0.009	7.0E-09	32.7
BMI	Overall	rs2183588	A	G	0.345	-0.055	0.009	1.9E-10	40.1
BMI	Overall	rs427943	A	C	0.429	-0.085	0.008	3.6E-25	108.4
BMI	Overall	rs11538	A	G	0.828	-0.066	0.011	1.1E-09	36.0
BMI	Overall	rs2238799	A	G	0.619	0.049	0.009	8.6E-09	32.1
BMI	Overall	rs12628891	T	C	0.319	-0.055	0.009	5.9E-10	36.6
BMI	Overall	rs12628051	T	C	0.641	0.077	0.009	2.9E-19	80.0
BMI	Overall	rs738140	A	G	0.710	0.062	0.009	3.1E-12	51.4
BMI	Women	rs12044597	A	G	0.496	-0.066	0.011	2.3E-10	39.3
BMI	Women	rs4648360	T	C	0.447	-0.061	0.011	6.3E-09	33.3
BMI	Women	rs309517	A	T	0.670	-0.064	0.012	2.5E-08	30.7
BMI	Women	rs12027258	T	C	0.835	0.080	0.014	4.7E-08	30.6
BMI	Women	rs13374459	T	C	0.610	-0.065	0.011	8.2E-09	34.5
BMI	Women	rs2984618	T	G	0.443	0.080	0.011	2.2E-14	57.6
BMI	Women	rs657452	A	G	0.405	0.098	0.011	1.1E-19	85.1
BMI	Women	rs17424278	A	C	0.903	-0.101	0.018	3.7E-08	30.5
BMI	Women	rs1013293	A	G	0.411	-0.074	0.011	2.2E-11	44.8

BMI	Women	rs2568958	A	G	0.619	0.109	0.011	1.3E-24	106.5
BMI	Women	rs6669189	T	C	0.405	0.101	0.011	4.7E-21	91.1
BMI	Women	rs12729914	T	C	0.830	-0.115	0.013	8.6E-18	73.5
BMI	Women	rs2166171	T	C	0.623	-0.066	0.012	7.2E-09	32.6
BMI	Women	rs11165643	T	C	0.579	0.087	0.011	1.7E-16	67.7
BMI	Women	rs4421623	T	G	0.210	0.088	0.013	1.1E-11	46.4
BMI	Women	rs7550711	T	C	0.028	0.308	0.033	3.4E-21	88.9
BMI	Women	rs197374	T	C	0.405	0.082	0.011	2.8E-13	55.3
BMI	Women	rs3738476	A	C	0.877	-0.094	0.017	2.2E-08	31.0
BMI	Women	rs11264483	C	G	0.588	0.074	0.012	7.6E-11	41.2
BMI	Women	rs12564992	A	G	0.888	-0.100	0.017	2.4E-09	35.0
BMI	Women	rs543874	A	G	0.777	-0.277	0.013	8.7E-100	455.1
BMI	Women	rs10920678	A	G	0.424	0.078	0.011	7.5E-14	54.9
BMI	Women	rs10801377	C	G	0.420	0.071	0.011	1.9E-10	41.4
BMI	Women	rs2820295	A	G	0.325	0.103	0.012	8.0E-19	79.5
BMI	Women	rs17014375	T	G	0.870	-0.091	0.016	1.5E-08	31.2
BMI	Women	rs12042959	A	G	0.865	0.093	0.015	2.3E-09	36.8
BMI	Women	rs6548237	A	C	0.160	-0.277	0.014	4.4E-89	394.5
BMI	Women	rs7571496	A	G	0.743	0.070	0.012	1.4E-08	31.1
BMI	Women	rs7601870	A	G	0.453	-0.063	0.011	1.2E-08	32.9
BMI	Women	rs10182181	A	G	0.505	-0.174	0.011	5.0E-63	270.8
BMI	Women	rs10048652	T	C	0.450	0.065	0.011	3.5E-09	34.5
BMI	Women	rs579119	T	C	0.670	0.069	0.011	6.7E-10	39.2
BMI	Women	rs2346882	A	G	0.335	-0.076	0.012	6.6E-11	43.3
BMI	Women	rs7582359	A	G	0.331	-0.080	0.012	1.0E-11	44.6
BMI	Women	rs1861412	A	G	0.423	-0.103	0.011	9.1E-21	87.4
BMI	Women	rs6728037	A	G	0.386	0.100	0.011	6.5E-19	81.0
BMI	Women	rs11678302	T	G	0.787	-0.081	0.014	7.2E-09	33.6
BMI	Women	rs12714199	T	C	0.615	-0.070	0.011	5.1E-10	40.3
BMI	Women	rs11677607	T	C	0.246	-0.115	0.013	2.9E-19	79.0
BMI	Women	rs1457457	A	T	0.532	0.068	0.011	3.8E-10	38.1
BMI	Women	rs6739199	T	C	0.270	0.076	0.012	3.2E-10	40.4
BMI	Women	rs1984559	A	G	0.808	0.078	0.014	3.3E-08	29.5
BMI	Women	rs13002158	A	G	0.813	-0.100	0.014	1.2E-12	51.4
BMI	Women	rs12692596	T	C	0.361	0.070	0.011	9.7E-11	40.3
BMI	Women	rs12692738	T	C	0.764	-0.078	0.012	2.8E-10	38.8
BMI	Women	rs7588437	A	G	0.367	-0.085	0.011	8.4E-15	58.6
BMI	Women	rs7594237	A	T	0.277	-0.069	0.012	1.8E-08	32.7
BMI	Women	rs4482463	A	C	0.917	-0.175	0.020	5.2E-18	75.5
BMI	Women	rs11692326	T	C	0.230	0.075	0.012	2.0E-09	36.0
BMI	Women	rs4673553	T	G	0.555	-0.074	0.011	2.6E-11	44.3
BMI	Women	rs12612009	T	G	0.663	-0.068	0.012	3.8E-09	34.5
BMI	Women	rs6720868	T	C	0.306	0.081	0.012	4.6E-12	49.6
BMI	Women	rs1801282	C	G	0.897	-0.087	0.016	4.7E-08	30.4
BMI	Women	rs2470520	T	C	0.577	-0.061	0.011	4.2E-08	30.0
BMI	Women	rs4858223	T	C	0.326	-0.077	0.012	8.3E-11	41.5
BMI	Women	rs13062093	T	G	0.632	-0.066	0.012	8.0E-09	32.6
BMI	Women	rs1799923	A	G	0.111	-0.117	0.017	5.1E-12	48.2

BMI	Women	rs2681781	A	G	0.480	-0.110	0.011	1.6E-25	107.4
BMI	Women	rs1916801	A	T	0.620	0.082	0.011	1.8E-14	59.7
BMI	Women	rs1452075	T	C	0.732	0.076	0.012	1.5E-10	40.4
BMI	Women	rs925018	C	G	0.665	-0.068	0.011	9.8E-10	38.1
BMI	Women	rs7647242	T	C	0.394	0.070	0.011	3.9E-10	39.7
BMI	Women	rs13068138	T	C	0.192	0.111	0.013	3.6E-17	73.2
BMI	Women	rs1949197	A	G	0.401	0.067	0.011	3.0E-09	36.5
BMI	Women	rs1492014	T	C	0.568	-0.070	0.011	2.8E-10	39.7
BMI	Women	rs1436348	A	G	0.432	-0.064	0.011	6.3E-09	33.9
BMI	Women	rs1289736	T	C	0.543	0.067	0.011	1.4E-09	36.5
BMI	Women	rs17619973	A	G	0.936	0.116	0.021	1.3E-08	31.7
BMI	Women	rs7428670	C	G	0.269	0.097	0.012	3.1E-15	60.4
BMI	Women	rs7621025	T	C	0.261	-0.096	0.012	1.6E-15	64.0
BMI	Women	rs16851483	T	G	0.073	0.174	0.022	2.3E-15	62.3
BMI	Women	rs355754	T	C	0.604	-0.068	0.011	4.3E-10	38.1
BMI	Women	rs247975	T	C	0.466	-0.087	0.011	3.6E-15	62.6
BMI	Women	rs10513801	T	G	0.878	0.169	0.015	1.3E-27	121.0
BMI	Women	rs4677812	A	C	0.293	-0.069	0.012	9.4E-09	33.2
BMI	Women	rs1477887	A	G	0.441	-0.075	0.011	1.7E-11	46.0
BMI	Women	rs9291467	T	C	0.465	0.072	0.011	5.7E-11	42.5
BMI	Women	rs6448587	A	C	0.802	0.081	0.014	1.6E-08	31.4
BMI	Women	rs337637	A	G	0.358	-0.060	0.011	3.0E-08	29.5
BMI	Women	rs10938397	A	G	0.571	-0.151	0.011	1.7E-45	205.0
BMI	Women	rs2271046	A	T	0.694	-0.066	0.012	2.4E-08	30.0
BMI	Women	rs2192158	A	G	0.449	0.070	0.011	2.1E-10	40.3
BMI	Women	rs11945861	A	G	0.247	-0.072	0.013	3.1E-08	30.9
BMI	Women	rs10002111	A	G	0.224	0.081	0.013	9.3E-10	36.4
BMI	Women	rs4148155	A	G	0.887	0.098	0.017	6.2E-09	33.6
BMI	Women	rs12645001	A	G	0.356	-0.068	0.012	4.1E-09	34.5
BMI	Women	rs13107325	T	C	0.083	0.217	0.021	9.3E-25	105.5
BMI	Women	rs4834272	T	C	0.687	-0.069	0.011	8.2E-10	38.7
BMI	Women	rs1403846	T	C	0.833	0.078	0.014	1.6E-08	31.2
BMI	Women	rs4864201	T	C	0.346	0.063	0.011	6.3E-09	32.4
BMI	Women	rs10000502	T	G	0.367	-0.073	0.012	1.8E-10	39.6
BMI	Women	rs750090	T	C	0.624	0.075	0.012	7.4E-11	42.3
BMI	Women	rs13110266	A	G	0.403	-0.068	0.011	1.5E-10	41.7
BMI	Women	rs1491333	T	G	0.263	-0.071	0.012	9.9E-09	32.0
BMI	Women	rs6890310	A	G	0.294	-0.074	0.012	1.4E-09	37.9
BMI	Women	rs782971	A	G	0.754	-0.083	0.012	4.9E-12	47.3
BMI	Women	rs10471636	A	G	0.525	-0.082	0.012	4.7E-13	50.2
BMI	Women	rs2112347	T	G	0.630	0.149	0.011	2.1E-42	180.5
BMI	Women	rs6870983	T	C	0.206	-0.102	0.012	9.6E-16	66.5
BMI	Women	rs2161228	T	C	0.101	0.135	0.018	5.5E-14	57.7
BMI	Women	rs10473922	A	G	0.458	0.064	0.011	7.0E-09	33.4
BMI	Women	rs6885199	A	G	0.036	0.187	0.031	8.5E-10	37.1
BMI	Women	rs2161097	T	C	0.442	0.062	0.011	2.3E-08	31.5
BMI	Women	rs158186	A	G	0.166	-0.138	0.015	3.2E-20	85.7
BMI	Women	rs4895231	C	G	0.468	0.069	0.011	9.3E-10	38.7

BMI	Women	rs13174863	A	G	0.854	-0.130	0.015	4.5E-18	75.9
BMI	Women	rs4569924	T	C	0.435	0.076	0.011	7.4E-13	51.6
BMI	Women	rs6556810	T	C	0.534	-0.060	0.011	5.0E-08	29.1
BMI	Women	rs2053682	A	C	0.678	0.088	0.012	1.9E-13	53.6
BMI	Women	rs2228213	A	G	0.337	-0.071	0.011	9.2E-11	41.4
BMI	Women	rs11757278	T	C	0.695	0.065	0.012	4.8E-08	29.6
BMI	Women	rs3806114	A	G	0.694	-0.068	0.012	4.5E-09	34.5
BMI	Women	rs2178899	A	T	0.885	0.119	0.016	7.2E-14	56.5
BMI	Women	rs11751591	A	G	0.153	-0.089	0.015	1.2E-08	33.4
BMI	Women	rs2814992	A	G	0.667	-0.138	0.011	1.2E-35	155.7
BMI	Women	rs1579557	T	C	0.288	0.102	0.012	3.1E-17	71.9
BMI	Women	rs943005	T	C	0.147	0.191	0.014	2.0E-43	188.4
BMI	Women	rs2465043	A	G	0.351	-0.068	0.012	3.2E-09	35.0
BMI	Women	rs209416	A	T	0.324	0.080	0.012	8.7E-12	47.8
BMI	Women	rs13209872	C	G	0.345	-0.068	0.012	6.1E-09	35.0
BMI	Women	rs12202969	A	G	0.479	-0.070	0.011	1.9E-10	39.7
BMI	Women	rs1417665	T	C	0.802	0.096	0.014	5.7E-12	47.1
BMI	Women	rs768023	A	G	0.603	0.089	0.011	1.4E-16	70.7
BMI	Women	rs2357760	A	G	0.673	0.076	0.011	1.1E-11	47.8
BMI	Women	rs11754747	T	C	0.271	0.070	0.012	1.3E-08	31.5
BMI	Women	rs198665	A	C	0.394	0.064	0.011	4.4E-09	33.9
BMI	Women	rs2185027	A	C	0.698	-0.074	0.012	1.1E-10	41.2
BMI	Women	rs9478496	T	C	0.847	-0.082	0.015	4.6E-08	30.1
BMI	Women	rs13213408	A	T	0.847	0.095	0.016	4.3E-09	35.6
BMI	Women	rs11765458	T	C	0.781	0.076	0.014	4.1E-08	29.7
BMI	Women	rs836525	T	C	0.173	0.085	0.015	9.8E-09	32.2
BMI	Women	rs4307239	A	G	0.536	-0.068	0.011	1.3E-09	37.6
BMI	Women	rs11971098	A	G	0.909	-0.114	0.020	6.5E-09	33.4
BMI	Women	rs10499694	A	G	0.494	0.065	0.011	4.0E-10	38.2
BMI	Women	rs10237317	A	G	0.588	-0.066	0.011	6.8E-10	39.3
BMI	Women	rs2267812	A	C	0.807	-0.083	0.014	1.7E-09	35.2
BMI	Women	rs1167800	A	G	0.553	0.096	0.011	3.7E-19	82.6
BMI	Women	rs7805441	T	C	0.547	0.070	0.011	2.0E-10	40.3
BMI	Women	rs2528531	A	C	0.651	-0.070	0.012	1.3E-09	36.5
BMI	Women	rs13240600	A	G	0.835	0.083	0.014	9.7E-09	33.3
BMI	Women	rs2299383	T	C	0.395	0.087	0.011	2.7E-16	68.4
BMI	Women	rs7788008	A	G	0.430	-0.082	0.011	1.1E-13	55.3
BMI	Women	rs2045293	T	C	0.417	0.091	0.011	4.1E-16	67.5
BMI	Women	rs2289705	A	G	0.796	-0.072	0.012	1.5E-08	33.3
BMI	Women	rs2968864	T	C	0.756	0.078	0.013	9.3E-10	36.4
BMI	Women	rs4240673	T	C	0.456	0.085	0.011	1.0E-15	64.0
BMI	Women	rs2959592	T	C	0.809	0.082	0.013	6.3E-10	36.9
BMI	Women	rs1362910	A	G	0.413	0.064	0.011	3.0E-09	33.9
BMI	Women	rs4320543	A	G	0.419	-0.062	0.011	2.9E-08	31.5
BMI	Women	rs12681792	A	C	0.204	0.088	0.014	2.6E-10	39.8
BMI	Women	rs1431659	A	G	0.274	0.094	0.012	2.9E-14	56.8
BMI	Women	rs17405819	T	C	0.683	0.105	0.012	7.8E-20	82.5
BMI	Women	rs12680842	A	G	0.684	0.062	0.012	3.4E-08	29.3

BMI	Women	rs2721965	A	C	0.669	0.089	0.012	2.1E-14	60.1
BMI	Women	rs10099330	A	G	0.538	-0.063	0.011	9.9E-09	32.4
BMI	Women	rs10124645	A	G	0.592	0.065	0.011	5.0E-09	35.0
BMI	Women	rs10756714	A	G	0.555	0.100	0.011	1.5E-19	81.8
BMI	Women	rs10962550	C	G	0.174	0.092	0.014	1.7E-10	41.0
BMI	Women	rs16912921	A	C	0.313	0.120	0.011	1.6E-26	117.2
BMI	Women	rs973345	T	C	0.611	0.068	0.011	1.1E-09	38.1
BMI	Women	rs563132	A	T	0.605	0.087	0.012	2.0E-14	56.3
BMI	Women	rs10797115	T	C	0.532	0.077	0.011	4.1E-12	48.4
BMI	Women	rs10992867	A	G	0.269	0.089	0.012	8.9E-13	50.6
BMI	Women	rs10118701	A	G	0.672	-0.085	0.012	2.2E-13	53.8
BMI	Women	rs7024334	T	G	0.226	0.076	0.012	1.6E-09	37.4
BMI	Women	rs1928295	T	C	0.571	0.077	0.011	2.8E-13	52.9
BMI	Women	rs3829849	T	C	0.350	0.065	0.011	1.9E-09	35.0
BMI	Women	rs10733682	A	G	0.458	0.076	0.011	1.9E-12	51.6
BMI	Women	rs2267958	A	G	0.510	-0.077	0.012	1.9E-11	45.0
BMI	Women	rs1009473	T	C	0.526	-0.061	0.011	3.4E-08	30.5
BMI	Women	rs7084454	A	G	0.306	0.108	0.012	7.4E-20	81.0
BMI	Women	rs12762034	T	C	0.928	-0.123	0.021	2.3E-09	35.2
BMI	Women	rs1624134	C	G	0.392	0.062	0.011	3.5E-08	31.0
BMI	Women	rs10823897	A	T	0.416	0.071	0.011	2.1E-10	40.8
BMI	Women	rs10761785	T	G	0.514	-0.083	0.011	4.2E-15	61.1
BMI	Women	rs10824347	A	T	0.771	0.085	0.013	5.5E-11	42.5
BMI	Women	rs7899106	A	G	0.950	-0.180	0.024	7.6E-14	56.3
BMI	Women	rs563296	A	G	0.558	0.084	0.012	8.8E-14	53.2
BMI	Women	rs3977755	T	C	0.287	-0.069	0.012	8.5E-09	32.7
BMI	Women	rs6585198	A	G	0.444	-0.074	0.011	1.2E-12	49.0
BMI	Women	rs1681743	T	C	0.401	-0.063	0.012	3.7E-08	29.8
BMI	Women	rs1561589	A	G	0.361	0.076	0.011	4.2E-12	47.2
BMI	Women	rs4880341	T	C	0.572	-0.069	0.011	4.5E-10	38.7
BMI	Women	rs7395632	T	C	0.357	-0.062	0.012	3.9E-08	29.3
BMI	Women	rs4929923	T	C	0.337	-0.106	0.011	2.5E-22	92.3
BMI	Women	rs11022762	T	C	0.368	-0.074	0.012	5.6E-11	41.2
BMI	Women	rs6265	T	C	0.183	-0.177	0.013	8.0E-40	172.7
BMI	Women	rs2862961	A	G	0.699	-0.115	0.012	5.3E-22	91.4
BMI	Women	rs10742752	T	C	0.375	-0.063	0.011	4.0E-09	36.0
BMI	Women	rs7124681	A	C	0.418	0.119	0.011	9.3E-29	126.1
BMI	Women	rs893006	A	C	0.721	0.083	0.012	4.0E-13	52.0
BMI	Women	rs7102454	T	C	0.643	-0.080	0.012	5.1E-12	47.8
BMI	Women	rs2605603	A	G	0.481	-0.063	0.011	1.4E-09	36.0
BMI	Women	rs7105462	A	G	0.581	0.069	0.011	4.4E-10	39.2
BMI	Women	rs1048932	A	C	0.440	-0.087	0.011	2.5E-16	68.4
BMI	Women	rs1003081	T	C	0.435	0.069	0.011	6.3E-11	42.8
BMI	Women	rs4459316	T	C	0.551	-0.074	0.011	2.3E-11	44.8
BMI	Women	rs4936175	T	C	0.559	-0.076	0.011	6.7E-12	47.2
BMI	Women	rs329651	T	G	0.805	0.087	0.013	2.0E-10	41.8
BMI	Women	rs12364470	T	G	0.855	-0.096	0.014	2.5E-11	44.4
BMI	Women	rs11611246	T	G	0.199	0.102	0.013	1.3E-14	61.7

BMI	Women	rs7976757	T	C	0.177	-0.092	0.014	2.9E-10	41.0
BMI	Women	rs16926318	A	G	0.921	-0.109	0.020	4.2E-08	30.7
BMI	Women	rs16926778	C	G	0.077	0.125	0.021	1.4E-09	36.6
BMI	Women	rs7138803	A	G	0.388	0.140	0.011	9.7E-38	160.1
BMI	Women	rs2271189	A	G	0.396	-0.070	0.012	6.4E-10	37.0
BMI	Women	rs11173522	A	C	0.233	0.081	0.013	9.0E-10	36.4
BMI	Women	rs645026	A	G	0.744	-0.077	0.012	4.8E-10	37.9
BMI	Women	rs2279574	A	C	0.549	-0.079	0.011	2.0E-12	51.5
BMI	Women	rs2712643	A	C	0.239	-0.074	0.012	1.5E-09	37.9
BMI	Women	rs17033633	T	C	0.169	-0.100	0.014	7.3E-12	48.5
BMI	Women	rs6606686	C	G	0.698	-0.086	0.011	2.5E-14	59.9
BMI	Women	rs1466852	A	G	0.227	0.078	0.013	3.6E-09	33.9
BMI	Women	rs6489156	T	C	0.734	0.107	0.012	1.0E-17	72.9
BMI	Women	rs7133378	A	G	0.334	0.076	0.012	1.9E-11	43.3
BMI	Women	rs1967772	A	G	0.271	-0.079	0.012	1.1E-10	40.3
BMI	Women	rs1045411	T	C	0.283	-0.067	0.012	2.6E-08	30.9
BMI	Women	rs4942925	T	C	0.574	0.062	0.011	2.8E-09	34.9
BMI	Women	rs12429545	A	G	0.120	0.140	0.016	1.1E-18	77.8
BMI	Women	rs6561937	A	T	0.767	-0.095	0.013	6.0E-13	53.2
BMI	Women	rs9538162	T	C	0.579	0.069	0.011	6.3E-10	38.7
BMI	Women	rs9571687	A	C	0.342	-0.070	0.012	3.2E-09	36.5
BMI	Women	rs1441264	A	G	0.583	0.082	0.011	5.2E-14	55.3
BMI	Women	rs9531786	C	G	0.371	-0.066	0.012	8.4E-09	33.1
BMI	Women	rs4343164	T	C	0.276	0.072	0.012	4.0E-09	33.3
BMI	Women	rs1927790	T	C	0.608	-0.065	0.011	1.3E-09	37.7
BMI	Women	rs4586287	C	G	0.674	0.066	0.012	2.9E-08	30.5
BMI	Women	rs7335249	T	C	0.558	0.065	0.011	4.9E-09	34.5
BMI	Women	rs2479958	A	G	0.486	0.065	0.012	1.4E-08	31.6
BMI	Women	rs2754079	A	G	0.571	-0.075	0.011	2.3E-11	46.0
BMI	Women	rs1569979	A	G	0.774	0.111	0.013	1.6E-17	72.6
BMI	Women	rs17522122	T	G	0.467	0.080	0.011	6.5E-14	57.6
BMI	Women	rs2806034	T	G	0.668	0.066	0.011	5.3E-09	35.5
BMI	Women	rs7141420	T	C	0.549	0.101	0.011	7.8E-22	91.1
BMI	Women	rs1285997	C	G	0.288	-0.072	0.012	3.2E-09	36.0
BMI	Women	rs9989141	T	C	0.616	0.081	0.011	3.2E-13	53.4
BMI	Women	rs3803286	A	G	0.341	0.083	0.012	8.3E-13	51.4
BMI	Women	rs709400	A	G	0.621	0.088	0.011	4.4E-16	64.0
BMI	Women	rs12101625	C	G	0.614	0.066	0.012	5.5E-09	33.1
BMI	Women	rs8038522	T	G	0.413	-0.067	0.011	1.3E-09	37.1
BMI	Women	rs6493498	T	C	0.446	0.078	0.011	1.5E-13	54.9
BMI	Women	rs8024806	T	C	0.937	0.131	0.023	9.8E-09	33.5
BMI	Women	rs8033510	T	C	0.364	0.066	0.012	1.1E-08	32.6
BMI	Women	rs4776982	A	G	0.769	0.149	0.012	4.2E-33	143.1
BMI	Women	rs12914489	A	G	0.081	0.109	0.017	1.6E-10	39.8
BMI	Women	rs12593036	A	G	0.690	0.074	0.012	6.6E-10	37.9
BMI	Women	rs11073383	A	G	0.480	-0.076	0.011	3.3E-12	47.8
BMI	Women	rs214249	T	G	0.607	0.080	0.012	1.4E-12	48.4
BMI	Women	rs12448257	A	G	0.219	0.072	0.013	3.1E-08	30.9

BMI	Women	rs879620	T	C	0.596	0.119	0.011	5.4E-26	115.3
BMI	Women	rs9926784	T	C	0.800	0.121	0.013	6.0E-20	81.0
BMI	Women	rs17767765	T	C	0.515	-0.061	0.011	2.6E-08	30.5
BMI	Women	rs7498665	A	G	0.619	-0.127	0.011	1.9E-32	144.0
BMI	Women	rs2289292	T	C	0.332	-0.091	0.012	1.1E-14	57.8
BMI	Women	rs4889606	A	G	0.631	0.129	0.011	1.3E-32	135.8
BMI	Women	rs1558902	A	T	0.409	0.337	0.011	1.1E-215	1018.2
BMI	Women	rs4783718	T	C	0.419	0.098	0.011	1.8E-19	78.7
BMI	Women	rs811054	T	C	0.533	0.078	0.011	3.2E-13	54.2
BMI	Women	rs2012502	A	C	0.384	0.074	0.011	3.0E-11	44.3
BMI	Women	rs4790292	A	C	0.147	-0.126	0.016	1.7E-15	63.5
BMI	Women	rs3826408	T	C	0.418	0.058	0.011	4.5E-08	30.3
BMI	Women	rs1075901	T	C	0.452	-0.069	0.011	4.4E-11	42.8
BMI	Women	rs4986044	T	C	0.453	-0.083	0.011	2.2E-15	61.8
BMI	Women	rs12150665	T	C	0.592	0.080	0.011	5.9E-14	57.6
BMI	Women	rs9906944	T	C	0.355	-0.091	0.012	7.3E-15	62.7
BMI	Women	rs7211966	T	C	0.029	-0.226	0.038	4.9E-09	34.5
BMI	Women	rs8075273	A	C	0.290	-0.077	0.012	5.6E-11	44.4
BMI	Women	rs2619976	T	C	0.399	0.070	0.012	6.2E-10	37.0
BMI	Women	rs12949279	T	C	0.563	0.086	0.011	6.1E-16	66.2
BMI	Women	rs4969387	C	G	0.738	-0.074	0.013	6.9E-09	32.5
BMI	Women	rs9955276	T	C	0.144	0.107	0.015	8.0E-12	48.1
BMI	Women	rs1808579	T	C	0.481	-0.095	0.011	9.4E-20	81.0
BMI	Women	rs16975921	A	T	0.695	-0.078	0.012	3.9E-11	42.5
BMI	Women	rs1518159	T	C	0.250	-0.091	0.012	6.7E-13	52.8
BMI	Women	rs7239114	A	G	0.542	0.075	0.011	3.9E-12	49.6
BMI	Women	rs6567160	T	C	0.751	-0.273	0.012	8.7E-108	475.6
BMI	Women	rs17066856	T	C	0.891	0.191	0.018	1.4E-27	115.7
BMI	Women	rs12981256	A	G	0.527	0.087	0.011	3.9E-15	62.6
BMI	Women	rs895330	C	G	0.807	0.087	0.014	6.6E-10	39.0
BMI	Women	rs273504	A	G	0.576	-0.073	0.011	1.3E-10	43.1
BMI	Women	rs17724992	A	G	0.719	0.082	0.012	6.7E-12	46.2
BMI	Women	rs11880870	A	G	0.519	0.095	0.011	3.3E-18	73.4
BMI	Women	rs17751061	T	C	0.159	-0.087	0.015	1.3E-08	32.0
BMI	Women	rs12462975	A	G	0.325	0.098	0.012	2.6E-16	65.9
BMI	Women	rs11667280	C	G	0.797	0.076	0.014	3.1E-08	29.7
BMI	Women	rs7250833	T	C	0.311	0.072	0.012	7.2E-10	38.5
BMI	Women	rs185350	T	C	0.490	0.062	0.011	7.1E-09	33.9
BMI	Women	rs11672660	T	C	0.187	-0.154	0.013	5.9E-31	131.4
BMI	Women	rs9304665	A	T	0.740	0.098	0.013	1.8E-14	57.1
BMI	Women	rs1884389	T	C	0.440	-0.067	0.011	1.2E-09	37.1
BMI	Women	rs1884897	A	G	0.375	-0.081	0.011	9.1E-14	54.0
BMI	Women	rs1409818	T	C	0.108	0.121	0.018	1.6E-11	46.4
BMI	Women	rs6050446	A	G	0.032	-0.179	0.031	6.2E-09	33.8
BMI	Women	rs6142096	A	G	0.520	0.064	0.011	4.2E-09	33.9
BMI	Women	rs16989232	A	G	0.385	0.062	0.011	1.5E-08	31.0
BMI	Women	rs17806224	A	G	0.181	-0.139	0.014	3.5E-22	92.8
BMI	Women	rs6010784	T	C	0.508	0.061	0.011	1.1E-08	32.8

BMI	Women	rs2836754	T	C	0.369	-0.072	0.011	3.7E-11	45.9
BMI	Women	rs8126575	T	G	0.868	0.088	0.016	3.0E-08	31.1
BMI	Women	rs427943	A	C	0.428	-0.085	0.011	1.4E-14	59.2
BMI	Women	rs4820408	T	G	0.375	0.065	0.011	1.4E-09	38.2
BMI	Women	rs13055841	A	G	0.693	0.066	0.012	3.7E-08	30.5
BMI	Women	rs13053342	A	G	0.400	0.074	0.012	1.3E-10	40.6
BMI	Men	rs2803316	A	G	0.457	-0.072	0.012	4.0E-09	35.5
BMI	Men	rs3762444	T	C	0.461	-0.093	0.012	1.5E-14	60.2
BMI	Men	rs11121210	T	C	0.342	-0.072	0.012	7.9E-09	32.8
BMI	Men	rs11581010	A	G	0.763	-0.086	0.014	1.0E-09	37.7
BMI	Men	rs2235549	T	G	0.840	-0.102	0.016	8.3E-11	41.7
BMI	Men	rs2271928	A	G	0.375	-0.065	0.012	9.9E-09	32.1
BMI	Men	rs12061406	T	C	0.078	-0.131	0.022	1.7E-09	36.8
BMI	Men	rs1768808	T	C	0.624	0.069	0.012	3.5E-08	30.3
BMI	Men	rs977747	T	G	0.439	0.076	0.012	1.3E-11	43.9
BMI	Men	rs7531656	A	G	0.330	0.095	0.012	2.3E-15	62.7
BMI	Men	rs2481665	T	C	0.583	0.090	0.012	2.0E-15	60.7
BMI	Men	rs2310754	A	G	0.510	0.075	0.012	4.4E-10	38.4
BMI	Men	rs1993709	A	G	0.178	-0.199	0.014	1.5E-42	189.5
BMI	Men	rs12566985	A	G	0.558	-0.087	0.011	1.1E-14	61.9
BMI	Men	rs12049202	T	C	0.186	0.109	0.015	5.5E-13	53.1
BMI	Men	rs12034762	T	C	0.392	0.081	0.012	4.8E-11	41.8
BMI	Men	rs11165643	T	C	0.579	0.097	0.012	1.7E-17	70.1
BMI	Men	rs17024393	T	C	0.968	-0.342	0.034	1.7E-23	100.3
BMI	Men	rs6587552	A	G	0.241	0.086	0.014	1.0E-09	37.7
BMI	Men	rs543874	A	G	0.775	-0.173	0.014	3.7E-35	154.1
BMI	Men	rs10920678	A	G	0.422	0.064	0.012	1.9E-08	30.7
BMI	Men	rs2172935	T	C	0.321	0.128	0.012	3.6E-24	105.5
BMI	Men	rs823094	T	G	0.553	0.071	0.012	3.8E-10	38.0
BMI	Men	rs4653942	A	G	0.209	-0.077	0.014	4.4E-08	30.4
BMI	Men	rs2125231	T	C	0.323	0.082	0.012	5.5E-11	43.3
BMI	Men	rs4639527	A	G	0.704	-0.096	0.013	2.0E-13	54.9
BMI	Men	rs12714415	T	C	0.850	0.290	0.015	1.1E-80	356.3
BMI	Men	rs2693826	A	G	0.429	-0.079	0.012	5.0E-11	43.0
BMI	Men	rs10182181	A	G	0.505	-0.131	0.011	5.5E-32	140.9
BMI	Men	rs1260326	T	C	0.392	-0.088	0.012	2.1E-14	58.1
BMI	Men	rs2888172	A	G	0.547	-0.068	0.012	1.4E-08	31.8
BMI	Men	rs10185199	A	G	0.287	-0.076	0.014	3.8E-08	30.1
BMI	Men	rs7561278	T	C	0.764	0.084	0.015	1.1E-08	31.9
BMI	Men	rs12986742	T	C	0.517	-0.095	0.012	3.2E-15	62.1
BMI	Men	rs887912	T	C	0.299	0.110	0.012	2.7E-19	77.6
BMI	Men	rs11685481	T	C	0.850	0.095	0.016	9.0E-09	33.6
BMI	Men	rs4581940	T	C	0.586	0.075	0.012	8.4E-10	38.4
BMI	Men	rs7557796	T	C	0.361	0.075	0.012	2.2E-09	35.5
BMI	Men	rs1437377	T	G	0.854	-0.117	0.017	1.5E-11	45.9
BMI	Men	rs10197031	T	C	0.729	-0.081	0.013	7.7E-10	38.7
BMI	Men	rs902695	A	G	0.492	-0.072	0.012	2.3E-09	35.5
BMI	Men	rs11687120	A	G	0.446	0.066	0.012	4.7E-08	30.0

BMI	Men	rs961360	A	G	0.890	0.102	0.016	3.8E-10	39.2
BMI	Men	rs453520	T	C	0.566	-0.077	0.012	1.2E-10	41.5
BMI	Men	rs908671	C	G	0.709	0.077	0.013	5.8E-09	33.1
BMI	Men	rs1019612	T	C	0.664	0.086	0.012	1.4E-11	47.4
BMI	Men	rs10497807	C	G	0.502	-0.070	0.011	5.0E-10	40.3
BMI	Men	rs7569376	T	C	0.207	0.094	0.014	1.3E-10	42.3
BMI	Men	rs17203016	A	G	0.802	-0.082	0.014	9.9E-09	32.1
BMI	Men	rs7599312	A	G	0.275	-0.105	0.012	1.5E-16	70.3
BMI	Men	rs1541777	A	G	0.551	-0.075	0.011	1.8E-11	46.0
BMI	Men	rs7600417	A	G	0.853	0.100	0.017	2.9E-09	35.3
BMI	Men	rs11692572	C	G	0.596	-0.067	0.012	3.7E-08	30.9
BMI	Men	rs2596902	A	G	0.650	-0.072	0.012	1.3E-08	33.3
BMI	Men	rs6804842	A	G	0.430	-0.087	0.012	1.3E-14	57.5
BMI	Men	rs762318	A	C	0.628	0.074	0.012	2.1E-09	34.6
BMI	Men	rs1524887	T	C	0.419	-0.067	0.012	3.6E-08	30.9
BMI	Men	rs6803741	T	C	0.664	-0.072	0.013	2.0E-08	30.9
BMI	Men	rs2188151	T	G	0.415	0.137	0.012	2.7E-29	129.0
BMI	Men	rs6787805	A	G	0.139	-0.099	0.017	2.3E-09	34.6
BMI	Men	rs2365389	T	C	0.395	-0.085	0.012	5.3E-14	54.4
BMI	Men	rs11915371	A	C	0.804	-0.105	0.015	1.1E-12	49.5
BMI	Men	rs13078509	T	C	0.028	0.231	0.040	8.4E-09	32.8
BMI	Men	rs3849570	A	C	0.354	0.072	0.012	2.6E-09	35.5
BMI	Men	rs12495178	T	C	0.641	0.109	0.012	1.5E-20	88.7
BMI	Men	rs7628689	A	G	0.169	-0.110	0.016	1.4E-11	45.4
BMI	Men	rs1492014	T	C	0.568	-0.102	0.012	1.6E-17	72.6
BMI	Men	rs1436351	T	G	0.737	0.086	0.013	3.4E-11	43.5
BMI	Men	rs6768228	A	G	0.632	-0.072	0.012	6.8E-09	32.8
BMI	Men	rs2918217	T	C	0.153	0.106	0.017	7.2E-10	37.7
BMI	Men	rs1320903	A	G	0.309	0.123	0.013	1.7E-21	89.2
BMI	Men	rs645040	T	G	0.770	0.090	0.013	3.2E-11	44.6
BMI	Men	rs2035935	A	G	0.924	-0.160	0.022	4.0E-13	52.4
BMI	Men	rs355777	C	G	0.397	0.082	0.012	1.8E-11	46.2
BMI	Men	rs9870575	A	G	0.141	-0.114	0.017	1.5E-11	45.9
BMI	Men	rs5398	A	G	0.283	0.081	0.012	5.5E-11	41.8
BMI	Men	rs2606227	T	C	0.368	0.068	0.012	4.6E-08	29.8
BMI	Men	rs9816226	A	T	0.174	-0.164	0.015	5.1E-29	121.0
BMI	Men	rs6841761	T	G	0.521	-0.064	0.011	9.0E-09	33.9
BMI	Men	rs2242189	T	C	0.633	0.082	0.012	5.6E-11	42.8
BMI	Men	rs13130484	T	C	0.431	0.169	0.012	6.4E-50	215.1
BMI	Men	rs2237025	T	C	0.430	0.068	0.012	3.1E-08	31.8
BMI	Men	rs13107325	T	C	0.085	0.238	0.023	3.2E-26	110.9
BMI	Men	rs2613726	A	G	0.370	-0.078	0.012	4.3E-10	38.8
BMI	Men	rs2391518	T	C	0.315	0.076	0.012	1.7E-10	40.4
BMI	Men	rs1296328	A	C	0.443	0.096	0.012	2.1E-15	64.0
BMI	Men	rs6537285	A	G	0.280	-0.086	0.013	1.2E-10	40.4
BMI	Men	rs6827083	A	G	0.579	-0.073	0.012	1.3E-10	40.1
BMI	Men	rs828544	T	G	0.435	0.066	0.012	4.5E-08	30.0
BMI	Men	rs1503526	T	C	0.530	-0.067	0.012	5.7E-09	34.0

BMI	Men	rs253414	T	C	0.657	0.125	0.012	1.1E-24	107.3
BMI	Men	rs4532349	A	G	0.783	0.078	0.013	6.6E-09	33.5
BMI	Men	rs7733438	T	G	0.859	-0.166	0.017	2.1E-21	91.8
BMI	Men	rs6235	C	G	0.734	-0.100	0.013	1.9E-13	54.7
BMI	Men	rs11951673	T	C	0.395	-0.090	0.012	7.8E-15	60.7
BMI	Men	rs289228	C	G	0.836	0.107	0.016	9.8E-12	45.3
BMI	Men	rs7711753	A	G	0.416	-0.063	0.012	2.6E-08	30.3
BMI	Men	rs7715256	T	G	0.563	-0.078	0.012	4.5E-12	46.1
BMI	Men	rs7730898	A	G	0.731	0.081	0.012	9.9E-11	42.3
BMI	Men	rs6556301	T	G	0.370	-0.069	0.012	1.6E-08	32.7
BMI	Men	rs2228213	A	G	0.337	-0.073	0.012	6.8E-10	36.5
BMI	Men	rs9366639	C	G	0.804	0.093	0.016	3.8E-09	34.6
BMI	Men	rs2178899	A	T	0.886	0.118	0.017	5.4E-12	46.7
BMI	Men	rs14398	A	G	0.661	-0.107	0.017	7.1E-10	38.0
BMI	Men	rs2744974	T	C	0.322	0.110	0.012	8.5E-20	83.9
BMI	Men	rs17681686	C	G	0.295	0.101	0.013	5.3E-15	60.5
BMI	Men	rs1358980	T	C	0.472	-0.075	0.012	6.1E-11	42.8
BMI	Men	rs2206277	T	C	0.151	0.218	0.015	5.4E-50	214.5
BMI	Men	rs1327259	A	G	0.610	0.076	0.012	6.0E-10	37.4
BMI	Men	rs7773045	T	G	0.922	0.127	0.021	1.6E-09	36.0
BMI	Men	rs12663742	A	G	0.372	-0.087	0.012	1.7E-12	48.5
BMI	Men	rs10872224	T	G	0.585	0.097	0.012	3.8E-15	59.8
BMI	Men	rs17789218	T	C	0.753	-0.073	0.013	3.3E-08	29.5
BMI	Men	rs768023	A	G	0.602	0.064	0.012	3.0E-08	30.7
BMI	Men	rs1159974	T	C	0.492	-0.067	0.012	2.0E-08	30.9
BMI	Men	rs6569648	T	C	0.765	-0.073	0.013	3.7E-08	29.5
BMI	Men	rs765875	T	C	0.466	-0.066	0.012	3.4E-08	30.5
BMI	Men	rs13191362	A	G	0.853	0.127	0.017	2.3E-13	54.2
BMI	Men	rs9364687	T	G	0.398	-0.067	0.012	4.0E-09	34.0
BMI	Men	rs9638713	A	G	0.027	0.216	0.036	2.3E-09	36.0
BMI	Men	rs215635	T	C	0.622	-0.088	0.012	1.2E-12	49.5
BMI	Men	rs7779853	A	G	0.461	-0.063	0.012	2.8E-08	29.8
BMI	Men	rs10269783	A	G	0.423	0.083	0.012	7.6E-13	51.4
BMI	Men	rs1167827	A	G	0.439	-0.104	0.012	2.9E-19	81.8
BMI	Men	rs2245368	T	C	0.810	-0.158	0.016	4.4E-23	98.8
BMI	Men	rs1965529	A	G	0.771	0.084	0.015	4.2E-08	29.9
BMI	Men	rs1701829	A	G	0.336	0.103	0.012	6.8E-16	67.7
BMI	Men	rs13228237	C	G	0.213	-0.079	0.014	4.6E-08	29.9
BMI	Men	rs4841504	A	C	0.502	-0.093	0.012	4.2E-16	64.7
BMI	Men	rs4123853	T	C	0.373	0.080	0.012	1.1E-10	40.8
BMI	Men	rs4258002	T	G	0.141	-0.089	0.016	2.0E-08	31.4
BMI	Men	rs7827210	A	G	0.397	0.086	0.012	2.3E-12	47.4
BMI	Men	rs7012923	A	C	0.441	0.078	0.012	4.9E-11	42.5
BMI	Men	rs2017657	T	G	0.559	-0.074	0.012	7.0E-10	37.9
BMI	Men	rs2588785	A	G	0.284	-0.101	0.012	1.7E-15	65.9
BMI	Men	rs957447	A	C	0.239	-0.108	0.014	8.6E-14	56.3
BMI	Men	rs800571	T	C	0.246	-0.087	0.014	6.9E-10	39.0
BMI	Men	rs11781699	T	C	0.811	-0.085	0.014	6.7E-09	34.4

BMI	Men	rs7826388	T	C	0.037	-0.180	0.032	1.4E-08	32.1
BMI	Men	rs4740619	T	C	0.542	0.090	0.011	1.5E-15	66.1
BMI	Men	rs10738451	T	C	0.715	-0.078	0.013	2.2E-09	36.4
BMI	Men	rs10968114	A	C	0.534	0.074	0.012	8.7E-10	37.5
BMI	Men	rs1412235	C	G	0.314	0.112	0.012	9.9E-21	87.6
BMI	Men	rs10746862	T	G	0.580	-0.075	0.012	8.6E-10	38.4
BMI	Men	rs1187351	A	C	0.399	-0.067	0.012	4.5E-08	30.9
BMI	Men	rs6559921	A	T	0.771	0.087	0.014	1.2E-09	36.4
BMI	Men	rs6477694	T	C	0.642	-0.067	0.012	8.9E-09	34.0
BMI	Men	rs7038943	T	C	0.659	0.073	0.012	6.5E-09	34.2
BMI	Men	rs10760279	T	G	0.383	0.074	0.013	7.5E-09	32.5
BMI	Men	rs3902840	A	G	0.087	0.148	0.021	2.5E-12	48.7
BMI	Men	rs4740383	A	G	0.418	0.074	0.012	2.7E-09	35.1
BMI	Men	rs11251291	A	C	0.467	-0.068	0.012	1.7E-08	31.8
BMI	Men	rs10828247	A	G	0.664	-0.081	0.012	1.7E-10	42.3
BMI	Men	rs3758438	T	C	0.078	0.132	0.023	7.3E-09	34.0
BMI	Men	rs7072873	T	C	0.535	-0.071	0.012	5.5E-09	34.6
BMI	Men	rs10788494	C	G	0.483	0.075	0.012	5.4E-10	38.4
BMI	Men	rs17094222	T	C	0.791	-0.123	0.014	1.1E-18	77.3
BMI	Men	rs284851	C	G	0.879	-0.119	0.017	2.8E-12	50.2
BMI	Men	rs7903146	T	C	0.278	-0.111	0.012	3.6E-19	78.9
BMI	Men	rs845084	A	G	0.269	0.075	0.014	3.8E-08	29.3
BMI	Men	rs7113874	T	C	0.327	-0.069	0.012	3.3E-09	36.0
BMI	Men	rs900144	T	C	0.584	0.074	0.012	5.5E-10	37.9
BMI	Men	rs6265	T	C	0.183	-0.230	0.014	6.3E-58	253.9
BMI	Men	rs1765139	T	C	0.767	0.106	0.014	1.8E-13	54.3
BMI	Men	rs2862996	T	G	0.704	-0.096	0.012	2.5E-15	63.4
BMI	Men	rs7124681	A	C	0.419	0.132	0.012	5.1E-31	131.3
BMI	Men	rs7102454	T	C	0.642	-0.085	0.012	1.3E-11	46.3
BMI	Men	rs349088	A	C	0.473	-0.072	0.012	2.0E-09	36.0
BMI	Men	rs647248	A	G	0.544	0.077	0.012	2.6E-10	41.5
BMI	Men	rs12286929	A	G	0.507	-0.086	0.011	2.3E-14	59.9
BMI	Men	rs7944782	T	G	0.504	-0.070	0.012	7.5E-09	34.1
BMI	Men	rs10894670	A	C	0.561	-0.068	0.012	1.6E-09	35.0
BMI	Men	rs11611246	T	G	0.196	0.118	0.014	8.0E-17	67.2
BMI	Men	rs10772983	T	C	0.544	-0.067	0.011	2.0E-09	37.1
BMI	Men	rs10842240	C	G	0.125	0.124	0.018	4.6E-12	48.6
BMI	Men	rs11170468	A	C	0.784	0.077	0.013	7.8E-09	33.1
BMI	Men	rs11181001	A	G	0.482	0.069	0.012	1.2E-09	36.0
BMI	Men	rs7132908	A	G	0.392	0.166	0.013	1.3E-38	164.2
BMI	Men	rs705704	A	G	0.322	-0.078	0.012	1.5E-10	39.3
BMI	Men	rs1689437	A	G	0.948	-0.143	0.024	2.0E-09	36.7
BMI	Men	rs7488867	T	C	0.257	-0.106	0.014	5.7E-14	57.6
BMI	Men	rs10850184	T	G	0.321	0.080	0.013	3.5E-10	38.3
BMI	Men	rs12369179	T	C	0.084	-0.169	0.022	1.4E-14	58.2
BMI	Men	rs7323	C	G	0.267	-0.084	0.013	4.1E-10	39.1
BMI	Men	rs2504236	T	C	0.586	-0.073	0.012	1.9E-09	36.5
BMI	Men	rs9595893	T	G	0.360	-0.096	0.012	1.2E-14	58.6

BMI	Men	rs9603697	T	C	0.317	0.095	0.013	1.3E-13	53.2
BMI	Men	rs12429545	A	G	0.121	0.162	0.017	2.0E-21	92.2
BMI	Men	rs7338127	A	T	0.330	0.095	0.013	1.4E-13	53.2
BMI	Men	rs1441264	A	G	0.582	0.087	0.012	1.4E-13	56.9
BMI	Men	rs912690	C	G	0.557	-0.083	0.012	6.9E-12	47.9
BMI	Men	rs9168	A	C	0.273	-0.072	0.013	4.6E-08	30.9
BMI	Men	rs12868881	A	T	0.405	0.087	0.012	1.1E-12	51.8
BMI	Men	rs10132280	A	C	0.313	-0.144	0.012	1.4E-31	132.3
BMI	Men	rs1950709	A	G	0.556	-0.065	0.011	7.3E-09	35.0
BMI	Men	rs1955540	T	C	0.194	-0.096	0.015	2.9E-10	39.1
BMI	Men	rs4900714	T	G	0.473	0.096	0.012	1.2E-15	64.0
BMI	Men	rs217671	A	G	0.726	-0.081	0.013	2.2E-09	36.4
BMI	Men	rs3902951	T	G	0.771	-0.073	0.013	4.4E-08	29.1
BMI	Men	rs17109256	A	G	0.237	0.148	0.013	2.8E-27	121.0
BMI	Men	rs12888545	A	G	0.745	-0.084	0.014	2.0E-09	36.4
BMI	Men	rs2148564	A	G	0.285	-0.095	0.013	1.1E-12	50.0
BMI	Men	rs7161194	A	G	0.345	0.103	0.013	3.3E-15	63.4
BMI	Men	rs2015407	A	G	0.353	0.087	0.012	2.7E-12	47.9
BMI	Men	rs11636536	T	C	0.497	0.071	0.012	2.8E-09	35.0
BMI	Men	rs10851458	A	G	0.581	0.071	0.012	6.0E-09	35.0
BMI	Men	rs4776970	A	T	0.643	0.134	0.012	1.5E-30	135.1
BMI	Men	rs4777541	T	C	0.784	0.101	0.014	1.1E-12	49.0
BMI	Men	rs8024572	A	G	0.611	0.075	0.012	7.8E-10	38.9
BMI	Men	rs8036171	A	C	0.374	0.074	0.012	4.3E-10	37.5
BMI	Men	rs12448257	A	G	0.219	0.089	0.014	1.2E-10	41.1
BMI	Men	rs879620	T	C	0.597	0.100	0.012	3.1E-16	64.6
BMI	Men	rs9926784	T	C	0.801	0.108	0.014	5.7E-14	55.8
BMI	Men	rs9921854	A	G	0.087	-0.140	0.022	1.9E-10	40.3
BMI	Men	rs3888190	A	C	0.380	0.151	0.012	7.0E-40	172.3
BMI	Men	rs7201780	T	C	0.454	-0.107	0.012	1.4E-18	79.6
BMI	Men	rs1558902	A	T	0.415	0.392	0.012	1.2E-257	1153.2
BMI	Men	rs889398	T	C	0.418	-0.100	0.012	1.5E-18	75.8
BMI	Men	rs756717	A	G	0.394	-0.068	0.012	8.6E-09	34.5
BMI	Men	rs7206608	C	G	0.674	-0.072	0.013	1.9E-08	30.9
BMI	Men	rs3923783	A	C	0.178	-0.109	0.016	7.5E-12	46.9
BMI	Men	rs12450045	A	G	0.458	0.069	0.012	1.7E-08	32.7
BMI	Men	rs4986044	T	C	0.454	-0.087	0.012	1.5E-14	56.9
BMI	Men	rs8065172	A	G	0.238	-0.080	0.014	1.2E-08	33.2
BMI	Men	rs12150665	T	C	0.593	0.087	0.012	3.4E-14	56.9
BMI	Men	rs676387	A	C	0.303	0.078	0.013	4.1E-09	36.0
BMI	Men	rs208015	T	C	0.070	0.175	0.024	8.7E-14	55.5
BMI	Men	rs12939549	A	G	0.556	0.096	0.011	1.3E-17	75.6
BMI	Men	rs7226371	A	G	0.844	-0.089	0.015	7.7E-09	33.8
BMI	Men	rs891387	T	C	0.496	0.109	0.012	6.8E-20	82.4
BMI	Men	rs11874191	T	C	0.728	-0.081	0.014	3.1E-09	34.0
BMI	Men	rs2612575	A	G	0.276	-0.077	0.013	1.5E-08	32.7
BMI	Men	rs9951619	T	G	0.222	-0.089	0.013	1.7E-11	44.1
BMI	Men	rs6567160	T	C	0.749	-0.256	0.013	2.1E-82	362.4

BMI	Men	rs17066856	T	C	0.890	0.145	0.019	2.7E-14	59.6
BMI	Men	rs17710386	T	C	0.664	-0.079	0.012	3.7E-11	43.0
BMI	Men	rs1787267	C	G	0.063	-0.157	0.025	2.5E-10	39.5
BMI	Men	rs7258309	T	G	0.784	0.112	0.015	6.5E-14	56.5
BMI	Men	rs273512	T	C	0.410	0.079	0.012	1.7E-10	39.8
BMI	Men	rs9636202	A	G	0.281	-0.085	0.013	5.5E-11	43.0
BMI	Men	rs7258722	A	T	0.411	-0.096	0.012	8.6E-15	58.6
BMI	Men	rs17513613	T	C	0.695	-0.086	0.012	1.5E-12	51.3
BMI	Men	rs11084553	A	G	0.849	0.114	0.017	9.4E-12	45.9
BMI	Men	rs769449	A	G	0.102	-0.116	0.019	1.6E-09	36.6
BMI	Men	rs1800437	C	G	0.186	-0.175	0.014	1.7E-33	148.0
BMI	Men	rs9304665	A	T	0.741	0.132	0.014	4.8E-21	89.3
BMI	Men	rs7249149	A	G	0.696	0.073	0.013	4.4E-08	29.5
BMI	Men	rs2145270	T	C	0.616	0.100	0.012	7.0E-18	75.1
BMI	Men	rs8123881	A	G	0.864	-0.092	0.016	2.5E-08	31.9
BMI	Men	rs7272093	A	G	0.700	-0.083	0.013	1.1E-09	37.7
BMI	Men	rs742760	A	T	0.820	0.112	0.015	7.1E-13	53.0
BMI	Men	rs6092143	T	C	0.368	0.072	0.012	1.1E-08	32.8
BMI	Men	rs13047416	C	G	0.628	0.075	0.012	9.8E-10	36.5
BMI	Men	rs427943	A	C	0.427	-0.084	0.012	2.2E-12	49.0
BMI	Men	rs138383	T	C	0.514	-0.075	0.012	1.4E-09	35.5
BMI	Men	rs733381	A	G	0.767	0.099	0.014	5.2E-13	50.5
HbA1c	Overall	rs11191561	G	C	0.150	0.122	0.022	2.3E-08	31.3
HbA1c	Overall	rs11195508	G	C	0.149	-0.119	0.022	4.3E-08	30.0
HbA1c	Overall	rs114322470	G	T	0.019	-0.363	0.057	2.2E-10	40.3
HbA1c	Overall	rs34872471	C	T	0.291	0.485	0.017	3.5E-177	806.4
HbA1c	Overall	rs10736269	C	T	0.471	-0.098	0.016	4.5E-10	38.9
HbA1c	Overall	rs11257655	T	C	0.208	0.245	0.019	1.5E-37	164.1
HbA1c	Overall	rs7904973	T	G	0.576	0.093	0.016	3.0E-09	35.2
HbA1c	Overall	rs11598987	G	C	0.527	0.088	0.016	1.8E-08	31.7
HbA1c	Overall	rs3847378	G	A	0.264	-0.116	0.018	5.3E-11	43.1
HbA1c	Overall	rs61850681	A	G	0.239	-0.227	0.018	3.2E-35	153.4
HbA1c	Overall	rs561279660	T	C	0.015	-0.690	0.068	3.7E-24	102.8
HbA1c	Overall	rs72814244	G	C	0.052	0.330	0.035	3.5E-21	89.2
HbA1c	Overall	rs150025254	T	C	0.030	-1.049	0.046	4.5E-114	515.7
HbA1c	Overall	rs75048187	G	A	0.013	0.610	0.070	3.6E-18	75.6
HbA1c	Overall	rs6480398	G	A	0.906	1.273	0.027	0.0E+00	2171.4
HbA1c	Overall	rs555721560	G	T	0.022	0.310	0.056	2.5E-08	31.1
HbA1c	Overall	rs138691176	A	G	0.033	0.267	0.045	4.0E-09	34.6
HbA1c	Overall	rs1248673	C	T	0.293	0.125	0.017	2.3E-13	53.7
HbA1c	Overall	rs701848	C	T	0.390	-0.121	0.016	3.3E-14	57.5
HbA1c	Overall	rs7923837	A	G	0.380	-0.196	0.016	1.7E-34	150.0
HbA1c	Overall	rs2068888	A	G	0.449	-0.116	0.016	1.2E-13	55.0
HbA1c	Overall	rs12365580	A	G	0.151	-0.218	0.022	1.3E-23	100.3
HbA1c	Overall	rs11042852	G	A	0.087	0.269	0.028	3.1E-22	94.0
HbA1c	Overall	rs3802885	C	A	0.075	-0.206	0.030	3.0E-12	48.7
HbA1c	Overall	rs6589939	G	A	0.379	0.112	0.016	2.9E-12	48.7
HbA1c	Overall	rs11512059	T	C	0.083	0.184	0.028	6.5E-11	42.7

HbA1c	Overall	rs7124355	G	A	0.675	-0.125	0.017	5.0E-14	56.7
HbA1c	Overall	rs689	T	A	0.710	-0.148	0.017	9.1E-18	73.7
HbA1c	Overall	rs12419995	T	A	0.656	-0.205	0.017	7.2E-33	142.6
HbA1c	Overall	rs2237895	C	A	0.415	0.185	0.016	5.3E-32	138.7
HbA1c	Overall	rs10047488	C	G	0.083	0.177	0.029	6.4E-10	38.2
HbA1c	Overall	rs4755309	T	G	0.114	-0.171	0.024	2.9E-12	48.8
HbA1c	Overall	rs10501320	C	G	0.264	-0.182	0.018	4.9E-25	106.8
HbA1c	Overall	rs174549	A	G	0.310	-0.167	0.017	2.1E-23	99.4
HbA1c	Overall	rs12294913	G	C	0.050	0.281	0.037	3.9E-14	57.2
HbA1c	Overall	rs1152620	G	A	0.262	0.120	0.018	1.1E-11	46.2
HbA1c	Overall	rs11602873	T	A	0.157	-0.236	0.021	1.2E-28	123.4
HbA1c	Overall	rs689419	G	T	0.782	0.114	0.019	1.6E-09	36.4
HbA1c	Overall	rs10830963	G	C	0.275	0.435	0.017	7.8E-139	629.7
HbA1c	Overall	rs3020069	A	G	0.683	0.106	0.017	1.7E-10	40.8
HbA1c	Overall	rs4910498	T	A	0.643	-0.224	0.016	2.4E-43	190.6
HbA1c	Overall	rs7310615	G	C	0.517	0.193	0.016	5.9E-35	152.2
HbA1c	Overall	rs2936839	C	T	0.610	-0.105	0.016	4.9E-11	43.2
HbA1c	Overall	rs73226260	A	G	0.034	-0.286	0.043	3.2E-11	44.1
HbA1c	Overall	rs11043299	C	T	0.621	0.104	0.017	1.5E-09	36.6
HbA1c	Overall	rs7306544	C	T	0.104	0.146	0.025	9.6E-09	32.9
HbA1c	Overall	rs704168	G	A	0.184	-0.123	0.020	9.7E-10	37.4
HbA1c	Overall	rs10492373	A	G	0.196	-0.177	0.019	1.3E-19	82.1
HbA1c	Overall	rs4238013	T	C	0.796	-0.122	0.019	3.6E-10	39.3
HbA1c	Overall	rs76895963	G	T	0.020	-1.275	0.060	2.1E-101	457.4
HbA1c	Overall	rs12819124	A	C	0.472	-0.417	0.016	2.3E-158	719.7
HbA1c	Overall	rs6580661	T	A	0.224	0.227	0.019	7.3E-34	147.2
HbA1c	Overall	rs11169605	C	T	0.358	0.125	0.016	1.3E-14	59.3
HbA1c	Overall	rs765634	G	A	0.367	-0.090	0.016	3.5E-08	30.4
HbA1c	Overall	rs79755767	A	G	0.100	-0.243	0.026	1.2E-20	86.7
HbA1c	Overall	rs4760278	A	C	0.226	-0.128	0.019	4.1E-12	48.1
HbA1c	Overall	rs2258238	T	A	0.105	0.218	0.025	8.2E-18	73.9
HbA1c	Overall	rs2137537	C	T	0.546	0.090	0.016	1.0E-08	32.8
HbA1c	Overall	rs12813389	T	A	0.539	0.091	0.016	5.5E-09	34.0
HbA1c	Overall	rs79621919	A	G	0.059	-0.261	0.033	2.9E-15	62.3
HbA1c	Overall	rs1044364	C	T	0.056	-0.191	0.034	2.0E-08	31.5
HbA1c	Overall	rs12876143	C	T	0.089	0.554	0.027	1.3E-91	412.4
HbA1c	Overall	rs368865	G	A	0.725	0.189	0.017	1.1E-27	119.0
HbA1c	Overall	rs7329468	T	C	0.217	-0.256	0.019	1.9E-41	181.9
HbA1c	Overall	rs4769580	A	T	0.219	0.224	0.019	1.9E-32	140.6
HbA1c	Overall	rs75629141	A	T	0.829	-0.209	0.021	5.8E-24	101.9
HbA1c	Overall	rs1327315	T	C	0.289	-0.134	0.017	5.7E-15	61.0
HbA1c	Overall	rs61990662	T	C	0.173	-0.127	0.021	9.5E-10	37.4
HbA1c	Overall	rs9324022	C	T	0.206	0.163	0.020	6.4E-17	69.9
HbA1c	Overall	rs12147688	T	G	0.258	0.145	0.018	4.5E-16	66.0
HbA1c	Overall	rs8013143	G	A	0.278	0.139	0.017	1.3E-15	64.0
HbA1c	Overall	rs7151822	C	T	0.247	-0.164	0.018	9.6E-20	82.7
HbA1c	Overall	rs72681698	C	T	0.011	-0.497	0.074	2.3E-11	44.7
HbA1c	Overall	rs11621337	A	C	0.063	-0.206	0.035	3.2E-09	35.0

HbA1c	Overall	rs2285005	A	G	0.340	0.182	0.016	1.5E-28	122.8
HbA1c	Overall	rs35889227	T	G	0.631	-0.150	0.016	9.5E-21	87.3
HbA1c	Overall	rs34715063	C	T	0.130	0.145	0.023	4.1E-10	39.1
HbA1c	Overall	rs28526689	A	G	0.344	0.101	0.016	6.3E-10	38.2
HbA1c	Overall	rs4516170	C	T	0.050	-0.325	0.036	6.6E-20	83.4
HbA1c	Overall	rs1550026	T	A	0.408	0.145	0.016	6.4E-20	83.5
HbA1c	Overall	rs59153558	G	A	0.720	0.200	0.017	1.0E-30	132.8
HbA1c	Overall	rs11638511	G	A	0.561	0.091	0.016	5.9E-09	33.9
HbA1c	Overall	rs7166540	G	A	0.238	-0.127	0.018	5.0E-12	47.7
HbA1c	Overall	rs1866476	C	T	0.732	-0.141	0.018	7.9E-16	64.9
HbA1c	Overall	rs61215894	C	T	0.558	-0.096	0.016	2.0E-09	36.0
HbA1c	Overall	rs4451969	C	T	0.671	-0.139	0.017	3.6E-17	71.0
HbA1c	Overall	rs35591	G	C	0.233	0.136	0.018	1.1E-13	55.3
HbA1c	Overall	rs112414002	T	C	0.026	0.281	0.049	1.0E-08	32.8
HbA1c	Overall	rs181207	T	C	0.336	0.164	0.016	2.5E-23	99.1
HbA1c	Overall	rs2858010	C	T	0.340	-0.169	0.016	1.1E-24	105.2
HbA1c	Overall	rs4889490	T	G	0.390	-0.108	0.016	1.0E-11	46.2
HbA1c	Overall	rs8050500	C	T	0.446	-0.180	0.016	1.1E-30	132.6
HbA1c	Overall	rs1121980	A	G	0.422	0.146	0.016	1.6E-20	86.3
HbA1c	Overall	rs35158985	G	A	0.308	0.159	0.017	2.9E-21	89.6
HbA1c	Overall	rs72802365	C	G	0.079	-0.193	0.029	2.3E-11	44.7
HbA1c	Overall	rs247826	T	C	0.223	0.185	0.019	4.6E-23	97.8
HbA1c	Overall	rs72811487	A	G	0.035	-0.386	0.042	4.3E-20	84.3
HbA1c	Overall	rs7195055	T	C	0.015	-0.392	0.065	1.8E-09	36.1
HbA1c	Overall	rs551118	G	C	0.578	0.382	0.016	9.0E-127	574.1
HbA1c	Overall	rs11655029	C	T	0.695	-0.223	0.017	1.0E-39	174.0
HbA1c	Overall	rs12943904	C	G	0.306	-0.097	0.017	7.6E-09	33.4
HbA1c	Overall	rs4985997	T	C	0.350	-0.136	0.016	7.0E-17	69.7
HbA1c	Overall	rs216195	G	T	0.303	0.137	0.017	4.8E-16	65.9
HbA1c	Overall	rs12450826	T	A	0.293	-0.186	0.017	1.7E-26	113.5
HbA1c	Overall	rs9894558	A	T	0.270	-0.115	0.018	4.2E-10	39.0
HbA1c	Overall	rs10908278	A	T	0.515	-0.123	0.016	3.5E-15	62.0
HbA1c	Overall	rs150325706	A	T	0.262	0.131	0.019	1.1E-11	46.2
HbA1c	Overall	rs7222851	C	T	0.512	-0.105	0.016	1.4E-11	45.7
HbA1c	Overall	rs35895680	A	C	0.327	-0.096	0.017	9.1E-09	33.0
HbA1c	Overall	rs12600858	A	G	0.224	-0.203	0.019	1.4E-27	118.4
HbA1c	Overall	rs72838857	G	A	0.213	0.144	0.019	3.4E-14	57.5
HbA1c	Overall	rs2854215	T	C	0.280	-0.113	0.018	1.4E-10	41.2
HbA1c	Overall	rs1030097	G	C	0.495	0.106	0.016	1.2E-11	45.9
HbA1c	Overall	rs1641523	T	C	0.567	-0.186	0.016	3.0E-32	139.8
HbA1c	Overall	rs2748427	G	A	0.218	0.540	0.019	2.9E-182	829.8
HbA1c	Overall	rs7208422	T	A	0.475	0.117	0.015	3.5E-14	57.4
HbA1c	Overall	rs113373052	T	C	0.313	0.467	0.017	3.5E-171	778.8
HbA1c	Overall	rs4791641	T	C	0.505	-0.148	0.016	1.3E-21	91.2
HbA1c	Overall	rs8088001	G	T	0.071	-0.168	0.030	2.8E-08	30.9
HbA1c	Overall	rs12607898	G	A	0.747	0.164	0.018	3.9E-20	84.5
HbA1c	Overall	rs12605978	T	C	0.322	0.093	0.017	2.5E-08	31.1
HbA1c	Overall	rs3810027	G	C	0.335	0.096	0.016	5.9E-09	33.9

HbA1c	Overall	rs2658747	G	A	0.379	0.110	0.016	7.0E-12	47.0
HbA1c	Overall	rs11671760	A	G	0.206	0.117	0.019	1.6E-09	36.4
HbA1c	Overall	rs56397034	C	G	0.391	-0.156	0.016	8.0E-23	96.7
HbA1c	Overall	rs4808579	C	T	0.392	0.315	0.016	2.0E-87	393.1
HbA1c	Overall	rs4808072	C	T	0.581	0.101	0.016	1.6E-10	40.9
HbA1c	Overall	rs75372982	G	A	0.289	-0.195	0.017	2.7E-29	126.3
HbA1c	Overall	rs72973028	C	A	0.172	0.135	0.021	7.0E-11	42.5
HbA1c	Overall	rs17561351	G	A	0.064	0.210	0.032	3.8E-11	43.7
HbA1c	Overall	rs10407429	A	G	0.426	-0.167	0.016	1.7E-26	113.5
HbA1c	Overall	rs62136856	G	A	0.717	0.127	0.017	1.8E-13	54.2
HbA1c	Overall	rs12459419	T	C	0.326	-0.172	0.017	2.1E-25	108.5
HbA1c	Overall	rs2278832	G	A	0.205	0.120	0.020	2.0E-09	36.0
HbA1c	Overall	rs45607834	T	C	0.103	0.154	0.026	2.0E-09	36.0
HbA1c	Overall	rs351978	G	A	0.581	-0.103	0.016	1.1E-10	41.7
HbA1c	Overall	rs11166447	C	T	0.359	-0.101	0.016	5.2E-10	38.6
HbA1c	Overall	rs76757826	C	T	0.055	0.206	0.034	1.6E-09	36.4
HbA1c	Overall	rs1975283	C	A	0.685	-0.166	0.017	4.1E-23	98.1
HbA1c	Overall	rs2487569	T	A	0.111	0.152	0.025	1.2E-09	36.9
HbA1c	Overall	rs198325	T	C	0.222	-0.184	0.019	5.6E-23	97.4
HbA1c	Overall	rs12748814	T	C	0.085	0.174	0.028	3.5E-10	39.4
HbA1c	Overall	rs857725	G	T	0.267	0.543	0.017	1.9E-211	964.4
HbA1c	Overall	rs7528296	C	T	0.386	-0.112	0.016	2.7E-12	48.9
HbA1c	Overall	rs523647	T	C	0.463	0.099	0.017	3.0E-09	35.2
HbA1c	Overall	rs2808454	T	A	0.542	0.145	0.016	1.8E-20	86.1
HbA1c	Overall	rs1078308	C	A	0.727	-0.124	0.017	1.4E-12	50.2
HbA1c	Overall	rs340882	G	C	0.621	0.239	0.016	5.7E-50	221.0
HbA1c	Overall	rs348330	A	G	0.635	-0.147	0.016	1.1E-19	82.4
HbA1c	Overall	rs3811444	T	C	0.334	0.166	0.016	5.7E-24	101.9
HbA1c	Overall	rs28695210	A	G	0.083	0.267	0.029	1.5E-20	86.4
HbA1c	Overall	rs563437995	C	G	0.008	-1.076	0.092	8.8E-32	137.7
HbA1c	Overall	rs797399	G	A	0.729	-0.097	0.018	4.0E-08	30.2
HbA1c	Overall	rs1175550	G	A	0.230	-0.285	0.019	1.6E-52	232.7
HbA1c	Overall	rs3768321	T	G	0.197	0.169	0.020	4.3E-18	75.2
HbA1c	Overall	rs557458132	T	C	0.282	-0.137	0.017	3.7E-15	61.9
HbA1c	Overall	rs72900998	C	T	0.089	-0.250	0.027	3.3E-20	84.8
HbA1c	Overall	rs4926930	G	A	0.426	-0.089	0.016	1.4E-08	32.2
HbA1c	Overall	rs7513688	A	G	0.361	0.126	0.016	6.1E-15	60.9
HbA1c	Overall	rs1166698	A	G	0.212	-0.122	0.019	1.2E-10	41.5
HbA1c	Overall	rs13042195	G	A	0.213	-0.108	0.019	1.6E-08	32.0
HbA1c	Overall	rs149142833	T	C	0.156	0.172	0.022	1.7E-15	63.3
HbA1c	Overall	rs4431018	G	A	0.379	0.088	0.016	4.3E-08	30.0
HbA1c	Overall	rs67897819	A	G	0.212	0.129	0.020	5.0E-11	43.2
HbA1c	Overall	rs55966194	G	C	0.284	-0.149	0.017	6.0E-18	74.5
HbA1c	Overall	rs6014993	G	A	0.487	0.176	0.016	1.6E-29	127.3
HbA1c	Overall	rs4809556	A	G	0.596	-0.106	0.016	2.3E-11	44.7
HbA1c	Overall	rs1056441	C	T	0.673	0.099	0.017	2.0E-09	35.9
HbA1c	Overall	rs6077396	A	G	0.507	-0.142	0.016	7.9E-20	83.1
HbA1c	Overall	rs3827181	T	C	0.346	0.127	0.016	6.2E-15	60.8

HbA1c	Overall	rs61138219	T	C	0.153	-0.155	0.022	7.6E-13	51.4
HbA1c	Overall	rs17850433	C	T	0.012	0.633	0.071	6.4E-19	79.0
HbA1c	Overall	rs35963032	T	C	0.152	-0.140	0.022	1.4E-10	41.1
HbA1c	Overall	rs12628032	T	C	0.305	0.103	0.017	8.1E-10	37.7
HbA1c	Overall	rs6518681	G	A	0.907	0.220	0.027	2.5E-16	67.2
HbA1c	Overall	rs117721418	T	C	0.081	-0.226	0.028	1.9E-15	63.1
HbA1c	Overall	rs855791	G	A	0.561	-0.422	0.016	4.1E-159	723.2
HbA1c	Overall	rs2143918	C	A	0.471	-0.186	0.016	5.3E-33	143.3
HbA1c	Overall	rs2009581	A	G	0.275	0.096	0.017	4.5E-08	29.9
HbA1c	Overall	rs650588	G	A	0.581	-0.106	0.016	1.7E-11	45.3
HbA1c	Overall	rs111631066	A	G	0.015	-0.479	0.065	1.6E-13	54.5
HbA1c	Overall	rs4667575	G	C	0.592	0.088	0.016	2.6E-08	31.0
HbA1c	Overall	rs3843328	A	G	0.711	-0.161	0.017	1.2E-20	86.9
HbA1c	Overall	rs853787	T	G	0.651	0.636	0.016	0.0E+00	1517.2
HbA1c	Overall	rs4972439	C	T	0.220	-0.193	0.019	8.9E-25	105.7
HbA1c	Overall	rs6731171	A	T	0.626	0.104	0.016	1.9E-10	40.5
HbA1c	Overall	rs6736362	T	C	0.562	0.114	0.016	4.3E-13	52.5
HbA1c	Overall	rs1399627	G	A	0.653	0.153	0.016	6.3E-21	88.1
HbA1c	Overall	rs838717	A	G	0.566	0.114	0.016	3.0E-13	53.2
HbA1c	Overall	rs72781680	T	C	0.135	0.348	0.023	1.2E-52	233.2
HbA1c	Overall	rs13020237	G	A	0.182	-0.114	0.020	1.2E-08	32.5
HbA1c	Overall	rs780093	C	T	0.618	0.177	0.016	1.4E-28	123.0
HbA1c	Overall	rs11124556	T	G	0.514	-0.090	0.016	6.7E-09	33.6
HbA1c	Overall	rs77981966	T	C	0.076	-0.490	0.029	6.3E-63	280.5
HbA1c	Overall	rs2164701	C	G	0.140	0.199	0.022	7.1E-19	78.8
HbA1c	Overall	rs78583604	T	G	0.132	0.180	0.024	2.6E-14	58.0
HbA1c	Overall	rs7606173	C	G	0.431	-0.164	0.016	1.2E-25	109.6
HbA1c	Overall	rs7572278	A	T	0.204	0.194	0.019	8.2E-24	101.2
HbA1c	Overall	rs4435501	C	A	0.108	0.141	0.026	4.3E-08	30.0
HbA1c	Overall	rs9826367	G	A	0.449	-0.251	0.016	2.7E-58	259.2
HbA1c	Overall	rs11708067	G	A	0.245	-0.353	0.018	1.6E-85	384.3
HbA1c	Overall	rs12497133	A	G	0.533	0.120	0.016	2.1E-14	58.4
HbA1c	Overall	rs13059110	T	G	0.133	0.144	0.023	3.6E-10	39.3
HbA1c	Overall	rs75367758	A	G	0.048	-0.296	0.036	3.2E-16	66.7
HbA1c	Overall	rs12493136	A	G	0.164	0.169	0.021	1.0E-15	64.4
HbA1c	Overall	rs11926854	C	T	0.037	-0.240	0.042	7.9E-09	33.3
HbA1c	Overall	rs6785881	T	C	0.481	-0.140	0.016	2.2E-19	81.1
HbA1c	Overall	rs4955744	C	A	0.146	0.134	0.022	1.0E-09	37.3
HbA1c	Overall	rs1905505	A	G	0.284	-0.283	0.017	1.2E-60	270.0
HbA1c	Overall	rs360408	A	T	0.565	-0.124	0.016	9.1E-15	60.1
HbA1c	Overall	rs4894797	A	G	0.576	0.188	0.016	4.5E-33	143.6
HbA1c	Overall	rs4859137	T	C	0.772	0.117	0.019	3.4E-10	39.4
HbA1c	Overall	rs73061095	G	T	0.314	0.147	0.017	1.3E-18	77.6
HbA1c	Overall	rs6777684	G	A	0.610	0.175	0.016	7.2E-28	119.8
HbA1c	Overall	rs41297495	T	C	0.382	-0.099	0.016	4.9E-10	38.7
HbA1c	Overall	rs2015708	T	C	0.137	0.167	0.023	1.5E-13	54.5
HbA1c	Overall	rs2686588	G	C	0.565	-0.135	0.016	6.0E-18	74.5
HbA1c	Overall	rs1496653	G	A	0.204	-0.138	0.019	6.8E-13	51.6

HbA1c	Overall	rs4678917	C	T	0.105	-0.146	0.026	1.3E-08	32.4
HbA1c	Overall	rs62246446	G	C	0.176	-0.138	0.020	1.3E-11	45.9
HbA1c	Overall	rs2230929	A	G	0.171	0.258	0.021	8.8E-36	156.0
HbA1c	Overall	rs2336664	G	A	0.363	0.097	0.016	2.0E-09	36.0
HbA1c	Overall	rs11130982	G	T	0.711	0.110	0.017	1.6E-10	40.9
HbA1c	Overall	rs9840088	C	A	0.201	-0.113	0.020	7.9E-09	33.3
HbA1c	Overall	rs397834892	G	A	0.471	-0.105	0.016	1.6E-11	45.4
HbA1c	Overall	rs17508261	C	T	0.122	0.188	0.024	2.5E-15	62.6
HbA1c	Overall	rs9994509	G	A	0.393	-0.135	0.016	2.8E-17	71.5
HbA1c	Overall	rs2044341	C	T	0.684	-0.356	0.017	5.9E-100	450.7
HbA1c	Overall	rs76505278	C	T	0.103	-0.187	0.026	2.7E-13	53.5
HbA1c	Overall	rs13125518	G	A	0.459	-0.090	0.016	1.1E-08	32.7
HbA1c	Overall	rs75636043	A	G	0.078	-0.172	0.029	3.9E-09	34.7
HbA1c	Overall	rs6840504	T	C	0.443	0.113	0.016	4.9E-13	52.3
HbA1c	Overall	rs55881843	A	G	0.139	-0.226	0.023	1.5E-23	100.0
HbA1c	Overall	rs34811474	A	G	0.232	-0.107	0.018	5.0E-09	34.2
HbA1c	Overall	rs6844176	C	T	0.455	0.087	0.016	2.8E-08	30.9
HbA1c	Overall	rs9990955	T	C	0.284	0.126	0.017	2.1E-13	53.9
HbA1c	Overall	rs4689394	G	C	0.599	0.121	0.016	2.9E-14	57.8
HbA1c	Overall	rs1481012	G	A	0.113	0.198	0.025	6.9E-16	65.2
HbA1c	Overall	rs35188965	T	C	0.582	0.120	0.016	2.6E-14	58.0
HbA1c	Overall	rs469753	T	G	0.475	0.091	0.016	5.3E-09	34.1
HbA1c	Overall	rs10053046	G	A	0.329	0.118	0.017	1.5E-12	50.1
HbA1c	Overall	rs6885132	G	C	0.099	-0.233	0.026	8.5E-19	78.4
HbA1c	Overall	rs35018839	G	A	0.095	-0.151	0.027	1.9E-08	31.6
HbA1c	Overall	rs13167071	C	G	0.640	0.135	0.016	5.9E-17	70.0
HbA1c	Overall	rs7720275	C	T	0.170	0.161	0.021	6.2E-15	60.8
HbA1c	Overall	rs56395926	A	G	0.229	-0.108	0.019	5.2E-09	34.1
HbA1c	Overall	rs3857286	T	C	0.695	-0.104	0.017	9.4E-10	37.5
HbA1c	Overall	rs6893283	T	C	0.015	-0.377	0.065	6.0E-09	33.8
HbA1c	Overall	rs145381182	A	G	0.090	0.157	0.027	1.0E-08	32.8
HbA1c	Overall	rs2270927	G	C	0.094	0.193	0.027	4.1E-13	52.6
HbA1c	Overall	rs6878122	A	G	0.680	-0.181	0.017	1.5E-27	118.3
HbA1c	Overall	rs17419291	C	T	0.087	-0.165	0.028	1.9E-09	36.1
HbA1c	Overall	rs2546197	G	T	0.078	0.160	0.029	3.6E-08	30.3
HbA1c	Overall	rs144489757	G	C	0.284	-0.120	0.017	3.0E-12	48.7
HbA1c	Overall	rs148570357	T	C	0.295	-0.155	0.017	1.6E-19	81.7
HbA1c	Overall	rs72939920	T	A	0.235	-0.194	0.018	4.6E-26	111.5
HbA1c	Overall	rs9398642	T	C	0.145	-0.132	0.022	2.2E-09	35.8
HbA1c	Overall	rs1490384	T	C	0.498	0.122	0.016	4.8E-15	61.3
HbA1c	Overall	rs9402645	T	G	0.200	0.119	0.020	1.1E-09	37.1
HbA1c	Overall	rs9376091	T	C	0.262	-0.288	0.018	4.3E-59	262.9
HbA1c	Overall	rs592423	C	A	0.554	-0.131	0.016	3.6E-17	71.0
HbA1c	Overall	rs3910736	T	C	0.308	0.110	0.017	7.1E-11	42.5
HbA1c	Overall	rs262813	C	T	0.478	0.090	0.016	8.0E-09	33.3
HbA1c	Overall	rs2665357	C	A	0.517	0.120	0.016	1.5E-14	59.2
HbA1c	Overall	rs67131976	T	C	0.172	0.315	0.021	3.2E-53	235.9
HbA1c	Overall	rs142513102	G	A	0.026	-0.484	0.049	1.9E-23	99.6

HbA1c	Overall	rs1800562	A	G	0.079	-0.842	0.029	6.8E-188	855.8
HbA1c	Overall	rs2442725	C	T	0.694	0.159	0.017	4.3E-21	88.8
HbA1c	Overall	rs9268469	A	G	0.155	0.301	0.021	1.2E-44	196.5
HbA1c	Overall	rs833058	T	C	0.365	0.111	0.016	5.2E-12	47.6
HbA1c	Overall	rs998584	A	C	0.482	0.156	0.016	1.2E-23	100.6
HbA1c	Overall	rs2294856	C	G	0.355	0.114	0.016	1.9E-12	49.6
HbA1c	Overall	rs3748059	G	A	0.799	-0.149	0.019	1.6E-14	58.9
HbA1c	Overall	rs13192435	C	T	0.025	0.353	0.049	8.5E-13	51.2
HbA1c	Overall	rs78588343	A	G	0.179	-0.226	0.020	8.3E-29	124.0
HbA1c	Overall	rs1171105	C	T	0.695	0.095	0.017	4.1E-08	30.1
HbA1c	Overall	rs12667932	A	G	0.080	-0.314	0.029	5.5E-28	120.3
HbA1c	Overall	rs35722851	C	T	0.495	-0.088	0.016	1.5E-08	32.1
HbA1c	Overall	rs8176059	A	G	0.011	-0.439	0.073	2.2E-09	35.8
HbA1c	Overall	rs17168486	T	C	0.173	0.198	0.021	7.4E-22	92.3
HbA1c	Overall	rs10487796	A	T	0.450	-0.183	0.016	9.0E-32	137.6
HbA1c	Overall	rs6459737	A	G	0.347	-0.117	0.016	6.4E-13	51.7
HbA1c	Overall	rs60238952	G	A	0.219	-0.113	0.019	2.3E-09	35.7
HbA1c	Overall	rs1708302	T	C	0.501	-0.178	0.016	1.4E-30	132.2
HbA1c	Overall	rs76323047	G	A	0.118	0.335	0.024	5.4E-44	193.6
HbA1c	Overall	rs1004558	T	C	0.179	0.727	0.020	4.1E-283	1295.3
HbA1c	Overall	rs836472	A	G	0.266	-0.109	0.018	4.7E-10	38.8
HbA1c	Overall	rs2460421	T	A	0.502	-0.112	0.015	5.8E-13	51.9
HbA1c	Overall	rs6953344	G	A	0.246	0.127	0.018	2.2E-12	49.3
HbA1c	Overall	rs3211821	G	A	0.562	-0.106	0.016	1.7E-11	45.3
HbA1c	Overall	rs6973494	A	C	0.516	0.116	0.016	1.4E-13	54.7
HbA1c	Overall	rs10488532	T	C	0.108	-0.143	0.025	1.1E-08	32.6
HbA1c	Overall	rs117370443	C	T	0.039	0.373	0.040	1.4E-20	86.6
HbA1c	Overall	rs636089	C	T	0.386	0.095	0.016	2.9E-09	35.3
HbA1c	Overall	rs7820334	T	C	0.311	-0.146	0.017	4.8E-18	75.0
HbA1c	Overall	rs2737263	T	G	0.284	-0.156	0.017	1.1E-19	82.4
HbA1c	Overall	rs13266634	T	C	0.311	-0.379	0.017	2.4E-113	512.3
HbA1c	Overall	rs58253018	T	A	0.635	0.105	0.017	2.3E-10	40.2
HbA1c	Overall	rs2060985	G	A	0.537	-0.122	0.016	4.8E-15	61.3
HbA1c	Overall	rs3757971	C	T	0.380	0.130	0.016	3.2E-16	66.7
HbA1c	Overall	rs187055391	A	G	0.172	-0.134	0.021	1.5E-10	41.0
HbA1c	Overall	rs66593272	T	A	0.038	-0.915	0.041	2.4E-111	503.0
HbA1c	Overall	rs4737010	A	G	0.228	0.534	0.019	1.0E-182	831.9
HbA1c	Overall	rs55840085	A	G	0.355	0.169	0.016	1.7E-25	108.9
HbA1c	Overall	rs28708563	A	G	0.042	0.220	0.039	2.3E-08	31.3
HbA1c	Overall	rs62523081	A	G	0.362	0.094	0.016	7.1E-09	33.5
HbA1c	Overall	rs1967604	G	A	0.712	-0.208	0.017	1.1E-33	146.3
HbA1c	Overall	rs1560980	C	G	0.041	0.316	0.039	5.1E-16	65.8
HbA1c	Overall	rs584048	T	C	0.861	0.134	0.022	2.1E-09	35.9
HbA1c	Overall	rs5900831	G	T	0.653	0.135	0.017	3.2E-16	66.7
HbA1c	Overall	rs550057	T	C	0.255	0.325	0.018	5.9E-74	331.1
HbA1c	Overall	rs28641468	C	T	0.756	0.234	0.018	1.9E-38	168.2
HbA1c	Overall	rs10811660	A	G	0.173	-0.359	0.020	1.5E-68	306.3
HbA1c	Overall	rs10758593	A	G	0.397	0.107	0.016	1.1E-11	46.1

HbA1c	Overall	rs2236496	C	T	0.208	-0.182	0.019	2.5E-21	89.9
HbA1c	Overall	rs11145347	C	T	0.197	0.239	0.020	1.7E-34	150.1
HbA1c	Overall	rs61750929	T	C	0.055	-0.692	0.034	8.6E-92	413.1
HbA1c	Overall	rs7047279	C	T	0.568	-0.124	0.016	4.2E-15	61.6
HbA1c	Women	rs7903146	T	C	0.290	0.423	0.023	5.3E-76	340.7
HbA1c	Women	rs11257655	T	C	0.208	0.234	0.026	7.3E-20	83.2
HbA1c	Women	rs7923442	G	A	0.178	-0.156	0.027	9.8E-09	32.9
HbA1c	Women	rs61850681	A	G	0.239	-0.218	0.025	5.9E-19	79.1
HbA1c	Women	rs10998678	A	G	0.053	0.341	0.047	2.6E-13	53.5
HbA1c	Women	rs2015803	T	C	0.247	0.318	0.024	1.4E-39	173.3
HbA1c	Women	rs17476364	C	T	0.110	-2.079	0.033	0.0E+00	3990.7
HbA1c	Women	rs2305196	A	G	0.265	-0.221	0.024	1.1E-20	86.9
HbA1c	Women	rs7077479	A	C	0.337	0.140	0.022	3.9E-10	39.2
HbA1c	Women	rs701848	C	T	0.390	-0.118	0.021	3.2E-08	30.6
HbA1c	Women	rs11187149	A	G	0.381	-0.189	0.021	9.1E-19	78.3
HbA1c	Women	rs4418728	T	G	0.449	-0.152	0.021	3.5E-13	52.9
HbA1c	Women	rs12365580	A	G	0.151	-0.209	0.029	7.6E-13	51.4
HbA1c	Women	rs11042850	A	G	0.087	0.292	0.037	2.0E-15	63.1
HbA1c	Women	rs7944748	G	T	0.378	0.119	0.022	3.2E-08	30.6
HbA1c	Women	rs2351960	G	A	0.893	-0.219	0.034	7.8E-11	42.3
HbA1c	Women	rs3782123	A	C	0.733	-0.203	0.024	6.9E-18	74.3
HbA1c	Women	rs11564725	T	C	0.236	0.202	0.025	3.1E-16	66.7
HbA1c	Women	rs4755950	T	C	0.113	-0.187	0.033	1.2E-08	32.5
HbA1c	Women	rs10501320	C	G	0.264	-0.194	0.024	1.8E-16	67.8
HbA1c	Women	rs174598	A	G	0.329	-0.148	0.022	2.4E-11	44.6
HbA1c	Women	rs12294913	G	C	0.050	0.299	0.050	1.6E-09	36.4
HbA1c	Women	rs11602873	T	A	0.157	-0.224	0.029	4.3E-15	61.6
HbA1c	Women	rs10830963	G	C	0.275	0.434	0.023	1.7E-77	347.5
HbA1c	Women	rs1392026	T	C	0.635	-0.175	0.022	7.3E-16	65.1
HbA1c	Women	rs7310615	G	C	0.517	0.188	0.021	2.9E-19	80.5
HbA1c	Women	rs2936839	C	T	0.610	-0.129	0.021	1.6E-09	36.4
HbA1c	Women	rs147730268	T	G	0.091	-0.229	0.037	9.2E-10	37.5
HbA1c	Women	rs10842994	T	C	0.196	-0.156	0.026	2.4E-09	35.7
HbA1c	Women	rs76895963	G	T	0.020	-1.082	0.080	1.1E-41	183.1
HbA1c	Women	rs10492081	G	A	0.180	0.198	0.027	2.4E-13	53.7
HbA1c	Women	rs4760682	A	C	0.813	0.501	0.027	5.8E-79	354.3
HbA1c	Women	rs35979828	T	C	0.069	-0.236	0.041	8.9E-09	33.1
HbA1c	Women	rs2261181	T	C	0.096	0.244	0.035	5.8E-12	47.4
HbA1c	Women	rs1579238	G	A	0.241	-0.157	0.024	1.1E-10	41.6
HbA1c	Women	rs34011672	G	A	0.089	0.562	0.037	4.0E-53	235.5
HbA1c	Women	rs12861645	G	T	0.272	0.241	0.023	8.1E-25	105.8
HbA1c	Women	rs11616945	C	T	0.337	0.140	0.022	4.0E-10	39.1
HbA1c	Women	rs12020979	G	T	0.185	-0.268	0.028	6.3E-22	92.7
HbA1c	Women	rs4325403	A	T	0.220	0.231	0.025	1.0E-19	82.6
HbA1c	Women	rs75629141	A	T	0.829	-0.164	0.028	3.0E-09	35.2
HbA1c	Women	rs941899	C	T	0.529	-0.135	0.021	8.7E-11	42.1
HbA1c	Women	rs9324022	C	T	0.206	0.163	0.026	5.6E-10	38.5
HbA1c	Women	rs1760940	C	A	0.248	0.176	0.024	2.4E-13	53.6

HbA1c	Women	rs941718	C	T	0.278	0.151	0.023	7.3E-11	42.4
HbA1c	Women	rs8018574	A	T	0.243	-0.154	0.024	2.4E-10	40.1
HbA1c	Women	rs12878001	G	T	0.160	0.160	0.029	2.1E-08	31.4
HbA1c	Women	rs229572	A	T	0.235	0.190	0.025	8.5E-15	60.2
HbA1c	Women	rs35889227	T	G	0.631	-0.143	0.022	3.3E-11	44.0
HbA1c	Women	rs4516170	C	T	0.050	-0.313	0.048	5.8E-11	42.9
HbA1c	Women	rs1550026	T	A	0.408	0.137	0.021	1.1E-10	41.6
HbA1c	Women	rs869301	C	T	0.720	0.192	0.023	1.2E-16	68.6
HbA1c	Women	rs11638511	G	A	0.561	0.126	0.021	2.2E-09	35.8
HbA1c	Women	rs4451969	C	T	0.671	-0.150	0.022	1.5E-11	45.6
HbA1c	Women	rs9302377	T	C	0.493	0.123	0.022	2.5E-08	31.1
HbA1c	Women	rs6600191	C	T	0.181	-0.218	0.027	9.5E-16	64.5
HbA1c	Women	rs35661204	T	C	0.226	0.189	0.025	3.2E-14	57.6
HbA1c	Women	rs8050500	C	T	0.446	-0.162	0.021	1.1E-14	59.8
HbA1c	Women	rs11075985	A	C	0.423	0.124	0.021	3.8E-09	34.7
HbA1c	Women	rs9929239	T	C	0.292	0.159	0.023	3.0E-12	48.7
HbA1c	Women	rs247828	C	A	0.305	0.138	0.023	9.8E-10	37.4
HbA1c	Women	rs72811487	A	G	0.035	-0.413	0.056	2.0E-13	54.1
HbA1c	Women	rs551118	G	C	0.578	0.372	0.021	1.1E-67	302.4
HbA1c	Women	rs11655029	C	T	0.695	-0.211	0.023	1.1E-20	87.0
HbA1c	Women	rs6902	C	A	0.386	-0.118	0.021	3.4E-08	30.5
HbA1c	Women	rs12450826	T	A	0.293	-0.172	0.023	2.4E-13	53.7
HbA1c	Women	rs11651755	T	C	0.515	-0.133	0.021	1.7E-10	40.8
HbA1c	Women	rs2060779	A	G	0.222	-0.176	0.025	2.3E-12	49.3
HbA1c	Women	rs9899611	G	A	0.213	0.163	0.025	1.3E-10	41.3
HbA1c	Women	rs858518	A	G	0.573	-0.144	0.021	7.6E-12	46.9
HbA1c	Women	rs2748427	G	A	0.218	0.637	0.025	5.2E-142	644.8
HbA1c	Women	rs112281307	C	A	0.094	0.229	0.036	1.2E-10	41.5
HbA1c	Women	rs9909940	T	C	0.312	0.474	0.022	3.2E-99	447.6
HbA1c	Women	rs4792590	G	A	0.877	-0.215	0.032	1.2E-11	45.9
HbA1c	Women	rs4542747	T	C	0.746	0.153	0.024	1.7E-10	40.8
HbA1c	Women	rs8110787	T	C	0.391	-0.212	0.021	2.0E-23	99.4
HbA1c	Women	rs4808579	C	T	0.392	0.300	0.021	7.7E-45	197.5
HbA1c	Women	rs4499344	A	G	0.293	-0.211	0.023	2.7E-20	85.2
HbA1c	Women	rs10410896	A	C	0.890	0.199	0.034	3.7E-09	34.8
HbA1c	Women	rs55914970	T	G	0.441	-0.154	0.021	2.2E-13	53.8
HbA1c	Women	rs12459419	T	C	0.326	-0.163	0.022	2.0E-13	54.1
HbA1c	Women	rs7250849	T	G	0.106	0.235	0.034	3.8E-12	48.2
HbA1c	Women	rs351988	G	A	0.565	-0.117	0.021	3.3E-08	30.5
HbA1c	Women	rs1975283	C	A	0.685	-0.169	0.023	6.6E-14	56.2
HbA1c	Women	rs267738	G	T	0.221	-0.201	0.025	9.0E-16	64.7
HbA1c	Women	rs4133213	A	C	0.450	-0.121	0.021	1.5E-08	32.1
HbA1c	Women	rs857721	A	T	0.267	0.504	0.023	1.8E-102	462.6
HbA1c	Women	rs7528296	C	T	0.386	-0.130	0.021	1.1E-09	37.1
HbA1c	Women	rs1044145	C	T	0.540	0.155	0.021	1.3E-13	54.8
HbA1c	Women	rs340882	G	C	0.621	0.254	0.022	4.8E-32	138.9
HbA1c	Women	rs348330	A	G	0.635	-0.131	0.022	1.5E-09	36.5
HbA1c	Women	rs1339847	A	G	0.110	-0.224	0.033	1.7E-11	45.3

HbA1c	Women	rs2301153	C	G	0.799	-0.171	0.028	4.9E-10	38.7
HbA1c	Women	rs563437995	C	G	0.008	-1.019	0.123	1.1E-16	68.8
HbA1c	Women	rs1175550	G	A	0.230	-0.268	0.025	1.1E-26	114.4
HbA1c	Women	rs12141885	T	C	0.700	-0.126	0.023	2.6E-08	31.0
HbA1c	Women	rs1768809	C	A	0.556	0.132	0.021	3.0E-10	39.7
HbA1c	Women	rs12121576	G	A	0.183	-0.163	0.028	4.9E-09	34.2
HbA1c	Women	rs149142833	T	C	0.156	0.165	0.029	1.2E-08	32.5
HbA1c	Women	rs6066148	C	G	0.262	-0.144	0.024	1.3E-09	36.8
HbA1c	Women	rs6014993	G	A	0.487	0.204	0.021	1.5E-22	95.6
HbA1c	Women	rs73129526	C	T	0.144	0.168	0.030	1.4E-08	32.2
HbA1c	Women	rs6077396	A	G	0.507	-0.114	0.021	4.4E-08	30.0
HbA1c	Women	rs3827181	T	C	0.346	0.131	0.022	2.3E-09	35.7
HbA1c	Women	rs61138219	T	C	0.153	-0.160	0.029	3.3E-08	30.5
HbA1c	Women	rs17850433	C	T	0.012	0.624	0.095	4.7E-11	43.3
HbA1c	Women	rs62221525	T	C	0.179	-0.168	0.027	7.1E-10	38.0
HbA1c	Women	rs4680	A	G	0.516	0.119	0.021	9.4E-09	33.0
HbA1c	Women	rs6518681	G	A	0.907	0.206	0.036	1.0E-08	32.8
HbA1c	Women	rs855791	G	A	0.561	-0.417	0.021	1.3E-87	394.1
HbA1c	Women	rs2143918	C	A	0.471	-0.166	0.021	1.9E-15	63.1
HbA1c	Women	rs111631066	A	G	0.015	-0.550	0.087	2.8E-10	39.8
HbA1c	Women	rs180935712	A	G	0.019	0.533	0.078	9.8E-12	46.4
HbA1c	Women	rs143869345	G	A	0.017	0.681	0.082	1.1E-16	68.9
HbA1c	Women	rs560887	C	T	0.701	0.738	0.023	1.2E-232	1063.7
HbA1c	Women	rs4972439	C	T	0.220	-0.192	0.025	2.6E-14	58.0
HbA1c	Women	rs1047891	A	C	0.316	-0.154	0.022	5.6E-12	47.5
HbA1c	Women	rs13427681	C	G	0.562	0.129	0.021	7.0E-10	38.0
HbA1c	Women	rs2943640	C	A	0.652	0.121	0.022	2.7E-08	30.9
HbA1c	Women	rs7346	A	G	0.191	-0.163	0.027	7.7E-10	37.8
HbA1c	Women	rs72780125	C	T	0.131	0.429	0.031	8.3E-44	192.8
HbA1c	Women	rs7595986	A	G	0.781	-0.156	0.025	5.1E-10	38.7
HbA1c	Women	rs1504	G	T	0.444	-0.116	0.021	2.9E-08	30.8
HbA1c	Women	rs112694524	A	G	0.072	-0.469	0.041	2.0E-30	131.4
HbA1c	Women	rs2121564	G	C	0.129	0.169	0.031	4.8E-08	29.8
HbA1c	Women	rs78583604	T	G	0.132	0.175	0.032	3.1E-08	30.6
HbA1c	Women	rs13019832	A	G	0.418	-0.149	0.021	2.2E-12	49.3
HbA1c	Women	rs7572278	A	T	0.204	0.175	0.026	1.2E-11	45.9
HbA1c	Women	rs9826367	G	A	0.449	-0.258	0.021	5.2E-35	152.4
HbA1c	Women	rs11708067	G	A	0.245	-0.392	0.024	2.8E-59	263.8
HbA1c	Women	rs6807945	T	C	0.837	-0.186	0.028	5.7E-11	42.9
HbA1c	Women	rs77630894	T	G	0.049	-0.266	0.048	3.8E-08	30.3
HbA1c	Women	rs12493136	A	G	0.164	0.200	0.028	1.3E-12	50.3
HbA1c	Women	rs16831139	G	A	0.400	-0.137	0.022	2.5E-10	40.0
HbA1c	Women	rs5398	A	G	0.285	-0.284	0.023	4.6E-35	152.7
HbA1c	Women	rs360408	A	T	0.565	-0.123	0.021	9.8E-09	32.9
HbA1c	Women	rs16856859	G	A	0.583	0.199	0.021	5.0E-21	88.5
HbA1c	Women	rs10222374	T	C	0.785	0.152	0.026	2.6E-09	35.4
HbA1c	Women	rs6777684	G	A	0.610	0.173	0.021	5.4E-16	65.6
HbA1c	Women	rs2686588	G	C	0.565	-0.133	0.021	2.2E-10	40.3

HbA1c	Women	rs62246446	G	C	0.176	-0.166	0.027	1.1E-09	37.1
HbA1c	Women	rs67882627	T	A	0.171	0.251	0.028	1.1E-19	82.4
HbA1c	Women	rs11130982	G	T	0.711	0.178	0.023	1.3E-14	59.5
HbA1c	Women	rs223376	A	T	0.485	-0.122	0.021	5.6E-09	34.0
HbA1c	Women	rs17430251	G	A	0.122	0.178	0.032	2.1E-08	31.4
HbA1c	Women	rs2298978	A	G	0.403	-0.142	0.021	2.5E-11	44.6
HbA1c	Women	rs6814613	C	G	0.665	-0.364	0.023	1.1E-58	261.0
HbA1c	Women	rs34835465	C	T	0.070	-0.295	0.041	5.8E-13	51.9
HbA1c	Women	rs6840504	T	C	0.443	0.127	0.021	1.5E-09	36.5
HbA1c	Women	rs11132255	A	G	0.161	-0.237	0.028	5.9E-17	70.0
HbA1c	Women	rs11731874	C	T	0.575	0.123	0.021	6.5E-09	33.7
HbA1c	Women	rs2622622	G	C	0.314	-0.158	0.023	2.9E-12	48.8
HbA1c	Women	rs112034864	G	C	0.503	0.141	0.021	1.9E-11	45.1
HbA1c	Women	rs6885132	G	C	0.099	-0.252	0.035	9.5E-13	51.0
HbA1c	Women	rs4704834	G	A	0.646	0.181	0.022	1.1E-16	68.9
HbA1c	Women	rs4496693	C	T	0.094	0.219	0.036	9.1E-10	37.5
HbA1c	Women	rs17419291	C	T	0.087	-0.211	0.037	1.0E-08	32.8
HbA1c	Women	rs35247507	G	A	0.286	-0.164	0.023	1.3E-12	50.4
HbA1c	Women	rs13214717	A	C	0.299	-0.165	0.023	3.7E-13	52.8
HbA1c	Women	rs72939920	T	A	0.235	-0.205	0.025	7.1E-17	69.7
HbA1c	Women	rs9385400	G	T	0.455	0.160	0.021	1.9E-14	58.6
HbA1c	Women	rs9376091	T	C	0.262	-0.274	0.024	1.0E-30	132.8
HbA1c	Women	rs592423	C	A	0.554	-0.114	0.021	4.6E-08	29.9
HbA1c	Women	rs6920313	C	T	0.306	0.131	0.023	6.4E-09	33.7
HbA1c	Women	rs9368219	T	C	0.173	0.282	0.028	1.6E-24	104.6
HbA1c	Women	rs1800562	A	G	0.079	-0.871	0.039	7.0E-113	510.5
HbA1c	Women	rs2071596	A	G	0.163	-0.232	0.028	1.4E-16	68.2
HbA1c	Women	rs11967891	T	A	0.181	0.320	0.028	3.7E-30	130.3
HbA1c	Women	rs9272606	T	C	0.660	0.127	0.022	8.6E-09	33.1
HbA1c	Women	rs998584	A	C	0.482	0.191	0.021	4.9E-20	84.0
HbA1c	Women	rs34101263	A	G	0.299	0.148	0.023	6.6E-11	42.6
HbA1c	Women	rs3748059	G	A	0.799	-0.168	0.026	9.6E-11	41.9
HbA1c	Women	rs78588343	A	G	0.179	-0.268	0.027	5.1E-23	97.6
HbA1c	Women	rs75759008	G	A	0.080	-0.284	0.038	1.2E-13	55.1
HbA1c	Women	rs12154627	C	T	0.498	-0.120	0.021	1.3E-08	32.3
HbA1c	Women	rs17168486	T	C	0.173	0.190	0.028	6.7E-12	47.1
HbA1c	Women	rs1974620	T	C	0.549	0.153	0.021	3.4E-13	53.0
HbA1c	Women	rs62491945	A	G	0.347	-0.125	0.022	8.7E-09	33.1
HbA1c	Women	rs864745	C	T	0.506	-0.170	0.021	2.6E-16	67.1
HbA1c	Women	rs2908277	A	G	0.120	0.333	0.032	2.2E-25	108.4
HbA1c	Women	rs1799884	T	C	0.178	0.769	0.027	2.4E-177	807.9
HbA1c	Women	rs868961	T	G	0.340	0.132	0.022	1.8E-09	36.2
HbA1c	Women	rs6973494	A	C	0.516	0.120	0.021	1.3E-08	32.3
HbA1c	Women	rs117370443	C	T	0.039	0.335	0.054	4.7E-10	38.8
HbA1c	Women	rs7820334	T	C	0.311	-0.143	0.023	2.6E-10	40.0
HbA1c	Women	rs55780214	A	T	0.290	-0.192	0.023	7.1E-17	69.7
HbA1c	Women	rs13266634	T	C	0.311	-0.369	0.022	1.3E-60	269.9
HbA1c	Women	rs2060984	C	T	0.450	-0.119	0.021	1.2E-08	32.5

HbA1c	Women	rs6474359	C	T	0.038	-0.854	0.054	2.3E-55	245.8
HbA1c	Women	rs4737010	A	G	0.228	0.471	0.025	1.8E-80	361.3
HbA1c	Women	rs55840085	A	G	0.355	0.157	0.022	4.8E-13	52.3
HbA1c	Women	rs7042939	G	A	0.620	-0.199	0.022	3.8E-20	84.5
HbA1c	Women	rs41273438	G	A	0.041	0.322	0.052	7.9E-10	37.8
HbA1c	Women	rs5900831	G	T	0.653	0.142	0.022	1.5E-10	41.0
HbA1c	Women	rs550057	T	C	0.255	0.287	0.024	3.8E-33	143.9
HbA1c	Women	rs28641468	C	T	0.756	0.256	0.024	2.7E-26	112.6
HbA1c	Women	rs10811660	A	G	0.173	-0.334	0.027	4.7E-34	148.1
HbA1c	Women	rs2236495	C	T	0.208	-0.192	0.026	7.9E-14	55.8
HbA1c	Women	rs11145347	C	T	0.197	0.221	0.026	2.7E-17	71.6
HbA1c	Women	rs61750929	T	C	0.055	-0.716	0.046	2.5E-55	245.7
HbA1c	Men	rs114322470	G	T	0.019	-0.565	0.086	5.1E-11	43.1
HbA1c	Men	rs34872471	C	T	0.291	0.555	0.026	1.2E-104	472.6
HbA1c	Men	rs11257655	T	C	0.208	0.256	0.029	3.0E-19	80.5
HbA1c	Men	rs7904973	T	G	0.576	0.135	0.023	1.0E-08	32.8
HbA1c	Men	rs3780962	G	A	0.532	0.135	0.023	7.4E-09	33.4
HbA1c	Men	rs56197478	G	A	0.223	-0.256	0.028	9.5E-20	82.7
HbA1c	Men	rs550048289	G	T	0.015	-0.830	0.102	3.9E-16	66.3
HbA1c	Men	rs1106676	G	T	0.656	0.290	0.024	2.0E-32	140.6
HbA1c	Men	rs11812380	C	T	0.012	0.757	0.111	7.9E-12	46.8
HbA1c	Men	rs10159477	A	G	0.149	-1.770	0.032	0.0E+00	2999.4
HbA1c	Men	rs749108	T	C	0.696	0.158	0.026	6.2E-10	38.3
HbA1c	Men	rs12219514	G	A	0.434	-0.207	0.023	1.1E-18	78.0
HbA1c	Men	rs1502276	A	G	0.184	-0.176	0.030	5.4E-09	34.0
HbA1c	Men	rs7127313	T	C	0.346	0.198	0.024	6.2E-16	65.4
HbA1c	Men	rs6484302	T	C	0.176	0.223	0.031	4.3E-13	52.5
HbA1c	Men	rs7124355	G	A	0.675	-0.138	0.025	3.1E-08	30.7
HbA1c	Men	rs12419995	T	A	0.656	-0.235	0.026	5.3E-20	83.9
HbA1c	Men	rs2237895	C	A	0.415	0.211	0.024	3.0E-19	80.5
HbA1c	Men	rs11039165	G	A	0.271	-0.167	0.026	1.6E-10	40.9
HbA1c	Men	rs174533	A	G	0.347	-0.192	0.024	3.5E-15	62.0
HbA1c	Men	rs9667947	C	T	0.161	-0.252	0.032	1.6E-15	63.5
HbA1c	Men	rs10830963	G	C	0.275	0.436	0.026	1.1E-63	284.1
HbA1c	Men	rs360140	A	C	0.643	-0.283	0.024	2.1E-31	135.9
HbA1c	Men	rs7137828	T	C	0.517	0.200	0.023	9.5E-18	73.6
HbA1c	Men	rs112066678	C	A	0.054	0.294	0.051	9.3E-09	33.0
HbA1c	Men	rs9668691	C	T	0.232	-0.202	0.028	2.5E-13	53.5
HbA1c	Men	rs4238013	T	C	0.796	-0.165	0.029	1.5E-08	32.1
HbA1c	Men	rs76895963	G	T	0.020	-1.488	0.089	2.3E-62	278.0
HbA1c	Men	rs12819124	A	C	0.472	-0.451	0.023	2.6E-83	374.4
HbA1c	Men	rs6580661	T	A	0.224	0.215	0.028	1.4E-14	59.3
HbA1c	Men	rs143343646	G	A	0.063	-0.266	0.049	4.5E-08	29.9
HbA1c	Men	rs79755767	A	G	0.100	-0.243	0.039	5.6E-10	38.5
HbA1c	Men	rs343087	G	A	0.861	-0.191	0.034	1.3E-08	32.3
HbA1c	Men	rs79621919	A	G	0.059	-0.299	0.049	1.5E-09	36.6
HbA1c	Men	rs420431	G	C	0.122	0.482	0.035	4.0E-42	185.1
HbA1c	Men	rs7329468	T	C	0.217	-0.270	0.028	2.6E-21	89.8

HbA1c	Men	rs11149501	C	T	0.223	0.203	0.028	5.2E-13	52.1
HbA1c	Men	rs2858980	A	G	0.833	-0.264	0.031	2.2E-17	72.0
HbA1c	Men	rs1359790	A	G	0.289	-0.199	0.026	6.9E-15	60.6
HbA1c	Men	rs561278583	C	G	0.206	0.168	0.029	5.3E-09	34.1
HbA1c	Men	rs12886807	T	C	0.242	-0.153	0.028	3.5E-08	30.4
HbA1c	Men	rs12434326	C	G	0.249	-0.177	0.027	4.8E-11	43.3
HbA1c	Men	rs2285005	A	G	0.340	0.203	0.025	1.6E-16	68.1
HbA1c	Men	rs10873398	A	G	0.646	-0.162	0.024	2.6E-11	44.5
HbA1c	Men	rs113204516	G	A	0.049	-0.350	0.054	1.1E-10	41.6
HbA1c	Men	rs7167567	A	T	0.423	0.155	0.024	4.6E-11	43.3
HbA1c	Men	rs11633054	G	A	0.713	0.210	0.026	3.3E-16	66.6
HbA1c	Men	rs62022948	T	C	0.329	-0.138	0.025	2.5E-08	31.0
HbA1c	Men	rs1866476	C	T	0.732	-0.170	0.026	1.0E-10	41.8
HbA1c	Men	rs396263	G	A	0.211	0.161	0.029	1.5E-08	32.1
HbA1c	Men	rs165975	C	T	0.234	0.173	0.028	3.2E-10	39.6
HbA1c	Men	rs184499898	G	A	0.011	0.715	0.116	6.4E-10	38.2
HbA1c	Men	rs181205	C	T	0.343	0.208	0.025	3.9E-17	70.8
HbA1c	Men	rs2858010	C	T	0.340	-0.185	0.025	5.8E-14	56.4
HbA1c	Men	rs8050500	C	T	0.446	-0.199	0.023	2.0E-17	72.1
HbA1c	Men	rs7188250	C	T	0.414	0.175	0.024	1.3E-13	54.9
HbA1c	Men	rs35158985	G	A	0.308	0.162	0.025	1.3E-10	41.3
HbA1c	Men	rs247826	T	C	0.223	0.229	0.028	3.0E-16	66.8
HbA1c	Men	rs72811487	A	G	0.035	-0.357	0.063	1.8E-08	31.7
HbA1c	Men	rs551118	G	C	0.578	0.394	0.024	2.9E-61	272.9
HbA1c	Men	rs1108646	G	A	0.682	-0.243	0.025	4.8E-22	93.2
HbA1c	Men	rs71373617	T	C	0.320	-0.162	0.025	9.4E-11	42.0
HbA1c	Men	rs216195	G	T	0.303	0.154	0.025	9.9E-10	37.3
HbA1c	Men	rs9303620	C	T	0.278	-0.201	0.026	9.3E-15	60.1
HbA1c	Men	rs17698176	G	T	0.229	0.173	0.028	6.0E-10	38.3
HbA1c	Men	rs12600858	A	G	0.224	-0.237	0.028	2.2E-17	72.0
HbA1c	Men	rs8064261	A	G	0.275	-0.157	0.026	1.8E-09	36.2
HbA1c	Men	rs1030097	G	C	0.495	0.128	0.023	4.1E-08	30.1
HbA1c	Men	rs1641523	T	C	0.567	-0.245	0.024	3.8E-25	107.4
HbA1c	Men	rs2748427	G	A	0.218	0.436	0.028	3.6E-54	240.4
HbA1c	Men	rs113373052	T	C	0.313	0.463	0.025	7.7E-76	340.0
HbA1c	Men	rs8078338	G	A	0.506	-0.169	0.023	4.0E-13	52.6
HbA1c	Men	rs6507691	C	T	0.749	0.184	0.027	7.7E-12	46.8
HbA1c	Men	rs17533945	C	T	0.392	0.337	0.024	6.9E-45	197.7
HbA1c	Men	rs10401969	C	T	0.077	0.263	0.044	2.0E-09	36.0
HbA1c	Men	rs75372982	G	A	0.289	-0.175	0.026	1.6E-11	45.4
HbA1c	Men	rs584007	G	A	0.641	-0.184	0.024	3.6E-14	57.4
HbA1c	Men	rs35255921	A	G	0.013	-0.581	0.101	9.2E-09	33.0
HbA1c	Men	rs62111724	C	T	0.366	-0.207	0.024	2.4E-17	71.8
HbA1c	Men	rs12985029	A	G	0.340	-0.157	0.025	1.6E-10	40.9
HbA1c	Men	rs1975283	C	A	0.685	-0.164	0.025	6.6E-11	42.6
HbA1c	Men	rs587739738	T	C	0.083	-0.284	0.042	1.8E-11	45.2
HbA1c	Men	rs857720	C	T	0.267	0.591	0.026	4.1E-112	507.1
HbA1c	Men	rs2228445	C	T	0.902	0.283	0.039	4.5E-13	52.4

HbA1c	Men	rs79687284	C	G	0.035	0.603	0.063	1.5E-21	90.9
HbA1c	Men	rs238763	A	T	0.592	-0.169	0.024	1.1E-12	50.6
HbA1c	Men	rs3811444	T	C	0.334	0.192	0.025	5.8E-15	61.0
HbA1c	Men	rs4518840	T	A	0.878	-0.279	0.036	9.2E-15	60.1
HbA1c	Men	rs114247628	A	G	0.006	-1.373	0.159	5.1E-18	74.9
HbA1c	Men	rs1175550	G	A	0.230	-0.304	0.028	2.4E-27	117.4
HbA1c	Men	rs3768321	T	G	0.197	0.212	0.029	4.6E-13	52.4
HbA1c	Men	rs3789586	T	C	0.153	-0.248	0.032	1.7E-14	58.9
HbA1c	Men	rs7513688	A	G	0.361	0.149	0.024	9.3E-10	37.5
HbA1c	Men	rs2065703	T	C	0.159	0.179	0.032	1.9E-08	31.6
HbA1c	Men	rs6063050	C	T	0.283	-0.162	0.026	4.8E-10	38.8
HbA1c	Men	rs6014993	G	A	0.487	0.146	0.023	4.0E-10	39.1
HbA1c	Men	rs2427361	A	C	0.620	-0.151	0.024	4.9E-10	38.7
HbA1c	Men	rs6086540	C	G	0.507	-0.173	0.023	1.2E-13	55.0
HbA1c	Men	rs17850433	C	T	0.012	0.647	0.108	1.8E-09	36.2
HbA1c	Men	rs59923178	G	T	0.096	-0.251	0.039	2.0E-10	40.5
HbA1c	Men	rs77696994	T	C	0.076	-0.301	0.044	1.0E-11	46.3
HbA1c	Men	rs855791	G	A	0.561	-0.428	0.023	3.3E-74	332.5
HbA1c	Men	rs8138197	A	G	0.471	-0.210	0.023	1.7E-19	81.6
HbA1c	Men	rs830021	C	A	0.697	-0.153	0.025	1.7E-09	36.3
HbA1c	Men	rs560887	C	T	0.701	0.685	0.025	1.1E-161	735.8
HbA1c	Men	rs13031216	T	C	0.220	-0.195	0.028	3.4E-12	48.4
HbA1c	Men	rs1399623	C	A	0.623	0.186	0.024	1.7E-14	58.9
HbA1c	Men	rs838717	A	G	0.566	0.133	0.023	1.3E-08	32.3
HbA1c	Men	rs113362373	G	A	0.135	0.269	0.034	2.9E-15	62.3
HbA1c	Men	rs780093	C	T	0.618	0.237	0.024	3.2E-23	98.6
HbA1c	Men	rs10173636	G	C	0.130	-0.456	0.035	8.9E-40	174.3
HbA1c	Men	rs2164701	C	G	0.140	0.235	0.034	2.6E-12	49.0
HbA1c	Men	rs77000664	G	A	0.177	0.169	0.031	3.5E-08	30.4
HbA1c	Men	rs7606173	C	G	0.431	-0.182	0.023	8.1E-15	60.3
HbA1c	Men	rs7572278	A	T	0.204	0.214	0.029	1.1E-13	55.2
HbA1c	Men	rs7638232	A	G	0.418	-0.263	0.024	6.3E-29	124.6
HbA1c	Men	rs11708067	G	A	0.245	-0.313	0.027	6.0E-31	133.9
HbA1c	Men	rs12497133	A	G	0.533	0.148	0.023	2.6E-10	39.9
HbA1c	Men	rs6785881	T	C	0.481	-0.153	0.023	5.8E-11	42.9
HbA1c	Men	rs1905505	A	G	0.284	-0.283	0.026	5.2E-28	120.4
HbA1c	Men	rs12633493	T	C	0.586	0.184	0.024	7.6E-15	60.5
HbA1c	Men	rs6780171	A	T	0.313	0.210	0.025	6.9E-17	69.7
HbA1c	Men	rs2041965	T	C	0.344	-0.144	0.024	3.9E-09	34.7
HbA1c	Men	rs6777684	G	A	0.610	0.178	0.024	1.2E-13	55.0
HbA1c	Men	rs1496653	G	A	0.204	-0.159	0.029	3.5E-08	30.4
HbA1c	Men	rs12715435	A	G	0.171	0.267	0.031	7.8E-18	74.0
HbA1c	Men	rs11728350	G	A	0.125	0.198	0.035	1.8E-08	31.7
HbA1c	Men	rs9994509	G	A	0.393	-0.134	0.024	1.9E-08	31.6
HbA1c	Men	rs2044341	C	T	0.684	-0.358	0.025	2.5E-46	204.4
HbA1c	Men	rs17462188	T	C	0.690	0.152	0.025	2.2E-09	35.8
HbA1c	Men	rs111537480	G	A	0.138	-0.216	0.034	2.4E-10	40.1
HbA1c	Men	rs225176	G	C	0.671	-0.146	0.026	1.3E-08	32.3

HbA1c	Men	rs59816691	G	C	0.669	0.137	0.025	3.5E-08	30.4
HbA1c	Men	rs258217	G	A	0.464	-0.157	0.024	5.1E-11	43.2
HbA1c	Men	rs7720275	C	T	0.170	0.180	0.031	6.9E-09	33.6
HbA1c	Men	rs7732130	A	G	0.681	-0.249	0.025	1.4E-23	100.1
HbA1c	Men	rs34533653	A	T	0.266	-0.149	0.026	1.5E-08	32.0
HbA1c	Men	rs12210538	G	A	0.236	-0.183	0.027	2.1E-11	44.8
HbA1c	Men	rs56293029	A	C	0.259	-0.308	0.027	2.0E-30	131.4
HbA1c	Men	rs62440928	A	G	0.607	-0.158	0.024	5.1E-11	43.2
HbA1c	Men	rs12201779	T	C	0.395	-0.143	0.024	2.2E-09	35.8
HbA1c	Men	rs2665357	C	A	0.517	0.142	0.023	1.1E-09	37.1
HbA1c	Men	rs67131976	T	C	0.172	0.355	0.031	9.1E-31	133.0
HbA1c	Men	rs1800562	A	G	0.079	-0.815	0.043	2.7E-79	355.9
HbA1c	Men	rs204991	C	T	0.221	0.161	0.028	9.5E-09	32.9
HbA1c	Men	rs3135365	A	C	0.769	0.257	0.028	1.2E-20	86.9
HbA1c	Men	rs2308891	A	C	0.459	0.164	0.024	1.2E-11	46.1
HbA1c	Men	rs3778321	A	G	0.180	-0.180	0.030	2.7E-09	35.4
HbA1c	Men	rs12667932	A	G	0.080	-0.347	0.043	7.0E-16	65.2
HbA1c	Men	rs11763864	T	A	0.157	0.218	0.032	8.8E-12	46.6
HbA1c	Men	rs11514706	C	A	0.524	0.223	0.023	1.3E-21	91.2
HbA1c	Men	rs11982272	C	T	0.202	-0.160	0.029	4.2E-08	30.0
HbA1c	Men	rs1708302	T	C	0.501	-0.192	0.023	1.6E-16	68.0
HbA1c	Men	rs76323047	G	A	0.118	0.336	0.036	1.1E-20	86.9
HbA1c	Men	rs1004558	T	C	0.179	0.685	0.030	1.0E-112	509.9
HbA1c	Men	rs55803206	A	C	0.464	0.133	0.024	2.0E-08	31.5
HbA1c	Men	rs117370443	C	T	0.039	0.419	0.060	3.1E-12	48.6
HbA1c	Men	rs4300038	A	G	0.311	-0.394	0.025	3.9E-55	244.8
HbA1c	Men	rs2954021	G	A	0.506	0.211	0.023	1.0E-19	82.5
HbA1c	Men	rs2060985	G	A	0.537	-0.130	0.023	2.2E-08	31.3
HbA1c	Men	rs3757974	G	A	0.380	0.148	0.024	5.4E-10	38.5
HbA1c	Men	rs66593272	T	A	0.038	-0.982	0.061	2.9E-58	259.2
HbA1c	Men	rs4737010	A	G	0.228	0.603	0.028	1.5E-104	472.3
HbA1c	Men	rs55840085	A	G	0.355	0.184	0.024	3.7E-14	57.3
HbA1c	Men	rs7829826	A	T	0.534	-0.145	0.023	4.9E-10	38.7
HbA1c	Men	rs1967604	G	A	0.712	-0.210	0.026	3.4E-16	66.6
HbA1c	Men	rs550057	T	C	0.255	0.368	0.027	3.3E-43	190.0
HbA1c	Men	rs28641468	C	T	0.756	0.211	0.027	5.8E-15	61.0
HbA1c	Men	rs10811660	A	G	0.173	-0.387	0.031	2.7E-36	158.4
HbA1c	Men	rs10758593	A	G	0.397	0.141	0.024	2.8E-09	35.3
HbA1c	Men	rs17803780	C	T	0.211	-0.169	0.028	2.7E-09	35.4
HbA1c	Men	rs7025532	C	T	0.199	0.261	0.029	2.4E-19	80.9
HbA1c	Men	rs61750929	T	C	0.055	-0.668	0.051	2.8E-39	172.0
HbA1c	Men	rs7047279	C	T	0.568	-0.161	0.024	9.1E-12	46.5

BMI, body mass index; HbA1c, glycated hemoglobin.

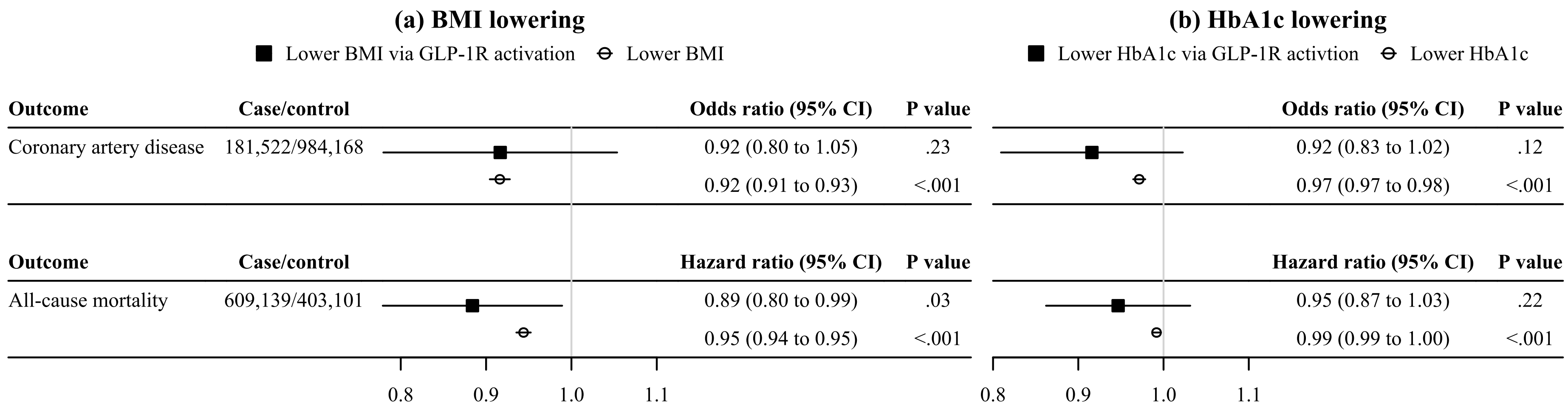

eFigure 3. IVW MR estimates for the associations of GLP-1R activation with the risk of coronary artery disease and all-cause mortality in comparison with those for lower BMI and lower HbA1c.

BMI, body mass index; GLP-1R, glucagon-like peptide-1 receptor; HbA1c, glycated hemoglobin; IVW, inverse variance weighted; MR, Mendelian randomization. Black squares denote genetically predicted (a) lower BMI or (b) lower HbA1c via GLP-1R activation based on variants within 1 Mb of *GLP1R*. White circles denote genetically predicted (a) lower BMI or (b) lower HbA1c based on genome-wide variants. Estimates are presented as odds ratio for coronary artery disease or hazard ratio for all-cause mortality per 1-kg/m^2 decrease in BMI or per 1-mmol/mol decrease in HbA1c.

eTable 6. MR sensitivity analyses for the associations of lower BMI and lower HbA1c with the risk of coronary artery disease and all-cause mortality.

Exposure	Outcome	SNPs	Method	Estimate (95% CI)	Measure	*P* value	*P* value (intercept)	*P* value (Q)
Lower BMI	Coronary artery disease	505	IVW	0.92 (0.91 to 0.93)	Odds ratio	<0.001		<0.001
Lower BMI	Coronary artery disease	505	Weighted median	0.92 (0.91 to 0.93)	Odds ratio	<0.001		
Lower BMI	Coronary artery disease	505	MR Egger	0.92 (0.90 to 0.95)	Odds ratio	<0.001	0.74	
Lower BMI	Coronary artery disease	505	MR-RAPS	0.91 (0.91 to 0.92)	Odds ratio	<0.001		
Lower BMI	All-cause mortality	490	IVW	0.95 (0.94 to 0.95)	Hazard ratio	<0.001		<0.001
Lower BMI	All-cause mortality	490	Weighted median	0.94 (0.93 to 0.95)	Hazard ratio	<0.001		
Lower BMI	All-cause mortality	490	MR Egger	0.95 (0.93 to 0.97)	Hazard ratio	<0.001	0.51	
Lower BMI	All-cause mortality	490	MR-RAPS	0.94 (0.93 to 0.94)	Hazard ratio	<0.001		
Lower BMI	All-cause mortality	489	IVW (Steiger filtering)	0.94 (0.94 to 0.95)	Hazard ratio	<0.001		<0.001
Lower BMI	All-cause mortality	489	Weighted median (Steiger filtering)	0.94 (0.93 to 0.95)	Hazard ratio	<0.001		
Lower BMI	All-cause mortality	489	MR Egger (Steiger filtering)	0.94 (0.93 to 0.96)	Hazard ratio	<0.001	0.86	
Lower BMI	All-cause mortality	489	MR-RAPS (Steiger filtering)	0.94 (0.93 to 0.94)	Hazard ratio	<0.001		
Lower HbA1c	Coronary artery disease	303	IVW	0.97 (0.97 to 0.98)	Odds ratio	<0.001		<0.001
Lower HbA1c	Coronary artery disease	303	Weighted median	0.98 (0.97 to 0.98)	Odds ratio	<0.001		
Lower HbA1c	Coronary artery disease	303	MR Egger	0.99 (0.97 to 1.00)	Odds ratio	0.04	0.006	
Lower HbA1c	Coronary artery disease	303	MR-RAPS	0.97 (0.97 to 0.98)	Odds ratio	<0.001		
Lower HbA1c	All-cause mortality	286	IVW	0.99 (0.99 to 1.00)	Hazard ratio	<0.001		<0.001
Lower HbA1c	All-cause mortality	286	Weighted median	1.00 (0.99 to 1.00)	Hazard ratio	0.05		
Lower HbA1c	All-cause mortality	286	MR Egger	1.00 (1.00 to 1.01)	Hazard ratio	0.14	<0.001	
Lower HbA1c	All-cause mortality	286	MR-RAPS	0.99 (0.99 to 0.99)	Hazard ratio	<0.001		

BMI, body mass index; GLP-1R, glucagon-like peptide-1 receptor; HbA1c, glycated hemoglobin; IVW, inverse variance weighted; MR, Mendelian randomization; RAPS, robust adjusted profile score. Estimates are presented per 1-kg/m^2 decrease in BMI or per 1-mmol/mol decrease in HbA1c.

(a) BMI lowering

■ Lower BMI via GLP-1R activation ⊖ Lower BMI

(b) HbA1c lowering

■ Lower HbA1c via GLP-1R activation ⊖ Lower HbA1c

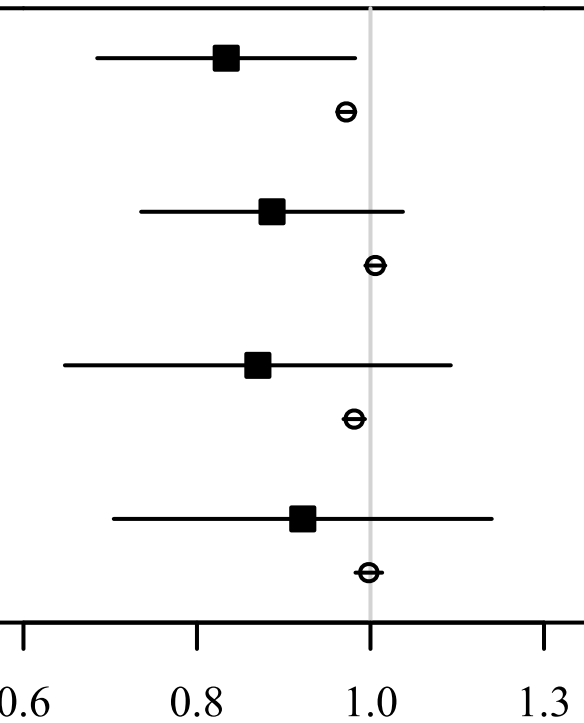

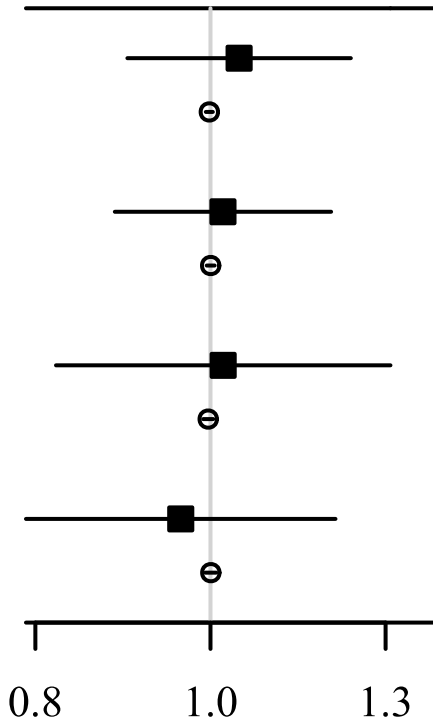

Outcome	Sex	Case/control	(a) Odds ratio (95% CI)	P	P (sex diff)	(b) Odds ratio (95% CI)	P	P (sex diff)
Depressive symptoms	Women	79,976/112,704	0.81 (0.67 to 0.98)	.03	.62	1.04 (0.89 to 1.22)	.62	.84
(seen a general practitioner)			0.97 (0.95 to 0.98)	<.001	<.001	1.00 (0.99 to 1.00)	.53	.62
	Men	43,552/122,461	0.87 (0.72 to 1.05)	.14	.62	1.02 (0.87 to 1.19)	.82	.84
			1.01 (0.99 to 1.02)	.34	<.001	1.00 (0.99 to 1.01)	.92	.62
Depressive symptoms	Women	23,975/169,269	0.85 (0.64 to 1.12)	.25	.74	1.02 (0.80 to 1.29)	.88	.72
(seen a psychiatrist)			0.98 (0.96 to 0.99)	.003	.09	1.00 (0.99 to 1.00)	.38	.49
	Men	17,258/149,033	0.91 (0.69 to 1.19)	.48	.74	0.96 (0.77 to 1.20)	.71	.72
			1.00 (0.98 to 1.02)	.81	.09	1.00 (0.99 to 1.01)	.90	.49

eFigure 4. IVW MR estimates for sex-specific associations of GLP-1R activation with the risk of depressive symptoms in comparison with those for lower BMI and lower HbA1c.

BMI, body mass index; GLP-1R, glucagon-like peptide-1 receptor; HbA1c, glycated hemoglobin; IVW, inverse variance weighted; MR, Mendelian randomization. Black squares denote genetically predicted (a) lower BMI or (b) lower HbA1c via GLP-1R activation based on variants within 1 Mb of *GLP1R*. White circles denote genetically predicted (a) lower BMI or (b) lower HbA1c based on genome-wide variants. Estimates are presented as odds ratio per 1-kg/m^2 decrease in BMI or per 1-mmol/mol decrease in HbA1c.

eTable 7. MR sensitivity analyses for the associations of lower BMI and lower HbA1c with mental health well-being.

Exposure	Outcome	Sex	SNPs	Method	Estimate (95% CI)	Measure	*P* value	*P* value (intercept)	*P* value (Q)
Lower BMI	Well-being spectrum	Overall	464	IVW	0.003 (0.000 to 0.01)	Beta	0.04		<0.001
Lower BMI	Well-being spectrum	Overall	464	Weighted median	0.002 (-0.000 to 0.005)	Beta	0.08		
Lower BMI	Well-being spectrum	Overall	464	MR Egger	-0.001 (-0.01 to 0.01)	Beta	0.81	0.3	
Lower BMI	Well-being spectrum	Overall	464	MR-RAPS	0.003 (0.002 to 0.005)	Beta	<0.001		
Lower BMI	Life satisfaction	Overall	464	IVW	0.003 (-0.000 to 0.01)	Beta	0.05		<0.001
Lower BMI	Life satisfaction	Overall	464	Weighted median	0.002 (-0.001 to 0.01)	Beta	0.24		
Lower BMI	Life satisfaction	Overall	464	MR Egger	-0.001 (-0.01 to 0.01)	Beta	0.8	0.33	
Lower BMI	Life satisfaction	Overall	464	MR-RAPS	0.004 (0.002 to 0.01)	Beta	<0.001		
Lower BMI	Positive affect	Overall	464	IVW	0.002 (-0.001 to 0.01)	Beta	0.16		<0.001
Lower BMI	Positive affect	Overall	464	Weighted median	0.002 (-0.002 to 0.01)	Beta	0.29		
Lower BMI	Positive affect	Overall	464	MR Egger	-0.001 (-0.01 to 0.01)	Beta	0.76	0.41	
Lower BMI	Positive affect	Overall	464	MR-RAPS	0.004 (0.002 to 0.01)	Beta	<0.001		
Lower BMI	Neuroticism	Overall	464	IVW	-0.004 (-0.01 to 0.000)	Beta	0.05		<0.001
Lower BMI	Neuroticism	Overall	464	Weighted median	-0.005 (-0.01 to -0.001)	Beta	0.02		
Lower BMI	Neuroticism	Overall	464	MR Egger	0.002 (-0.01 to 0.01)	Beta	0.71	0.27	
Lower BMI	Neuroticism	Overall	464	MR-RAPS	-0.01 (-0.01 to -0.003)	Beta	<0.001		
Lower BMI	Depressive symptoms	Overall	464	IVW	-0.004 (-0.01 to -0.001)	Beta	0.002		<0.001
Lower BMI	Depressive symptoms	Overall	464	Weighted median	-0.003 (-0.01 to -0.001)	Beta	0.02		
Lower BMI	Depressive symptoms	Overall	464	MR Egger	-0.001 (-0.01 to 0.01)	Beta	0.85	0.35	
Lower BMI	Depressive symptoms	Overall	464	MR-RAPS	-0.005 (-0.01 to -0.003)	Beta	<0.001		
Lower BMI	Depressive symptoms (seen a general practitioner)	Women	300	IVW	0.97 (0.95 to 0.98)	Odds ratio	<0.001		<0.001
Lower BMI	Depressive symptoms (seen a general practitioner)	Women	300	Weighted median	0.98 (0.96 to 0.99)	Odds ratio	0.01		

Lower BMI	Depressive symptoms (seen a general practitioner)	Women	300	MR Egger	0.99 (0.96 to 1.03)	Odds ratio	0.75	0.1	
Lower BMI	Depressive symptoms (seen a general practitioner)	Women	300	MR-RAPS	0.97 (0.96 to 0.98)	Odds ratio	<0.001		
Lower BMI	Depressive symptoms (seen a general practitioner)	Men	251	IVW	1.01 (0.99 to 1.02)	Odds ratio	0.34		<0.001
Lower BMI	Depressive symptoms (seen a general practitioner)	Men	251	Weighted median	1.01 (0.99 to 1.03)	Odds ratio	0.37		
Lower BMI	Depressive symptoms (seen a general practitioner)	Men	251	MR Egger	1.00 (0.96 to 1.04)	Odds ratio	1	0.7	
Lower BMI	Depressive symptoms (seen a general practitioner)	Men	251	MR-RAPS	1.01 (1.00 to 1.02)	Odds ratio	0.11		
Lower BMI	Depressive symptoms (seen a psychiatrist)	Women	300	IVW	0.98 (0.96 to 0.99)	Odds ratio	0.003		<0.001
Lower BMI	Depressive symptoms (seen a psychiatrist)	Women	300	Weighted median	0.99 (0.97 to 1.01)	Odds ratio	0.25		
Lower BMI	Depressive symptoms (seen a psychiatrist)	Women	300	MR Egger	0.99 (0.95 to 1.04)	Odds ratio	0.75	0.43	
Lower BMI	Depressive symptoms (seen a psychiatrist)	Women	300	MR-RAPS	0.98 (0.96 to 0.99)	Odds ratio	<0.001		
Lower BMI	Depressive symptoms (seen a psychiatrist)	Men	251	IVW	1.00 (0.98 to 1.02)	Odds ratio	0.81		<0.001
Lower BMI	Depressive symptoms (seen a psychiatrist)	Men	251	Weighted median	1.01 (0.98 to 1.03)	Odds ratio	0.61		
Lower BMI	Depressive symptoms (seen a psychiatrist)	Men	251	MR Egger	1.01 (0.95 to 1.06)	Odds ratio	0.85	0.76	
Lower BMI	Depressive symptoms (seen a psychiatrist)	Men	251	MR-RAPS	1.00 (0.98 to 1.02)	Odds ratio	0.94		

Lower HbA1c	Well-being spectrum	Overall	242	IVW	-0.001 (-0.002 to 0.001)	Beta	0.35		<0.001
Lower HbA1c	Well-being spectrum	Overall	242	Weighted median	0.000 (-0.001 to 0.001)	Beta	0.83		
Lower HbA1c	Well-being spectrum	Overall	242	MR Egger	-0.000 (-0.002 to 0.002)	Beta	0.78	0.81	
Lower HbA1c	Well-being spectrum	Overall	242	MR-RAPS	-0.000 (-0.001 to 0.000)	Beta	0.42		
Lower HbA1c	Life satisfaction	Overall	242	IVW	-0.001 (-0.002 to 0.001)	Beta	0.42		<0.001
Lower HbA1c	Life satisfaction	Overall	242	Weighted median	-0.000 (-0.002 to 0.002)	Beta	0.96		
Lower HbA1c	Life satisfaction	Overall	242	MR Egger	-0.000 (-0.003 to 0.002)	Beta	0.73	0.93	
Lower HbA1c	Life satisfaction	Overall	242	MR-RAPS	-0.000 (-0.001 to 0.001)	Beta	0.49		
Lower HbA1c	Positive affect	Overall	242	IVW	-0.001 (-0.002 to 0.001)	Beta	0.38		<0.001
Lower HbA1c	Positive affect	Overall	242	Weighted median	-0.000 (-0.002 to 0.002)	Beta	0.88		
Lower HbA1c	Positive affect	Overall	242	MR Egger	-0.000 (-0.003 to 0.002)	Beta	0.74	0.88	
Lower HbA1c	Positive affect	Overall	242	MR-RAPS	-0.000 (-0.001 to 0.001)	Beta	0.35		
Lower HbA1c	Neuroticism	Overall	242	IVW	0.001 (-0.001 to 0.002)	Beta	0.54		<0.001
Lower HbA1c	Neuroticism	Overall	242	Weighted median	-0.000 (-0.002 to 0.002)	Beta	0.74		
Lower HbA1c	Neuroticism	Overall	242	MR Egger	0.000 (-0.003 to 0.004)	Beta	0.81	0.93	
Lower HbA1c	Neuroticism	Overall	242	MR-RAPS	0.000 (-0.001 to 0.001)	Beta	0.63		
Lower HbA1c	Depressive symptoms	Overall	242	IVW	0.000 (-0.001 to 0.001)	Beta	0.63		<0.001
Lower HbA1c	Depressive symptoms	Overall	242	Weighted median	-0.000 (-0.001 to 0.001)	Beta	0.77		
Lower HbA1c	Depressive symptoms	Overall	242	MR Egger	0.000 (-0.002 to 0.003)	Beta	0.68	0.85	
Lower HbA1c	Depressive symptoms	Overall	242	MR-RAPS	0.000 (-0.001 to 0.001)	Beta	0.71		
Lower HbA1c	Depressive symptoms (seen a general practitioner)	Women	193	IVW	1.00 (0.99 to 1.00)	Odds ratio	0.53		<0.001
Lower HbA1c	Depressive symptoms (seen a general practitioner)	Women	193	Weighted median	1.00 (0.99 to 1.01)	Odds ratio	0.68		
Lower HbA1c	Depressive symptoms (seen a general practitioner)	Women	193	MR Egger	1.00 (0.99 to 1.01)	Odds ratio	0.85	0.49	
Lower HbA1c	Depressive symptoms (seen a general practitioner)	Women	193	MR-RAPS	1.00 (0.99 to 1.00)	Odds ratio	0.43		

Lower HbA1c	Depressive symptoms (seen a general practitioner)	Men	168	IVW	1.00 (0.99 to 1.01)	Odds ratio	0.92		0.005
Lower HbA1c	Depressive symptoms (seen a general practitioner)	Men	168	Weighted median	1.00 (0.99 to 1.01)	Odds ratio	0.5		
Lower HbA1c	Depressive symptoms (seen a general practitioner)	Men	168	MR Egger	1.00 (0.99 to 1.01)	Odds ratio	0.9	0.93	
Lower HbA1c	Depressive symptoms (seen a general practitioner)	Men	168	MR-RAPS	1.00 (1.00 to 1.01)	Odds ratio	0.6		
Lower HbA1c	Depressive symptoms (seen a psychiatrist)	Women	193	IVW	1.00 (0.99 to 1.00)	Odds ratio	0.38		0.003
Lower HbA1c	Depressive symptoms (seen a psychiatrist)	Women	193	Weighted median	0.99 (0.98 to 1.00)	Odds ratio	0.21		
Lower HbA1c	Depressive symptoms (seen a psychiatrist)	Women	193	MR Egger	1.00 (0.99 to 1.01)	Odds ratio	0.9	0.43	
Lower HbA1c	Depressive symptoms (seen a psychiatrist)	Women	193	MR-RAPS	1.00 (0.99 to 1.00)	Odds ratio	0.15		
Lower HbA1c	Depressive symptoms (seen a psychiatrist)	Men	168	IVW	1.00 (0.99 to 1.01)	Odds ratio	0.9		0.11
Lower HbA1c	Depressive symptoms (seen a psychiatrist)	Men	168	Weighted median	1.00 (0.98 to 1.01)	Odds ratio	0.45		
Lower HbA1c	Depressive symptoms (seen a psychiatrist)	Men	168	MR Egger	1.00 (0.98 to 1.01)	Odds ratio	0.61	0.49	
Lower HbA1c	Depressive symptoms (seen a psychiatrist)	Men	168	MR-RAPS	1.00 (0.99 to 1.01)	Odds ratio	0.85		

BMI, body mass index; HbA1c, glycated hemoglobin; IVW, inverse variance weighting; MR, Mendelian randomization; RAPS, robust adjusted profile score. Positive associations with the well-being spectrum, life satisfaction, and positive affect and negative associations with neuroticism and depressive symptoms indicate better mental health well-being. Estimates are presented per 1-kg/m^2 decrease in BMI or per 1-mmol/mol decrease in HbA1c.

eFigure 5. IVW MR estimates for sex-specific associations of GLP-1R activation with the risk of major depressive disorder, schizophrenia, and ADHD in comparison with those for lower BMI and lower HbA1c.

(a) BMI lowering — ■ Lower BMI via GLP-1R activation ⊖ Lower BMI

(b) HbA1c lowering — ■ Lower HbA1c via GLP-1R activation ⊖ Lower HbA1c

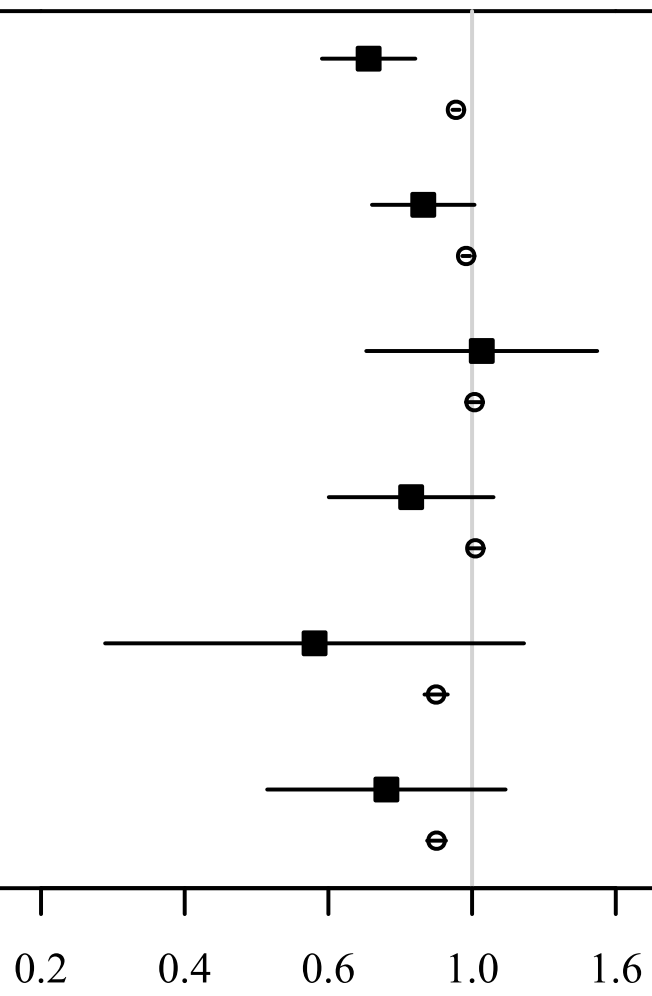

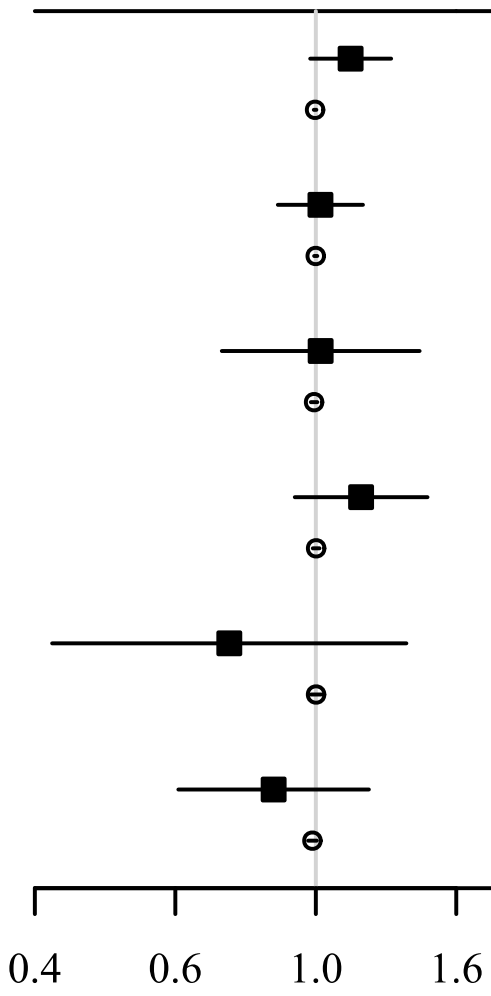

Outcome	Sex	Case/control	(a) Odds ratio (95% CI)	(a) P	(a) P (sex diff)	(b) Odds ratio (95% CI)	(b) P	(b) P (sex diff)
Major depressive disorder	Women	130,471/159,521	0.70 (0.59 to 0.82)	<.001	.12	1.13 (0.98 to 1.31)	.09	.31
			0.95 (0.93 to 0.96)	<.001	<.001	1.00 (0.99 to 1.00)	.27	.55
	Men	64,805/132,185	0.84 (0.71 to 1.01)	.06	.12	1.02 (0.87 to 1.18)	.83	.31
			0.98 (0.97 to 0.99)	.003	<.001	1.00 (0.99 to 1.01)	.90	.55
Schizophrenia	Women	17,710/36,803	1.03 (0.69 to 1.55)	.87	.33	1.02 (0.72 to 1.45)	.92	.51
			1.01 (0.98 to 1.04)	.57	.89	0.99 (0.98 to 1.01)	.30	.40
	Men	33,097/35,190	0.81 (0.61 to 1.08)	.15	.33	1.17 (0.93 to 1.49)	.18	.51
			1.01 (0.99 to 1.04)	.39	.89	1.00 (0.99 to 1.01)	.86	.40
ADHD	Women	4,945/16,246	0.58 (0.28 to 1.20)	.14	.56	0.73 (0.39 to 1.38)	.34	.67
			0.88 (0.85 to 0.92)	<.001	.95	1.00 (0.98 to 1.02)	.92	.29
	Men	14,154/17,948	0.74 (0.49 to 1.12)	.16	.56	0.86 (0.61 to 1.21)	.39	.67
			0.88 (0.86 to 0.91)	<.001	.95	0.99 (0.97 to 1.00)	.12	.29

ADHD, attention deficit hyperactivity disorder; BMI, body mass index; GLP-1R, glucagon-like peptide-1 receptor; HbA1c, glycated hemoglobin; IVW, inverse variance weighted; MR, Mendelian randomization. Black squares denote genetically predicted (a) lower BMI or (b) lower HbA1c via GLP-1R activation based on variants within 1 Mb of *GLP1R*. White circles denote genetically predicted (a) lower BMI or (b) lower HbA1c based on genome-wide variants. Estimates are presented as odds ratio per 1-kg/m^2 decrease in BMI or per 1-mmol/mol decrease in HbA1c.

eTable 8. MR sensitivity analyses for the associations of lower BMI and lower HbA1c with the risk of mental health disorders.

Exposure	Outcome	Sex	SNPs	Method	Odds ratio (95% CI)	*P* value	*P* value (intercept)	*P* value (Q)
Lower BMI	Major depressive disorder	Overall	492	IVW	0.97 (0.96 to 0.98)	<0.001		<0.001
Lower BMI	Major depressive disorder	Overall	492	Weighted median	0.97 (0.96 to 0.98)	<0.001		
Lower BMI	Major depressive disorder	Overall	492	MR Egger	0.99 (0.97 to 1.01)	0.26	0.04	
Lower BMI	Major depressive disorder	Overall	492	MR-RAPS	0.97 (0.97 to 0.97)	<0.001		
Lower BMI	Major depressive disorder	Women	297	IVW	0.95 (0.93 to 0.96)	<0.001		<0.001
Lower BMI	Major depressive disorder	Women	297	Weighted median	0.95 (0.94 to 0.97)	<0.001		
Lower BMI	Major depressive disorder	Women	297	MR Egger	0.96 (0.92 to 0.99)	0.02	0.54	
Lower BMI	Major depressive disorder	Women	297	MR-RAPS	0.95 (0.94 to 0.95)	<0.001		
Lower BMI	Major depressive disorder	Men	249	IVW	0.98 (0.97 to 0.99)	0.003		<0.001
Lower BMI	Major depressive disorder	Men	249	Weighted median	0.98 (0.96 to 0.99)	0.01		
Lower BMI	Major depressive disorder	Men	249	MR Egger	0.98 (0.94 to 1.02)	0.35	0.99	
Lower BMI	Major depressive disorder	Men	249	MR-RAPS	0.98 (0.97 to 0.99)	<0.001		
Lower BMI	Post-partum depression	Women	300	IVW	0.98 (0.96 to 1.00)	0.03		<0.001
Lower BMI	Post-partum depression	Women	300	Weighted median	0.99 (0.96 to 1.02)	0.45		
Lower BMI	Post-partum depression	Women	300	MR Egger	0.98 (0.93 to 1.04)	0.57	0.81	
Lower BMI	Post-partum depression	Women	300	MR-RAPS	0.98 (0.96 to 1.00)	0.01		
Lower BMI	Bipolar disorder	Overall	506	IVW	1.00 (0.98 to 1.02)	0.77		<0.001
Lower BMI	Bipolar disorder	Overall	506	Weighted median	1.00 (0.98 to 1.03)	0.87		
Lower BMI	Bipolar disorder	Overall	506	MR Egger	0.97 (0.92 to 1.02)	0.27	0.19	
Lower BMI	Bipolar disorder	Overall	506	MR-RAPS	1.00 (0.99 to 1.01)	0.88		
Lower BMI	Bipolar disorder I	Overall	499	IVW	1.01 (0.98 to 1.03)	0.57		<0.001
Lower BMI	Bipolar disorder I	Overall	499	Weighted median	1.00 (0.97 to 1.03)	0.93		
Lower BMI	Bipolar disorder I	Overall	499	MR Egger	0.96 (0.90 to 1.02)	0.2	0.11	
Lower BMI	Bipolar disorder I	Overall	499	MR-RAPS	1.00 (0.98 to 1.02)	0.95		
Lower BMI	Bipolar disorder II	Overall	499	IVW	1.00 (0.97 to 1.03)	0.94		<0.001

Lower BMI	Bipolar disorder II	Overall	499	Weighted median	0.99 (0.95 to 1.04)	0.82		
Lower BMI	Bipolar disorder II	Overall	499	MR Egger	0.98 (0.90 to 1.05)	0.52	0.51	
Lower BMI	Bipolar disorder II	Overall	499	MR-RAPS	1.00 (0.97 to 1.02)	0.79		
Lower BMI	PTSD	Overall	506	IVW	0.94 (0.92 to 0.96)	<0.001		<0.001
Lower BMI	PTSD	Overall	506	Weighted median	0.94 (0.91 to 0.97)	<0.001		
Lower BMI	PTSD	Overall	506	MR Egger	0.96 (0.91 to 1.01)	0.13	0.36	
Lower BMI	PTSD	Overall	506	MR-RAPS	0.94 (0.92 to 0.95)	<0.001		
Lower BMI	Schizophrenia	Overall	505	IVW	1.03 (1.01 to 1.05)	0.01		<0.001
Lower BMI	Schizophrenia	Overall	505	Weighted median	1.00 (0.98 to 1.03)	0.72		
Lower BMI	Schizophrenia	Overall	505	MR Egger	0.93 (0.88 to 0.99)	0.02	<0.001	
Lower BMI	Schizophrenia	Overall	505	MR-RAPS	1.02 (1.01 to 1.03)	0.002		
Lower BMI	Schizophrenia	Overall	498	IVW (Steiger filtering)	1.03 (1.01 to 1.05)	0.004		<0.001
Lower BMI	Schizophrenia	Overall	498	Weighted median (Steiger filtering)	1.00 (0.98 to 1.03)	0.72		
Lower BMI	Schizophrenia	Overall	498	MR Egger (Steiger filtering)	0.95 (0.90 to 1.01)	0.09	0.003	
Lower BMI	Schizophrenia	Overall	498	MR-RAPS (Steiger filtering)	1.02 (1.01 to 1.03)	0.002		
Lower BMI	Schizophrenia	Women	300	IVW	1.01 (0.98 to 1.04)	0.57		<0.001
Lower BMI	Schizophrenia	Women	300	Weighted median	0.98 (0.94 to 1.02)	0.25		
Lower BMI	Schizophrenia	Women	300	MR Egger	0.90 (0.83 to 0.98)	0.01	0.003	
Lower BMI	Schizophrenia	Women	300	MR-RAPS	1.01 (0.99 to 1.03)	0.23		
Lower BMI	Schizophrenia	Women	297	IVW (Steiger filtering)	1.01 (0.98 to 1.04)	0.56		<0.001
Lower BMI	Schizophrenia	Women	297	Weighted median (Steiger filtering)	0.98 (0.94 to 1.02)	0.26		

Lower BMI	Schizophrenia	Women	297	MR Egger (Steiger filtering)	0.92 (0.85 to 0.99)	0.03	0.009	
Lower BMI	Schizophrenia	Women	297	MR-RAPS (Steiger filtering)	1.01 (0.99 to 1.03)	0.26		
Lower BMI	Schizophrenia	Men	252	IVW	1.01 (0.99 to 1.04)	0.39		<0.001
Lower BMI	Schizophrenia	Men	252	Weighted median	0.99 (0.96 to 1.02)	0.69		
Lower BMI	Schizophrenia	Men	252	MR Egger	0.97 (0.90 to 1.04)	0.32	0.16	
Lower BMI	Schizophrenia	Men	252	MR-RAPS	1.01 (0.99 to 1.02)	0.45		
Lower BMI	Schizophrenia	Men	251	IVW (Steiger filtering)	1.01 (0.98 to 1.03)	0.5		<0.001
Lower BMI	Schizophrenia	Men	251	Weighted median (Steiger filtering)	0.99 (0.96 to 1.02)	0.69		
Lower BMI	Schizophrenia	Men	251	MR Egger (Steiger filtering)	0.97 (0.91 to 1.04)	0.4	0.24	
Lower BMI	Schizophrenia	Men	251	MR-RAPS (Steiger filtering)	1.01 (0.99 to 1.02)	0.51		
Lower BMI	Anorexia nervosa	Overall	449	IVW	1.10 (1.07 to 1.12)	<0.001		<0.001
Lower BMI	Anorexia nervosa	Overall	449	Weighted median	1.07 (1.03 to 1.11)	<0.001		
Lower BMI	Anorexia nervosa	Overall	449	MR Egger	1.02 (0.96 to 1.09)	0.54	0.03	
Lower BMI	Anorexia nervosa	Overall	449	MR-RAPS	1.10 (1.08 to 1.12)	<0.001		
Lower BMI	Anorexia nervosa	Overall	448	IVW (Steiger filtering)	1.09 (1.06 to 1.12)	<0.001		<0.001
Lower BMI	Anorexia nervosa	Overall	448	Weighted median (Steiger filtering)	1.07 (1.03 to 1.10)	<0.001		
Lower BMI	Anorexia nervosa	Overall	448	MR Egger (Steiger filtering)	1.02 (0.95 to 1.09)	0.6	0.02	
Lower BMI	Anorexia nervosa	Overall	448	MR-RAPS (Steiger filtering)	1.10 (1.08 to 1.12)	<0.001		

Lower BMI	ADHD	Overall	496	IVW	0.89 (0.87 to 0.90)	<0.001		<0.001
Lower BMI	ADHD	Overall	496	Weighted median	0.90 (0.88 to 0.92)	<0.001		
Lower BMI	ADHD	Overall	496	MR Egger	0.95 (0.91 to 1.00)	0.04	0.001	
Lower BMI	ADHD	Overall	496	MR-RAPS	0.89 (0.88 to 0.90)	<0.001		
Lower BMI	ADHD	Women	257	IVW	0.88 (0.85 to 0.92)	<0.001		0.03
Lower BMI	ADHD	Women	257	Weighted median	0.91 (0.85 to 0.96)	0.001		
Lower BMI	ADHD	Women	257	MR Egger	0.92 (0.81 to 1.04)	0.2	0.49	
Lower BMI	ADHD	Women	257	MR-RAPS	0.89 (0.85 to 0.92)	<0.001		
Lower BMI	ADHD	Women	256	IVW (Steiger filtering)	0.89 (0.85 to 0.92)	<0.001		0.12
Lower BMI	ADHD	Women	256	Weighted median (Steiger filtering)	0.91 (0.85 to 0.96)	0.001		
Lower BMI	ADHD	Women	256	MR Egger (Steiger filtering)	0.92 (0.81 to 1.04)	0.16	0.58	
Lower BMI	ADHD	Women	256	MR-RAPS (Steiger filtering)	0.89 (0.86 to 0.93)	<0.001		
Lower BMI	ADHD	Men	215	IVW	0.88 (0.86 to 0.91)	<0.001		<0.001
Lower BMI	ADHD	Men	215	Weighted median	0.85 (0.82 to 0.89)	<0.001		
Lower BMI	ADHD	Men	215	MR Egger	0.89 (0.81 to 0.99)	0.03	0.84	
Lower BMI	ADHD	Men	215	MR-RAPS	0.88 (0.85 to 0.90)	<0.001		
Lower BMI	Autism spectrum disorder	Overall	453	IVW	0.98 (0.95 to 1.00)	0.05		<0.001
Lower BMI	Autism spectrum disorder	Overall	453	Weighted median	0.98 (0.94 to 1.01)	0.17		
Lower BMI	Autism spectrum disorder	Overall	453	MR Egger	0.98 (0.92 to 1.04)	0.51	0.9	
Lower BMI	Autism spectrum disorder	Overall	453	MR-RAPS	0.97 (0.95 to 0.99)	0.002		
Lower BMI	Autism spectrum disorder	Overall	450	IVW (Steiger filtering)	0.97 (0.95 to 0.99)	0.01		<0.001
Lower BMI	Autism spectrum disorder	Overall	450	Weighted median (Steiger filtering)	0.97 (0.94 to 1.01)	0.11		

Lower BMI	Autism spectrum disorder	Overall	450	MR Egger (Steiger filtering)	0.98 (0.93 to 1.04)	0.54	0.68	
Lower BMI	Autism spectrum disorder	Overall	450	MR-RAPS (Steiger filtering)	0.97 (0.95 to 0.99)	<0.001		
Lower BMI	Tourette syndrome	Overall	451	IVW	0.97 (0.93 to 1.01)	0.14		<0.001
Lower BMI	Tourette syndrome	Overall	451	Weighted median	0.96 (0.90 to 1.03)	0.22		
Lower BMI	Tourette syndrome	Overall	451	MR Egger	1.03 (0.93 to 1.15)	0.56	0.21	
Lower BMI	Tourette syndrome	Overall	451	MR-RAPS	0.97 (0.93 to 1.00)	0.09		
Lower BMI	Tourette syndrome	Overall	444	IVW (Steiger filtering)	0.98 (0.94 to 1.02)	0.28		<0.001
Lower BMI	Tourette syndrome	Overall	444	Weighted median (Steiger filtering)	0.96 (0.90 to 1.03)	0.24		
Lower BMI	Tourette syndrome	Overall	444	MR Egger (Steiger filtering)	1.03 (0.93 to 1.14)	0.63	0.33	
Lower BMI	Tourette syndrome	Overall	444	MR-RAPS (Steiger filtering)	0.97 (0.94 to 1.01)	0.14		
Lower HbA1c	Major depressive disorder	Overall	282	IVW	1.00 (0.99 to 1.00)	0.14		<0.001
Lower HbA1c	Major depressive disorder	Overall	282	Weighted median	1.00 (0.99 to 1.00)	0.01		
Lower HbA1c	Major depressive disorder	Overall	282	MR Egger	1.00 (0.99 to 1.00)	0.56	0.79	
Lower HbA1c	Major depressive disorder	Overall	282	MR-RAPS	1.00 (0.99 to 1.00)	0.003		
Lower HbA1c	Major depressive disorder	Women	185	IVW	1.00 (0.99 to 1.00)	0.27		<0.001
Lower HbA1c	Major depressive disorder	Women	185	Weighted median	1.00 (0.99 to 1.01)	0.94		
Lower HbA1c	Major depressive disorder	Women	185	MR Egger	1.00 (0.99 to 1.01)	0.56	0.13	
Lower HbA1c	Major depressive disorder	Women	185	MR-RAPS	1.00 (0.99 to 1.00)	0.13		
Lower HbA1c	Major depressive disorder	Men	160	IVW	1.00 (0.99 to 1.01)	0.9		0.005
Lower HbA1c	Major depressive disorder	Men	160	Weighted median	1.00 (0.99 to 1.01)	0.5		
Lower HbA1c	Major depressive disorder	Men	160	MR Egger	1.00 (0.99 to 1.01)	0.73	0.74	
Lower HbA1c	Major depressive disorder	Men	160	MR-RAPS	1.00 (0.99 to 1.00)	0.87		

Lower HbA1c	Post-partum depression	Women	192	IVW	1.00 (0.99 to 1.01)	0.41		0.002
Lower HbA1c	Post-partum depression	Women	192	Weighted median	0.99 (0.98 to 1.01)	0.49		
Lower HbA1c	Post-partum depression	Women	192	MR Egger	1.00 (0.98 to 1.02)	0.82	0.74	
Lower HbA1c	Post-partum depression	Women	192	MR-RAPS	1.00 (0.99 to 1.01)	0.42		
Lower HbA1c	Bipolar disorder	Overall	286	IVW	1.01 (0.99 to 1.02)	0.34		<0.001
Lower HbA1c	Bipolar disorder	Overall	286	Weighted median	1.00 (0.99 to 1.01)	0.54		
Lower HbA1c	Bipolar disorder	Overall	286	MR Egger	0.99 (0.97 to 1.01)	0.17	0.02	
Lower HbA1c	Bipolar disorder	Overall	286	MR-RAPS	1.00 (1.00 to 1.01)	0.6		
Lower HbA1c	Bipolar disorder I	Overall	284	IVW	1.01 (1.00 to 1.02)	0.25		<0.001
Lower HbA1c	Bipolar disorder I	Overall	284	Weighted median	1.00 (0.99 to 1.02)	0.66		
Lower HbA1c	Bipolar disorder I	Overall	284	MR Egger	0.99 (0.96 to 1.01)	0.18	0.02	
Lower HbA1c	Bipolar disorder I	Overall	284	MR-RAPS	1.00 (1.00 to 1.01)	0.28		
Lower HbA1c	Bipolar disorder II	Overall	283	IVW	1.00 (0.99 to 1.02)	0.86		0.002
Lower HbA1c	Bipolar disorder II	Overall	283	Weighted median	1.00 (0.97 to 1.02)	0.8		
Lower HbA1c	Bipolar disorder II	Overall	283	MR Egger	0.97 (0.95 to 1.00)	0.06	0.02	
Lower HbA1c	Bipolar disorder II	Overall	283	MR-RAPS	1.00 (0.99 to 1.02)	0.5		
Lower HbA1c	PTSD	Overall	300	IVW	1.00 (0.99 to 1.01)	0.64		0.43
Lower HbA1c	PTSD	Overall	300	Weighted median	1.02 (1.00 to 1.03)	0.07		
Lower HbA1c	PTSD	Overall	300	MR Egger	1.02 (1.01 to 1.04)	0.01	0.008	
Lower HbA1c	PTSD	Overall	300	MR-RAPS	1.00 (0.99 to 1.02)	0.36		
Lower HbA1c	Schizophrenia	Overall	287	IVW	1.00 (0.99 to 1.01)	0.45		<0.001
Lower HbA1c	Schizophrenia	Overall	287	Weighted median	1.00 (0.98 to 1.01)	0.37		
Lower HbA1c	Schizophrenia	Overall	287	MR Egger	0.99 (0.97 to 1.01)	0.17	0.03	
Lower HbA1c	Schizophrenia	Overall	287	MR-RAPS	1.00 (0.99 to 1.01)	0.95		
Lower HbA1c	Schizophrenia	Overall	286	IVW (Steiger filtering)	1.00 (0.99 to 1.01)	0.53		<0.001
Lower HbA1c	Schizophrenia	Overall	286	Weighted median (Steiger filtering)	1.00 (0.98 to 1.01)	0.36		

Lower HbA1c	Schizophrenia	Overall	286	MR Egger (Steiger filtering)	0.99 (0.97 to 1.01)	0.23	0.06	
Lower HbA1c	Schizophrenia	Overall	286	MR-RAPS (Steiger filtering)	1.00 (0.99 to 1.01)	1		
Lower HbA1c	Schizophrenia	Women	184	IVW	0.99 (0.98 to 1.01)	0.3		<0.001
Lower HbA1c	Schizophrenia	Women	184	Weighted median	0.98 (0.97 to 1.00)	0.05		
Lower HbA1c	Schizophrenia	Women	184	MR Egger	0.99 (0.97 to 1.01)	0.33	0.64	
Lower HbA1c	Schizophrenia	Women	184	MR-RAPS	1.00 (0.99 to 1.00)	0.33		
Lower HbA1c	Schizophrenia	Men	157	IVW	1.00 (0.99 to 1.01)	0.86		<0.001
Lower HbA1c	Schizophrenia	Men	157	Weighted median	1.00 (0.98 to 1.01)	0.46		
Lower HbA1c	Schizophrenia	Men	157	MR Egger	0.99 (0.97 to 1.02)	0.59	0.45	
Lower HbA1c	Schizophrenia	Men	157	MR-RAPS	1.00 (0.99 to 1.01)	0.8		
Lower HbA1c	Schizophrenia	Men	156	IVW (Steiger filtering)	1.00 (0.99 to 1.01)	0.93		<0.001
Lower HbA1c	Schizophrenia	Men	156	Weighted median (Steiger filtering)	1.00 (0.98 to 1.01)	0.46		
Lower HbA1c	Schizophrenia	Men	156	MR Egger (Steiger filtering)	1.00 (0.98 to 1.02)	0.72	0.71	
Lower HbA1c	Schizophrenia	Men	156	MR-RAPS (Steiger filtering)	1.00 (0.99 to 1.01)	0.87		
Lower HbA1c	Anorexia nervosa	Overall	246	IVW	1.02 (1.00 to 1.03)	0.02		<0.001
Lower HbA1c	Anorexia nervosa	Overall	246	Weighted median	1.01 (0.99 to 1.02)	0.53		
Lower HbA1c	Anorexia nervosa	Overall	246	MR Egger	1.01 (0.99 to 1.03)	0.42	0.6	
Lower HbA1c	Anorexia nervosa	Overall	246	MR-RAPS	1.02 (1.00 to 1.03)	0.005		
Lower HbA1c	ADHD	Overall	270	IVW	0.99 (0.98 to 1.00)	0.02		<0.001
Lower HbA1c	ADHD	Overall	270	Weighted median	0.99 (0.98 to 1.01)	0.28		
Lower HbA1c	ADHD	Overall	270	MR Egger	1.01 (0.99 to 1.02)	0.46	0.01	
Lower HbA1c	ADHD	Overall	270	MR-RAPS	0.99 (0.98 to 1.00)	0.004		

Lower HbA1c	ADHD	Women	150	IVW	1.00 (0.98 to 1.02)	0.92		0.71
Lower HbA1c	ADHD	Women	150	Weighted median	1.01 (0.97 to 1.04)	0.75		
Lower HbA1c	ADHD	Women	150	MR Egger	1.01 (0.98 to 1.04)	0.6	0.56	
Lower HbA1c	ADHD	Women	150	MR-RAPS	1.00 (0.98 to 1.02)	0.8		
Lower HbA1c	ADHD	Men	133	IVW	0.99 (0.97 to 1.00)	0.12		<0.001
Lower HbA1c	ADHD	Men	133	Weighted median	0.99 (0.96 to 1.01)	0.21		
Lower HbA1c	ADHD	Men	133	MR Egger	0.99 (0.97 to 1.02)	0.63	0.66	
Lower HbA1c	ADHD	Men	133	MR-RAPS	0.99 (0.98 to 1.00)	0.05		
Lower HbA1c	Autism spectrum disorder	Overall	250	IVW	1.00 (0.99 to 1.02)	0.43		0.02
Lower HbA1c	Autism spectrum disorder	Overall	250	Weighted median	1.01 (1.00 to 1.03)	0.11		
Lower HbA1c	Autism spectrum disorder	Overall	250	MR Egger	1.01 (0.99 to 1.03)	0.59	0.9	
Lower HbA1c	Autism spectrum disorder	Overall	250	MR-RAPS	1.00 (0.99 to 1.02)	0.42		
Lower HbA1c	Autism spectrum disorder	Overall	249	IVW (Steiger filtering)	1.00 (0.99 to 1.01)	0.57		0.19
Lower HbA1c	Autism spectrum disorder	Overall	249	Weighted median (Steiger filtering)	1.01 (1.00 to 1.03)	0.1		
Lower HbA1c	Autism spectrum disorder	Overall	249	MR Egger (Steiger filtering)	1.01 (0.99 to 1.03)	0.52	0.69	
Lower HbA1c	Autism spectrum disorder	Overall	249	MR-RAPS (Steiger filtering)	1.00 (0.99 to 1.01)	0.47		
Lower HbA1c	Tourette syndrome	Overall	255	IVW	0.99 (0.97 to 1.01)	0.18		0.46
Lower HbA1c	Tourette syndrome	Overall	255	Weighted median	0.99 (0.96 to 1.02)	0.37		
Lower HbA1c	Tourette syndrome	Overall	255	MR Egger	1.00 (0.97 to 1.04)	0.96	0.35	
Lower HbA1c	Tourette syndrome	Overall	255	MR-RAPS	0.99 (0.97 to 1.01)	0.31		

ADHD, attention deficit hyperactivity disorder; BMI, body mass index; HbA1c, glycated hemoglobin; IVW, inverse variance weighting; MR, Mendelian randomization; PTSD, post-traumatic stress disorder; RAPS, robust adjusted profile score. Estimates are presented as odds ratio per 1-kg/m^2 decrease in BMI or per 1-mmol/mol decrease in HbA1c.

eTable 9. MR sensitivity analyses for the associations of lower BMI and lower HbA1c with the risk of substance use disorders.

Exposure	Outcome	SNPs	Method	Estimate (95% CI)	Measure	*P* value	*P* value (intercept)	*P* value (Q)
Lower BMI	Substance use disorders	359	IVW	-0.01 (-0.02 to -0.01)	Beta	<0.001		<0.001
Lower BMI	Substance use disorders	359	Weighted median	-0.01 (-0.02 to -0.01)	Beta	<0.001		
Lower BMI	Substance use disorders	359	MR Egger	0.003 (-0.01 to 0.02)	Beta	0.70	0.02	
Lower BMI	Substance use disorders	359	MR-RAPS	-0.01 (-0.02 to -0.01)	Beta	<0.001		
Lower BMI	Cannabis use disorder	453	IVW	0.94 (0.92 to 0.97)	Odds ratio	<0.001		<0.001
Lower BMI	Cannabis use disorder	453	Weighted median	0.94 (0.90 to 0.98)	Odds ratio	0.01		
Lower BMI	Cannabis use disorder	453	MR Egger	0.99 (0.92 to 1.06)	Odds ratio	0.72	0.19	
Lower BMI	Cannabis use disorder	453	MR-RAPS	0.94 (0.92 to 0.97)	Odds ratio	<0.001		
Lower BMI	Alcohol dependence	452	IVW	0.99 (0.95 to 1.02)	Odds ratio	0.35		0.14
Lower BMI	Alcohol dependence	452	Weighted median	1.00 (0.95 to 1.06)	Odds ratio	0.94		
Lower BMI	Alcohol dependence	452	MR Egger	1.04 (0.96 to 1.13)	Odds ratio	0.34	0.15	
Lower BMI	Alcohol dependence	452	MR-RAPS	0.99 (0.96 to 1.02)	Odds ratio	0.39		
Lower BMI	Alcohol dependence	451	IVW (Steiger filtering)	0.99 (0.96 to 1.02)	Odds ratio	0.44		0.26
Lower BMI	Alcohol dependence	451	Weighted median (Steiger filtering)	1.00 (0.95 to 1.06)	Odds ratio	0.93		
Lower BMI	Alcohol dependence	451	MR Egger (Steiger filtering)	1.04 (0.96 to 1.13)	Odds ratio	0.31	0.15	
Lower BMI	Alcohol dependence	451	MR-RAPS (Steiger filtering)	0.99 (0.96 to 1.02)	Odds ratio	0.43		
Lower BMI	AUDIT (total score)	452	IVW	0.003 (0.001 to 0.01)	Beta	0.01		<0.001
Lower BMI	AUDIT (total score)	452	Weighted median	0.002 (-0.001 to 0.005)	Beta	0.27		
Lower BMI	AUDIT (total score)	452	MR Egger	0.002 (-0.004 to 0.01)	Beta	0.53	0.71	
Lower BMI	AUDIT (total score)	452	MR-RAPS	0.002 (0.001 to 0.004)	Beta	0.01		

Lower BMI	AUDIT (total score)	451	IVW (Steiger filtering)	0.003 (0.001 to 0.01)	Beta	0.01		<0.001
Lower BMI	AUDIT (total score)	451	Weighted median (Steiger filtering)	0.002 (-0.001 to 0.005)	Beta	0.27		
Lower BMI	AUDIT (total score)	451	MR Egger (Steiger filtering)	0.001 (-0.004 to 0.01)	Beta	0.63	0.54	
Lower BMI	AUDIT (total score)	451	MR-RAPS (Steiger filtering)	0.002 (0.001 to 0.004)	Beta	0.01		
Lower BMI	AUDIT (alcohol consumption)	452	IVW	0.003 (0.001 to 0.01)	Beta	0.001		<0.001
Lower BMI	AUDIT (alcohol consumption)	452	Weighted median	0.002 (-0.001 to 0.01)	Beta	0.15		
Lower BMI	AUDIT (alcohol consumption)	452	MR Egger	0.001 (-0.004 to 0.01)	Beta	0.60	0.43	
Lower BMI	AUDIT (alcohol consumption)	452	MR-RAPS	0.003 (0.001 to 0.004)	Beta	<0.001		
Lower BMI	AUDIT (alcohol consumption)	451	IVW (Steiger filtering)	0.004 (0.002 to 0.01)	Beta	<0.001		<0.001
Lower BMI	AUDIT (alcohol consumption)	451	Weighted median (Steiger filtering)	0.002 (-0.001 to 0.01)	Beta	0.14		
Lower BMI	AUDIT (alcohol consumption)	451	MR Egger (Steiger filtering)	0.001 (-0.004 to 0.01)	Beta	0.70	0.30	
Lower BMI	AUDIT (alcohol consumption)	451	MR-RAPS (Steiger filtering)	0.003 (0.001 to 0.004)	Beta	<0.001		
Lower BMI	AUDIT (alcohol problems)	452	IVW	-0.001 (-0.003 to 0.001)	Beta	0.16		<0.001
Lower BMI	AUDIT (alcohol problems)	452	Weighted median	0.000 (-0.002 to 0.002)	Beta	0.99		
Lower BMI	AUDIT (alcohol problems)	452	MR Egger	0.000 (-0.005 to 0.005)	Beta	0.98	0.54	
Lower BMI	AUDIT (alcohol problems)	452	MR-RAPS	-0.001 (-0.003 to 0.000)	Beta	0.11		
Lower BMI	AUDIT (alcohol problems)	451	IVW (Steiger filtering)	-0.001 (-0.003 to 0.001)	Beta	0.20		<0.001
Lower BMI	AUDIT (alcohol problems)	451	Weighted median (Steiger filtering)	0.000 (-0.002 to 0.002)	Beta	0.99		

Lower BMI	AUDIT (alcohol problems)	451	MR Egger (Steiger filtering)	-0.000 (-0.005 to 0.004)	Beta	0.91	0.67	
Lower BMI	AUDIT (alcohol problems)	451	MR-RAPS (Steiger filtering)	-0.001 (-0.003 to 0.000)	Beta	0.13		
Lower HbA1c	Substance use disorders	192	IVW	-0.001 (-0.004 to 0.002)	Beta	0.49		<0.001
Lower HbA1c	Substance use disorders	192	Weighted median	-0.000 (-0.003 to 0.003)	Beta	0.83		
Lower HbA1c	Substance use disorders	192	MR Egger	-0.000 (-0.01 to 0.005)	Beta	0.87	0.80	
Lower HbA1c	Substance use disorders	192	MR-RAPS	-0.001 (-0.003 to 0.001)	Beta	0.23		
Lower HbA1c	Cannabis use disorder	253	IVW	0.99 (0.98 to 1.00)	Odds ratio	0.17		0.02
Lower HbA1c	Cannabis use disorder	253	Weighted median	0.99 (0.97 to 1.01)	Odds ratio	0.27		
Lower HbA1c	Cannabis use disorder	253	MR Egger	1.00 (0.97 to 1.02)	Odds ratio	0.92	0.45	
Lower HbA1c	Cannabis use disorder	253	MR-RAPS	0.99 (0.98 to 1.00)	Odds ratio	0.08		
Lower HbA1c	Alcohol dependence	252	IVW	1.00 (0.98 to 1.02)	Odds ratio	0.86		0.16
Lower HbA1c	Alcohol dependence	252	Weighted median	1.01 (0.98 to 1.04)	Odds ratio	0.69		
Lower HbA1c	Alcohol dependence	252	MR Egger	1.02 (0.98 to 1.05)	Odds ratio	0.36	0.33	
Lower HbA1c	Alcohol dependence	252	MR-RAPS	1.00 (0.98 to 1.02)	Odds ratio	0.90		
Lower HbA1c	AUDIT (total score)	262	IVW	0.001 (0.000 to 0.002)	Beta	0.02		<0.001
Lower HbA1c	AUDIT (total score)	262	Weighted median	0.000 (-0.001 to 0.002)	Beta	0.62		
Lower HbA1c	AUDIT (total score)	262	MR Egger	-0.001 (-0.003 to 0.001)	Beta	0.44	0.01	
Lower HbA1c	AUDIT (total score)	262	MR-RAPS	0.001 (0.000 to 0.002)	Beta	0.01		
Lower HbA1c	AUDIT (total score)	261	IVW (Steiger filtering)	0.001 (0.000 to 0.002)	Beta	0.02		<0.001
Lower HbA1c	AUDIT (total score)	261	Weighted median (Steiger filtering)	0.000 (-0.001 to 0.002)	Beta	0.62		
Lower HbA1c	AUDIT (total score)	261	MR Egger (Steiger filtering)	-0.001 (-0.002 to 0.001)	Beta	0.59	0.03	
Lower HbA1c	AUDIT (total score)	261	MR-RAPS (Steiger filtering)	0.001 (0.000 to 0.002)	Beta	0.01		

Lower HbA1c	AUDIT (alcohol consumption)	262	IVW	0.001 (0.000 to 0.002)	Beta	0.01		<0.001
Lower HbA1c	AUDIT (alcohol consumption)	262	Weighted median	0.000 (-0.001 to 0.002)	Beta	0.71		
Lower HbA1c	AUDIT (alcohol consumption)	262	MR Egger	-0.001 (-0.002 to 0.001)	Beta	0.50	0.01	
Lower HbA1c	AUDIT (alcohol consumption)	262	MR-RAPS	0.001 (0.000 to 0.002)	Beta	0.001		
Lower HbA1c	AUDIT (alcohol consumption)	261	IVW (Steiger filtering)	0.001 (0.000 to 0.002)	Beta	0.01		<0.001
Lower HbA1c	AUDIT (alcohol consumption)	261	Weighted median (Steiger filtering)	0.000 (-0.001 to 0.002)	Beta	0.71		
Lower HbA1c	AUDIT (alcohol consumption)	261	MR Egger (Steiger filtering)	-0.000 (-0.002 to 0.001)	Beta	0.65	0.02	
Lower HbA1c	AUDIT (alcohol consumption)	261	MR-RAPS (Steiger filtering)	0.001 (0.000 to 0.002)	Beta	0.002		
Lower HbA1c	AUDIT (alcohol problems)	262	IVW	0.000 (-0.001 to 0.001)	Beta	0.78		0.002
Lower HbA1c	AUDIT (alcohol problems)	262	Weighted median	-0.000 (-0.001 to 0.001)	Beta	0.81		
Lower HbA1c	AUDIT (alcohol problems)	262	MR Egger	-0.001 (-0.002 to 0.001)	Beta	0.34	0.18	
Lower HbA1c	AUDIT (alcohol problems)	262	MR-RAPS	0.000 (-0.001 to 0.001)	Beta	0.65		

AUDIT, Alcohol Use Disorders Identification Test; BMI, body mass index; HbA1c, glycated hemoglobin; IVW, inverse variance weighting; MR, Mendelian randomization; RAPS, robust adjusted profile score. Positive associations with AUDIT scores indicate higher risk of alcohol use disorders. Estimates are presented per 1-kg/m2 decrease in BMI or per 1-mmol/mol decrease in HbA1c.

eFigure 6. IVW MR estimates for the associations of GLP-1R activation with mental health outcomes in comparison with those for lower BMI and lower HbA1c in FinnGen.

(a) BMI lowering | **(b) HbA1c lowering**

■ Lower BMI via GLP-1R activation ⊖ Lower BMI | ■ Lower HbA1c via GLP-1R activation ⊖ Lower HbA1c

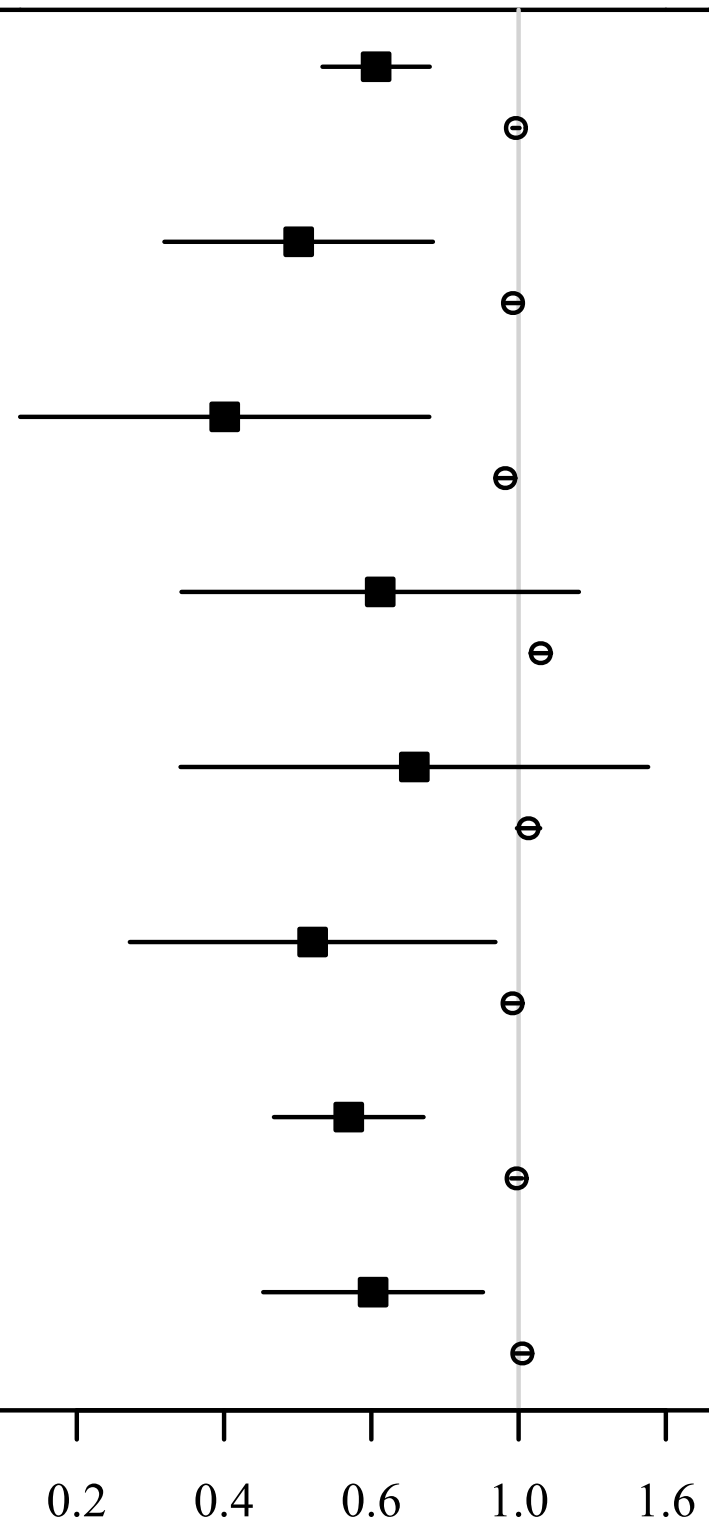

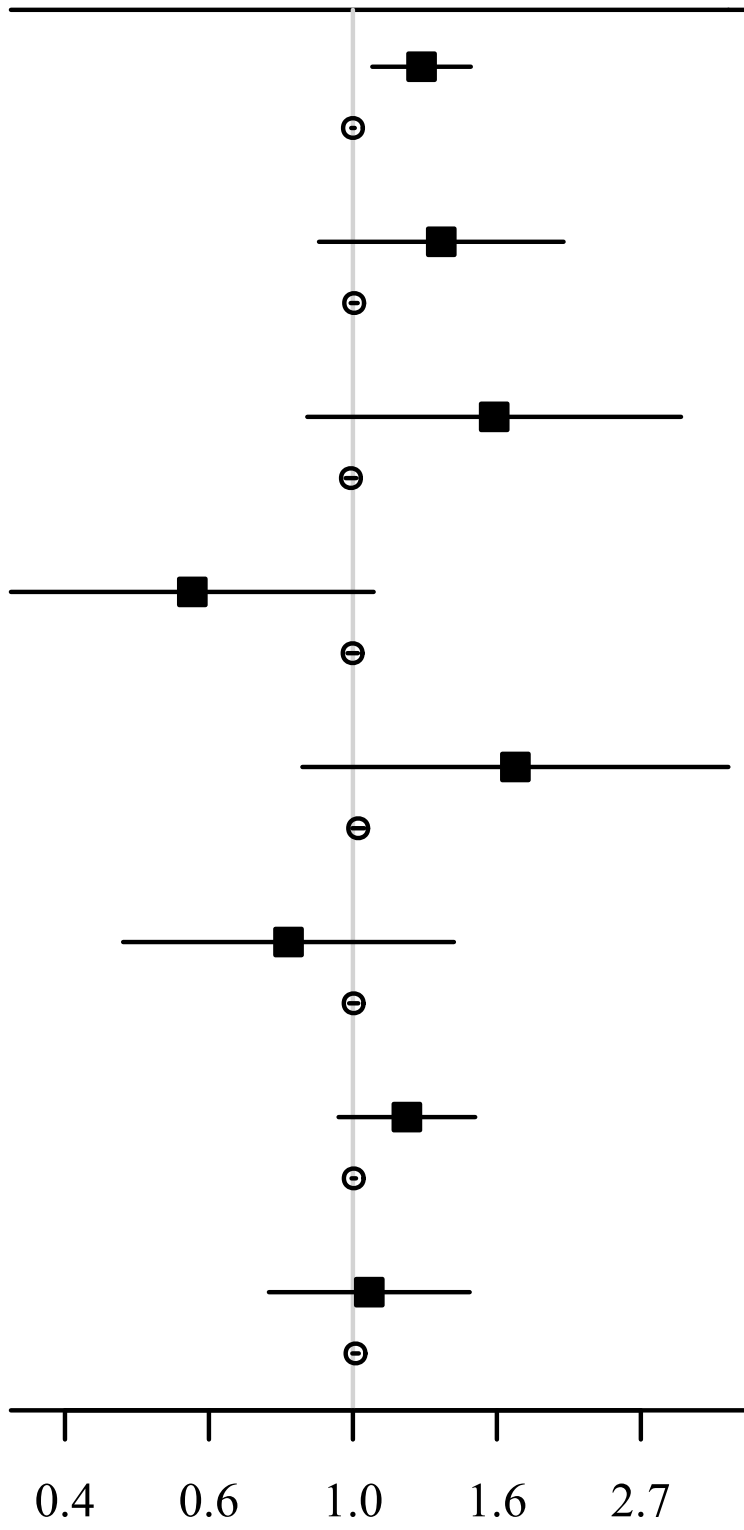

Outcome	Case/control	(a) Odds ratio (95% CI)	(a) P value	(b) Odds ratio (95% CI)	(b) P value
Major depressive disorder	59,333/434,831	0.62 (0.51 to 0.74)	<.001	1.27 (1.07 to 1.51)	.006
		0.99 (0.98 to 1.00)	.15	1.00 (0.99 to 1.01)	.92
Bipolar disorder	8,946/434,831	0.47 (0.30 to 0.75)	.001	1.36 (0.89 to 2.08)	.16
		0.98 (0.96 to 1.01)	.12	1.00 (0.99 to 1.02)	.48
PTSD	3,444/444,414	0.37 (0.18 to 0.74)	.005	1.63 (0.85 to 3.13)	.14
		0.96 (0.92 to 0.99)	.009	0.99 (0.98 to 1.01)	.47
Schizophrenia	7,234/484,776	0.63 (0.32 to 1.23)	.17	0.57 (0.30 to 1.07)	.08
		1.08 (1.04 to 1.12)	<.001	1.00 (0.98 to 1.02)	.93
Anorexia nervosa	2,621/484,901	0.70 (0.32 to 1.55)	.38	1.76 (0.84 to 3.68)	.13
		1.03 (0.99 to 1.08)	.09	1.02 (1.00 to 1.04)	.08
ADHD	4,452/490,708	0.50 (0.27 to 0.92)	.03	0.80 (0.45 to 1.42)	.45
		0.98 (0.95 to 1.01)	.21	1.00 (0.99 to 1.02)	.74
Substance abuse	30,806/362,304	0.56 (0.44 to 0.72)	<.001	1.21 (0.95 to 1.53)	.12
		0.99 (0.98 to 1.01)	.48	1.00 (1.00 to 1.01)	.45
Alcohol dependence	12,599/468,475	0.61 (0.42 to 0.89)	.009	1.06 (0.75 to 1.50)	.75
		1.01 (0.99 to 1.04)	.30	1.01 (1.00 to 1.02)	.11

ADHD, attention deficit hyperactivity disorder; BMI, body mass index; GLP-1R, glucagon-like peptide-1 receptor; HbA1c, glycated hemoglobin; IVW, inverse variance weighted; MR, Mendelian randomization; PTSD, post-traumatic stress disorder. Black squares denote genetically predicted (a) lower BMI or (b) lower HbA1c via GLP-1R activation based on variants within 1 Mb of *GLP1R*. White circles denote genetically predicted (a) lower BMI or (b) lower HbA1c based on genome-wide variants. Estimates are presented as odds ratio per 1-kg/m^2 decrease in BMI or per 1-mmol/mol decrease in HbA1c.

eTable 10. MR sensitivity analyses for the associations of lower BMI and lower HbA1c with mental health outcomes in FinnGen.

Exposure	Outcome	SNPs	Method	Odds ratio (95% CI)	*P* value	*P* value (intercept)	*P* value (Q)
Lower BMI	Major depressive disorder	500	IVW	0.99 (0.98 to 1.00)	0.15		<0.001
Lower BMI	Major depressive disorder	500	Weighted median	1.00 (0.99 to 1.02)	0.85		
Lower BMI	Major depressive disorder	500	MR Egger	1.01 (0.97 to 1.04)	0.71	0.32	
Lower BMI	Major depressive disorder	500	MR-RAPS	1.00 (0.99 to 1.00)	0.39		
Lower BMI	Bipolar disorder	500	IVW	0.98 (0.96 to 1.01)	0.12		<0.001
Lower BMI	Bipolar disorder	500	Weighted median	0.97 (0.94 to 1.01)	0.17		
Lower BMI	Bipolar disorder	500	MR Egger	0.99 (0.93 to 1.06)	0.76	0.77	
Lower BMI	Bipolar disorder	500	MR-RAPS	0.98 (0.96 to 1.01)	0.16		
Lower BMI	PTSD	500	IVW	0.96 (0.92 to 0.99)	0.009		0.008
Lower BMI	PTSD	500	Weighted median	1.00 (0.95 to 1.06)	0.97		
Lower BMI	PTSD	500	MR Egger	1.07 (0.98 to 1.17)	0.12	0.006	
Lower BMI	PTSD	500	MR-RAPS	0.97 (0.93 to 1.00)	0.03		
Lower BMI	Schizophrenia	500	IVW	1.08 (1.04 to 1.12)	<0.001		<0.001
Lower BMI	Schizophrenia	500	Weighted median	1.10 (1.04 to 1.17)	<0.001		
Lower BMI	Schizophrenia	500	MR Egger	1.02 (0.93 to 1.11)	0.74	0.17	
Lower BMI	Schizophrenia	500	MR-RAPS	1.09 (1.05 to 1.12)	<0.001		
Lower BMI	Anorexia nervosa	500	IVW	1.03 (0.99 to 1.08)	0.09		0.001
Lower BMI	Anorexia nervosa	500	Weighted median	1.05 (0.99 to 1.11)	0.12		
Lower BMI	Anorexia nervosa	500	MR Egger	1.08 (0.97 to 1.20)	0.16	0.4	
Lower BMI	Anorexia nervosa	500	MR-RAPS	1.03 (1.00 to 1.07)	0.08		
Lower BMI	ADHD	500	IVW	0.98 (0.95 to 1.01)	0.21		<0.001
Lower BMI	ADHD	500	Weighted median	0.99 (0.94 to 1.05)	0.78		
Lower BMI	ADHD	500	MR Egger	0.98 (0.90 to 1.07)	0.62	0.97	
Lower BMI	ADHD	500	MR-RAPS	0.98 (0.96 to 1.01)	0.29		
Lower BMI	Substance abuse	500	IVW	0.99 (0.98 to 1.01)	0.48		<0.001

Lower BMI	Substance abuse	500	Weighted median	1.01 (0.99 to 1.04)	0.23		
Lower BMI	Substance abuse	500	MR Egger	1.08 (1.03 to 1.13)	0.002	<0.001	
Lower BMI	Substance abuse	500	MR-RAPS	1.00 (0.98 to 1.01)	0.45		
Lower BMI	Alcohol dependence	500	IVW	1.01 (0.99 to 1.04)	0.3		<0.001
Lower BMI	Alcohol dependence	500	Weighted median	1.04 (1.01 to 1.08)	0.005		
Lower BMI	Alcohol dependence	500	MR Egger	1.09 (1.02 to 1.16)	0.008	0.01	
Lower BMI	Alcohol dependence	500	MR-RAPS	1.01 (1.00 to 1.03)	0.1		
Lower HbA1c	Major depressive disorder	291	IVW	1.00 (0.99 to 1.01)	0.92		<0.001
Lower HbA1c	Major depressive disorder	291	Weighted median	1.00 (0.99 to 1.01)	0.52		
Lower HbA1c	Major depressive disorder	291	MR Egger	1.00 (0.99 to 1.01)	0.76	0.77	
Lower HbA1c	Major depressive disorder	291	MR-RAPS	1.00 (1.00 to 1.01)	0.67		
Lower HbA1c	Bipolar disorder	291	IVW	1.00 (0.99 to 1.02)	0.48		0.006
Lower HbA1c	Bipolar disorder	291	Weighted median	1.01 (0.99 to 1.03)	0.17		
Lower HbA1c	Bipolar disorder	291	MR Egger	1.01 (0.99 to 1.03)	0.35	0.52	
Lower HbA1c	Bipolar disorder	291	MR-RAPS	1.00 (0.99 to 1.02)	0.43		
Lower HbA1c	PTSD	291	IVW	0.99 (0.98 to 1.01)	0.47		0.03
Lower HbA1c	PTSD	291	Weighted median	0.99 (0.97 to 1.02)	0.63		
Lower HbA1c	PTSD	291	MR Egger	1.01 (0.97 to 1.04)	0.66	0.32	
Lower HbA1c	PTSD	291	MR-RAPS	0.99 (0.98 to 1.01)	0.51		
Lower HbA1c	Schizophrenia	291	IVW	1.00 (0.98 to 1.02)	0.93		0.01
Lower HbA1c	Schizophrenia	291	Weighted median	0.99 (0.97 to 1.02)	0.68		
Lower HbA1c	Schizophrenia	291	MR Egger	0.99 (0.96 to 1.02)	0.5	0.45	
Lower HbA1c	Schizophrenia	291	MR-RAPS	1.00 (0.98 to 1.02)	0.88		
Lower HbA1c	Anorexia nervosa	291	IVW	1.02 (1.00 to 1.04)	0.08		0.04
Lower HbA1c	Anorexia nervosa	291	Weighted median	1.02 (0.99 to 1.06)	0.17		
Lower HbA1c	Anorexia nervosa	291	MR Egger	1.01 (0.97 to 1.05)	0.53	0.71	
Lower HbA1c	Anorexia nervosa	291	MR-RAPS	1.02 (1.00 to 1.04)	0.09		
Lower HbA1c	ADHD	291	IVW	1.00 (0.99 to 1.02)	0.74		0.16

Lower HbA1c	ADHD	291	Weighted median	1.00 (0.98 to 1.03)	0.93		
Lower HbA1c	ADHD	291	MR Egger	1.01 (0.98 to 1.04)	0.61	0.69	
Lower HbA1c	ADHD	291	MR-RAPS	1.00 (0.99 to 1.02)	0.56		
Lower HbA1c	Substance abuse	291	IVW	1.00 (1.00 to 1.01)	0.45		<0.001
Lower HbA1c	Substance abuse	291	Weighted median	1.00 (0.99 to 1.01)	0.75		
Lower HbA1c	Substance abuse	291	MR Egger	1.00 (0.99 to 1.02)	0.72	0.95	
Lower HbA1c	Substance abuse	291	MR-RAPS	1.00 (1.00 to 1.01)	0.44		
Lower HbA1c	Alcohol dependence	291	IVW	1.01 (1.00 to 1.02)	0.11		<0.001
Lower HbA1c	Alcohol dependence	291	Weighted median	1.00 (0.99 to 1.02)	0.78		
Lower HbA1c	Alcohol dependence	291	MR Egger	1.00 (0.98 to 1.02)	0.74	0.15	
Lower HbA1c	Alcohol dependence	291	MR-RAPS	1.01 (1.00 to 1.02)	0.16		

ADHD, attention deficit hyperactivity disorder; BMI, body mass index; HbA1c, glycated hemoglobin; IVW, inverse variance weighting; MR, Mendelian randomization; PTSD, post-traumatic stress disorder; RAPS, robust adjusted profile score. Estimates are presented as odds ratio per 1-kg/m^2 decrease in BMI or per 1-mmol/mol decrease in HbA1c.

eTable 11. Wald estimates for the associations of lower HbA1c via GLP-1R activation (instrumented by rs10305492) with coronary artery disease, all-cause mortality, and mental health outcomes.

Category	Outcome	Sex	Estimate (95% CI)	Measure	*P* value
Cardiovascular and mortality outcome	Coronary artery disease	Overall	0.94 (0.85 to 1.04)	Odds ratio	0.25
	All-cause mortality	Overall	0.96 (0.88 to 1.03)	Hazard ratio	0.27
Mental health disorder	Major depressive disorder	Overall	1.04 (0.99 to 1.10)	Odds ratio	0.13
	Major depressive disorder	Women	1.08 (0.92 to 1.27)	Odds ratio	0.36
	Major depressive disorder	Men	0.97 (0.87 to 1.08)	Odds ratio	0.59
	Post-partum depression	Women	0.98 (0.67 to 1.43)	Odds ratio	0.93
	Bipolar disorder	Overall	1.01 (0.83 to 1.22)	Odds ratio	0.92
	Bipolar disorder I	Overall	1.04 (0.79 to 1.37)	Odds ratio	0.77
	Bipolar disorder II	Overall	1.19 (0.75 to 1.90)	Odds ratio	0.46
	PTSD	Overall	1.32 (0.99 to 1.74)	Odds ratio	0.05
	Schizophrenia	Overall	1.06 (0.88 to 1.28)	Odds ratio	0.54
	Schizophrenia	Women	1.38 (0.88 to 2.17)	Odds ratio	0.16
	Schizophrenia	Men	1.00 (0.83 to 1.21)	Odds ratio	1.00
	Anorexia nervosa	Overall	1.07 (0.81 to 1.41)	Odds ratio	0.65
	ADHD	Overall	0.98 (0.80 to 1.19)	Odds ratio	0.83
	ADHD	Women	1.42 (0.68 to 2.95)	Odds ratio	0.35
	ADHD	Men	0.90 (0.70 to 1.15)	Odds ratio	0.39
	Autism spectrum disorder	Overall	1.21 (0.94 to 1.57)	Odds ratio	0.14
Substance use disorder	Cannabis use disorder	Overall	1.33 (0.93 to 1.91)	Odds ratio	0.12
	Alcohol dependence	Overall	1.03 (0.43 to 2.47)	Odds ratio	0.94
	AUDIT (total score)	Overall	0.01 (-0.02 to 0.03)	Beta	0.60
	AUDIT (alcohol consumption)	Overall	0.01 (-0.01 to 0.03)	Beta	0.55
	AUDIT (alcohol problems)	Overall	-0.004 (-0.02 to 0.02)	Beta	0.70
	Major depressive disorder	Overall	0.99 (0.87 to 1.14)	Odds ratio	0.93

Replication outcome (FinnGen)	Bipolar disorder	Overall	0.95 (0.68 to 1.33)	Odds ratio	0.76
	PTSD	Overall	1.03 (0.61 to 1.74)	Odds ratio	0.90
	Schizophrenia	Overall	1.11 (0.67 to 1.86)	Odds ratio	0.68
	Anorexia nervosa	Overall	0.86 (0.48 to 1.53)	Odds ratio	0.60
	ADHD	Overall	0.79 (0.50 to 1.25)	Odds ratio	0.31
	Substance abuse	Overall	0.91 (0.75 to 1.10)	Odds ratio	0.32
	Alcohol dependence	Overall	0.97 (0.73 to 1.28)	Odds ratio	0.80

ADHD, attention deficit hyperactivity disorder; AUDIT, Alcohol Use Disorders Identification Test; GLP-1R, glucagon-like peptide-1 receptor; HbA1c, glycated hemoglobin; PTSD, post-traumatic stress disorder. Estimates are presented per 1-mmol/mol decrease in HbA1c. rs10305492 was not available in the genome-wide association studies for the well-being spectrum, Tourette syndrome, and substance use disorders.

eTable 12. The posterior probabilities of different hypotheses in pairwise colocalization analyses for BMI and each significant outcome within 1 Mb of the *GLP1R* gene.

Outcome	SNPs	H_0	H_1	H_2	H_3	H_4	Conditional H_4
Well-being spectrum	2133	<0.1%	29.4%	<0.1%	11.3%	59.3%	84.0%
Life satisfaction	2133	<0.1%	13.4%	<0.1%	12.9%	73.8%	85.2%
Positive affect	2133	<0.1%	17.9%	<0.1%	14.0%	68.1%	82.9%
Neuroticism	2133	<0.1%	24.5%	<0.1%	14.4%	61.1%	80.9%
Depressive symptoms	2133	<0.1%	24.2%	<0.1%	13.0%	62.7%	82.8%
Major depressive disorder	2186	<0.1%	<0.1%	<0.1%	23.1%	76.9%	76.9%
Bipolar disorder	2375	<0.1%	50.0%	<0.1%	4.1%	45.9%	91.8%
Bipolar disorder I	2336	<0.1%	60.7%	<0.1%	4.5%	34.7%	88.5%

BMI, body mass index. H_0, no association with either trait; H_1, association with BMI only; H_2, association with outcome only; H_3, associations of two independent variants and one for each trait; H_4, associations of one shared variant with both traits; conditional H_4, associations of one shared variant with both traits conditional on the presence of a variant associated with outcome ($H_4/(H_2+H_3+H_4)$).
